# Development of Erasin: a chromone-based STAT3 inhibitor which induces apoptosis in Erlotinib-resistant lung cancer cells

**DOI:** 10.1038/s41598-017-17600-x

**Published:** 2017-12-12

**Authors:** Christian Lis, Stefan Rubner, Martin Roatsch, Angela Berg, Tyler Gilcrest, Darwin Fu, Elizabeth Nguyen, Anne-Marie Schmidt, Harald Krautscheid, Jens Meiler, Thorsten Berg

**Affiliations:** 1 Leipzig University, Institute of Organic Chemistry, Johannisallee 29, 04103 Leipzig, Germany; 2 Vanderbilt University, Center for Structural Biology, 465 21st Ave South, BIOSCI/MRBIII, Nashville, TN 37221 USA; 3 Leipzig University, Institute of Inorganic Chemistry, Johannisallee 29, 04103 Leipzig, Germany

## Abstract

Inhibition of protein-protein interactions by small molecules offers tremendous opportunities for basic research and drug development. One of the fundamental challenges of this research field is the broad lack of available lead structures from nature. Here, we demonstrate that modifications of a chromone-based inhibitor of the Src homology 2 (SH2) domain of the transcription factor STAT5 confer inhibitory activity against STAT3. The binding mode of the most potent STAT3 inhibitor Erasin was analyzed by the investigation of structure-activity relationships, which was facilitated by chemical synthesis and biochemical activity analysis, in combination with molecular docking studies. Erasin inhibits tyrosine phosphorylation of STAT3 with selectivity over STAT5 and STAT1 in cell-based assays, and increases the apoptotic rate of cultured NSCLC cells in a STAT3-dependent manner. This ability of Erasin also extends to HCC-827 cells with acquired resistance against Erlotinib, a clinically used inhibitor of the EGF receptor. Our work validates chromone-based acylhydrazones as privileged structures for antagonizing STAT SH2 domains, and demonstrates that apoptosis can be induced in NSCLC cells with acquired Erlotinib resistance by direct inhibition of STAT3.

## Introduction

Since protein-protein interactions mediate most biological processes, their inhibition by small molecules has tremendous potential for use in the study of cellular pathways, and for therapeutic interventions^1–5^. One of the fundamental difficulties associated with inhibition of protein-protein interactions, however, is the broad lack of known natural product-based lead structures. Natural products are an excellent source of structural inspiration for bioactive compounds, since they have co-evolved with the spectrum of animal and plant proteins^6^. Natural product-based scaffolds that can be tailored to target individual members of subclasses of protein-protein interaction domains would be of invaluable assistance in defining the chemical space of protein-protein interaction inhibitors.

STATs are a family of latent cytoplasmic transcription factors that carry signals from the cell surface to the nucleus^7,8^. All of the seven STAT family members known to date have been shown to play a role in human disease^8^. There is overwhelming biological evidence that targeting the transcription factors STAT3 and STAT5 with small molecules could interfere with a substantial proportion of human tumors^9–11^, and a number of potent small-molecule inhibitors of STAT3^12–16^ and STAT5^17–21^ have been published. We have previously presented compounds based on the chromone scaffold, which is found in flavones and isoflavones, as the first small molecule inhibitors of the protein-protein interaction domain [the Src homology 2 (SH2) domain] of STAT5^22,23^.

## Results and Discussion

**Figure 1:**
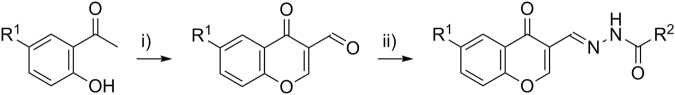
Synthesis of chromone derivatives targeting STAT SH2 domains. i) POCl_3_, DMF, 0 °C to RT, 1–2 h; ii) R^2^(CO)NHNH_2_, EtOH/H_2_O/HOAc, 4–5 h, reflux or EtOH/H_2_O/CHCl_3_, RT, 1–24 h.

The STAT family display a high degree of homology. In order to assess whether the scaffold of the most potent STAT5 inhibitor **1** (Table 1, entry 1)^22,23^ could be utilized for inhibition of other STAT family members, we explored the effect of substitutions at the 6-position of the chromone ring. Vilsmeyer-Haack formylation of 2-hydroxy acetophenones led to 3-formyl chromones, which were subsequently converted to the acyl hydrazones by reaction with hydrazides (Fig. 1). The target molecules were tested for their abilities to inhibit binding of fluorophore-labeled peptides to the SH2 domains of STAT1^24^, STAT3^25^, and STAT5b^26^ in assays based on fluorescence polarization (FP). The more distantly related SH2 domain of the tyrosine kinase Lck served as additional specificity control^27,28^.

**Table 1:** Activities of test compounds in fluorescence polarization (FP)-based competitive binding assays.

No	Structure	STAT1 app. IC_50_ [µM] or inhibition [%]	STAT3 app. IC_50_ [µM] or inhibition [%]	STAT5b app. IC_50_ [µM] or inhibition [%]
**1**		30 ± 1% inhibition at 80 µM	81 ± 4	23 ± 3
**2**		63 ± 6	74 ± 2	58 ± 1
**3**		32 ± 11	40 ± 3	31 ± 4% inhibition at 80 µM
**4**		36 ± 6% inhibition at 80 µM	25 ± 1	33 ± 7% inhibition at 80 µM
**5**		12 ± 3% inhibition at 80 µM	40 ± 2% inhibition at 80 µM	32 ± 24
**6**		58 ± 10	33 ± 6	26 ± 3
**7**		39 ± 2	22 ± 1	52 ± 12
**8**		24 ± 4	9.7 ± 1.8	32 ± 2% inhibition at 80 µM
**9**		36 ± 4% inhibition at 80 µM	45 ± 5% inhibition at 80 µM	no inhibition at 80 µM
**10**		36 ± 2% inhibition at 80 µM	35 ± 5% inhibition at 80 µM	no inhibition at 80 µM
**11**		19 ± 2% inhibition at 80 µM	65 ± 1	30 ± 7% inhibition at 80 µM
**12**		3 ± 3% inhibition at 80 µM	14 ± 2% inhibition at 80 µM	no inhibition at 80 µM
**8a**		15.0 ± 1.4	8.9 ± 0.8	27.7 ± 1.5
**9a**		10.7 ± 1.1	5.3 ± 0.4	34.8 ± 4.2

See the Supporting Information for details. Mean values ± standard deviations (s.d.) from three independent experiments are given.

Replacement of the hydrogen in the 6-position of the chromone ring of **1** with a halogen led to a systematic increase in activity against STAT3 with increasing halogen size, with a concomitant decrease in activity against STAT5 (Table 1). Apparent IC_50_ values of **1** and its fluoro (**2**)^22^, chloro (**3**), and bromo (**4**) derivatives against STAT3 decreased from 81 ± 4 µM (**1**) to 74 ± 2 µM (**2**), 40 ± 3 µM (**3**), and 25 ± 1 µM (**4**), respectively.

**Figure 2:**
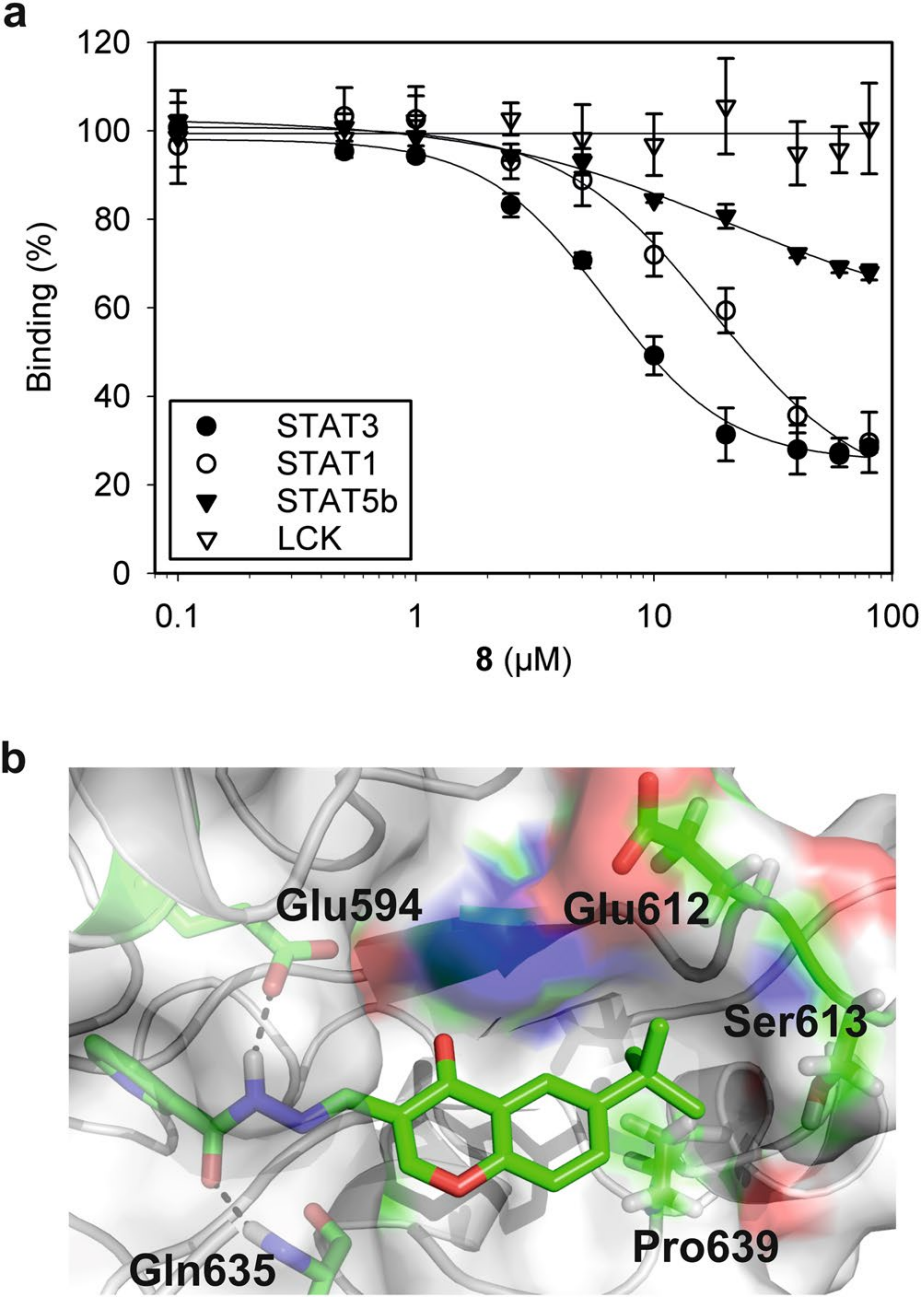
(**a**) Activity of **8** in FP-based assays. Error bars represent standard deviations from three independent experiments. (**b**) Docking pose of **8** in complex with STAT3. The Figure was created with PyMOL^51^.

Introduction of alkyl substituents larger than a methyl group (**5**) also had a distinct effect on the activity profiles of the compounds. Apparent IC_50_-values of the ethyl (**6**)^22^, isopropyl (**7**), and *tert*-butyl (**8**) derivatives decreased from 33 ± 6 µM (**6**) to 22 ± 1 µM (**7**) and 9.7 ± 1.8 µM (**8**) (Table 1, Fig. 2a). Gain in activity against STAT3 by introduction of alkyl groups was associated with a lesser gain in activity against STAT1 and a concomitant loss in activity against STAT5b (Table 1, Fig. 2a). None of the compounds showed significant activity against the more distantly related SH2 domain of the tyrosine kinase Lck^27^ (Supplementary Table S1).

Flexible docking of compounds **1**, **4**, **6**, **7**, and **8** to the STAT3 SH2 domain^29^ using RosettaLigand^30^ identified four clusters of highly populated binding poses for all five docked compounds (Supplementary Fig. S1). The binding poses in cluster 3 show a binding pocket for substituents at the 6-position of the chromone ring (Supplementary Fig. S1). This pocket, which is delineated by Pro639 and the hydrophobic components of the side chains of Glu612 and Ser613, is predicted to be occupied by the *tert*-butyl group of **8** (Fig. 2b). Derivatives **9** and **10** bearing a *tert*-pentyl and a phenyl group, respectively, displayed much weaker activity than **8**. These observations are consistent with a defined binding pocket for hydrophobic substituents at the 6-position, suitable for a *tert*-butyl group but not a larger substituent, and rule out non-specific hydrophobic effects as the underlying cause of the increased activities against STAT3. The predominantly hydrophobic nature of the binding pocket is in line with the lower activities of the methoxy-substituted compound **11** and the nitro group-bearing compound **12**. Docking of **8** into the SH2 domains of STAT1 and STAT5, starting from the pose of **8** as observed in STAT3, followed by gradient based minimization with ligand and receptor flexibility, revealed substantial clashes with the STAT5b SH2 domain, and lesser clashes with the STAT1 SH2 domain (Supplementary Fig. S2). This is consistent with the experimentally determined selectivity profile of **8** (Fig. 2a, Table 1).

The STAT3 binding pose indicates that the acyl hydrazone moiety of compound **8** lies in a narrow channel, which is not visible in the X-ray crystal structure of STAT3 bound to a phosphotyrosine-containing peptide segment (Supplementary Fig. S3)^29^. The predicted (*E*)-*trans*-configuration of the acyl hydrazone moiety in the protein-bound state was also observed in the X-ray structure of crystals of **8** grown from ethyl acetate / hexane (Supplementary Fig. S4). Substantial conformational changes of the protein are required for ligand binding (Supplementary Fig. S3), a process which can account for the observed time-dependence of inhibition by **8** (Supplementary Fig. S5). While substitution of the pyridyl moiety of **8** for phenyl (**13**) *para*-hydroxyphenyl (**14**), or *para*-methoxyphenyl (**15**) (Table 2) was accompanied by only a small loss in activity, a *meta*-methoxy substituent (**16**) markedly decreased activity, presumably for steric reasons. Substitution of the pyridyl ring for electron-rich, five-membered heteroaromatic rings is tolerated (**17**–**19**). In contrast, a *para*-fluoro (**20**) or a *para*-chloro substituent (**21**) on the phenyl ring strongly reduced binding. This behavior is consistent with hydrogen bonding between the molecules’ carbonyl group and the backbone amide of Gln635 as proposed by the docking studies (Fig. 2b), which is expected to suffer from electron-withdrawing substituents on the phenyl ring. Further evidence for hydrogen bonding arises from the inactivity of derivative **22**, in which the carbonyl group has been deleted. Substitution of the carbonyl by a sulfonyl group (compound **23**) is also not tolerated, indicating specific recognition of **8** by STAT3. Another hydrogen bond is suggested by the docking studies (Fig. 2b) between the side chain of STAT3 Glu594 and the acyl hydrazone moiety of **8**. The existence of this hydrogen bond is supported by the three-fold lower activity of the STAT3 Glu594Ala mutant (app. IC_50_ = 27 ± 1 µM, Supplementary Fig. S5) as compared to wild-type STAT3.

**Table 2:** Activities of test compounds in FP assays against the SH2 domains of STAT3 and STAT1.

No	Structure	STAT3 app. IC_50_ [µM] or inhibition [%]	STAT1 app. IC_50_ [µM] or inhibition [%]
**8**		9.7 ± 1.8	23 ± 6
**13**		14 ± 2	35 ± 4
**14**		19 ± 1	77 ± 16
**15**		26 ± 5	34 ± 4% inhibition at 80 µM
**16**		42 ± 2% at 80 µM	15 ± 2% at 80 µM
**17**		14 ± 1	49 ± 2
**18**		39 ± 4	32 ± 0.1% inhibition at 80 µM
**19**		50 ± 7	9 ± 4% inhibition at 80 µM
**20**		31 ± 8% at 80 µM	10 ± 5% inhibition at 80 µM
**21**		20 ± 2% at 80 µM	12 ± 4% inhibition at 80 µM
**22**		9 ± 3% at 80 µM	n. d.
**23**		4 ± 3% at 80 µM	n. d.

See the Supporting Information for details. n.d.: not determined. Mean values ± standard deviations (s.d.) from three independent experiments are given.

The formation of acyl hydrazones from aldehydes is a reversible reaction. In order to analyze whether formation of the aldehyde by hydrolysis of the acyl hydrazone may play a role in the observed inhibitory activities in FP, we tested the *tert*-butyl substituted aldehyde **8a**, which represents the synthetic precursor of the most active compound **8**, and the *tert*-pentyl substituted aldehyde **9a**, the precursor of the poorly active control compound **9**, against STAT3 (Table 1). Both aldehydes are active, but aldehyde **9a** displayed higher activity against STAT3 (app. IC_50_ = 5.3 ± 0.4 µM) than **8a** (app. IC_50_ = 8.9 ± 0.8 µM). The strongly divergent relative activities of the aldehydes **8a** and **9a** compared to the acyl hydrazones **8** and **9** imply that the activity of the acyl hydrazones is not a consequence of aldehyde formation. In addition, incubation of **8** in deuterated phosphate buffer (pD = 7.9, supplemented with DMSO-*d*_6_) for 24 h showed no sign of hydrolysis to the aldehyde **8a** in ^1^H-NMR studies (Supplementary Fig. S6).

**Figure 3:**
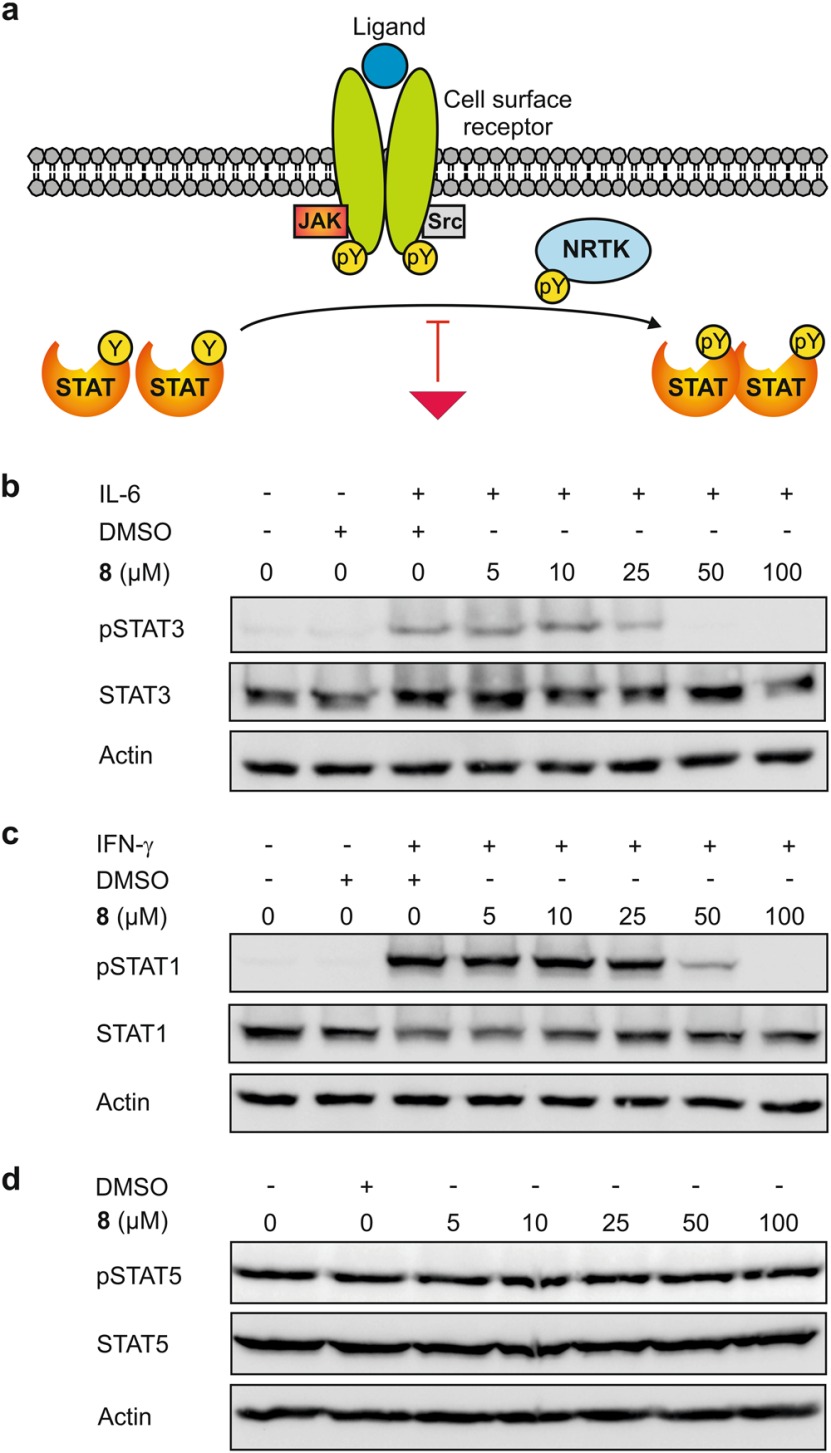
(**a**) Canonical STAT signaling pathway. An inhibitor of a STAT SH2 domain prevents phosphorylation of STATs at the conserved tyrosine residue C-terminal of the SH2 domain, thereby inhibiting STAT-mediated signal transduction. Effect of **8** on (**b**) IL-6-induced STAT3 phosphorylation in HepG2 cells, (**c**) IFN-γ-induced STAT1 phosphorylation in HepG2 cells, and (**d**) STAT5 phosphorylation in K562 cells. Cropped blots are displayed; full-length blots are presented in Supplementary Fig. S15.

In order to assess the effect of the most potent chromone **8** on STAT1, STAT3 and STAT5 activity in cells, we analyzed the phosphorylation state of a conserved tyrosine residue C-terminal of the STAT SH2 domain, which is dependent on the function of the SH2 domain. An inhibitor of a STAT SH2 domain prevents phosphorylation of STATs at this conserved tyrosine residue, thereby inhibiting STAT-mediated signal transduction (Fig. 3a). Consistent with its activity in the FP assay, **8** inhibited interleukin (IL)-6-stimulated STAT3 Tyr705 phosphorylation in HepG2 cells in a dose-dependent manner (Fig. 3b). Interferon (IFN)-γ-stimulated phosphorylation of STAT1 Tyr701 is inhibited to a lesser extent (Fig. 3c). In contrast, tyrosine phosphorylation of STAT5 in K562 cells is not inhibited (Fig. 3d). The activity profile in cells thus reflects the selectivity profile seen *in vitro* (Fig. 2a, Table 1), supporting the notion that cellular activities are mediated by functional inhibition of the STAT SH2 domains.

**Figure 4:**
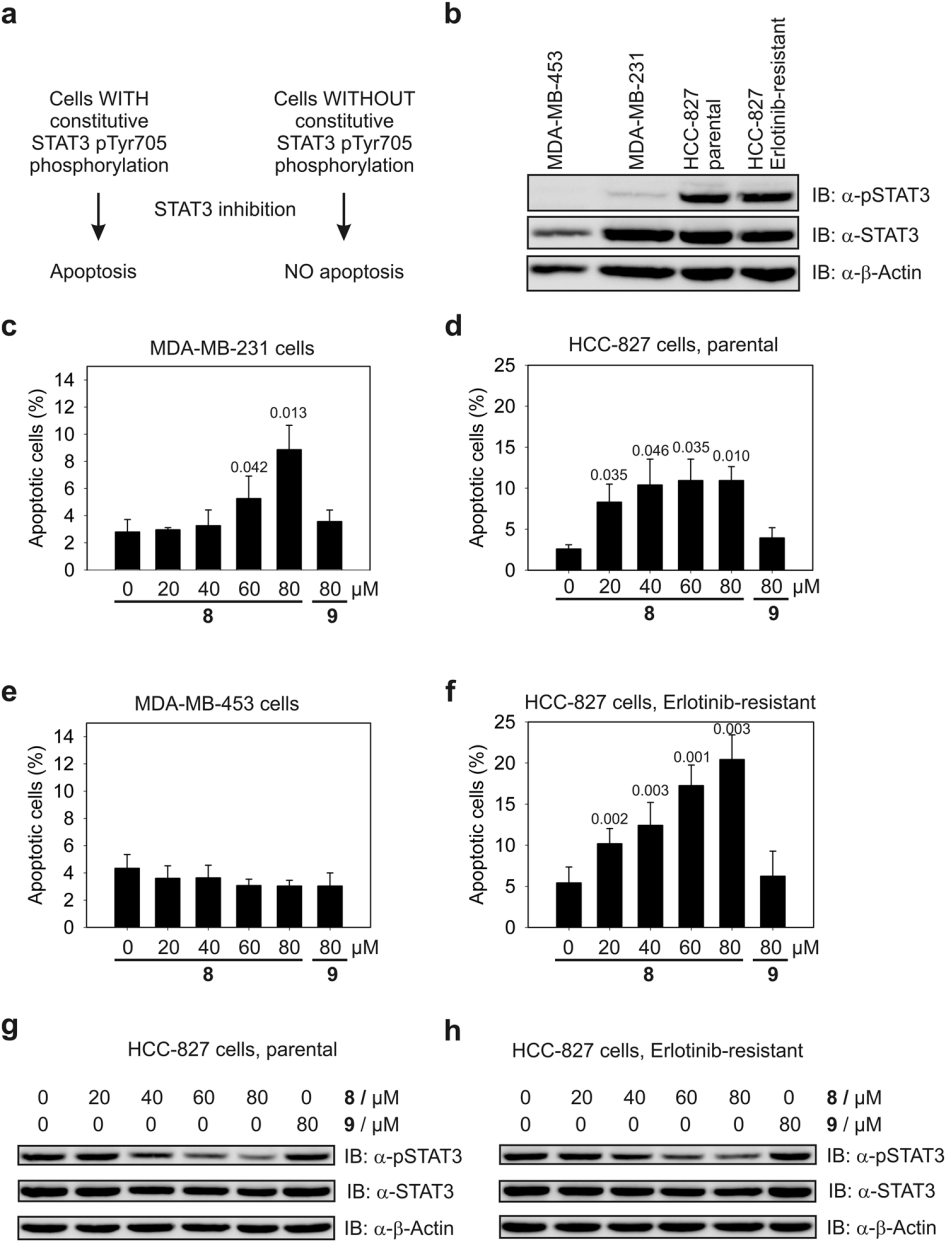
(**a**) Rationale for targeting STAT3. (**b**) Western Blot analysis of STAT3 Tyr705 phosphorylation. Cropped blots are displayed; full-length blots are presented in Supplementary Fig. S15. Effect of **8** and **9** on the increase in the apoptotic rate of (**c**) MDA-MB-231 cells (n = 3), (**d**) parental HCC-827 cells (n = 3), (**e**) MDA-MB-453 cells (n = 3), and (**f**) Erlotinib-resistant HCC-827 cells (n = 4) relative to control cells treated with DMSO only. Error bars represent standard deviations. Numbers on top of the bars indicate the p-values (t-test, two-tailed, paired). (**g**) Western Blot analysis of STAT3 Tyr705 phosphorylation in parental HCC-827 cells and (**h**) in Erlotinib-resistant HCC-827 cells. Cropped blots are displayed; full-length blots are presented in Supplementary Fig. S15.

STAT3 is constitutively activated in many human cancers^9^, including more than 50% of human breast and lung cancers^31^. Inhibition of STAT3 signaling in these cells increases the apoptotic rate, since tumor cells with constitutively activated STAT3 are dependent on STAT3 signaling for survival. In contrast, cells without constitutive STAT3 activation do not show an increase in their apoptotic rate in response to STAT3 inhibitors (Fig. 4a). **8** induced a dose-dependent, up to three-fold increase in the rate of apoptosis in MDA-MB-231 breast cancer cells, which have a moderate degree of constitutive STAT3 activation (Fig. 4b), compared to DMSO-treated control cells (Fig. 4c, Supplementary Fig. S7). HCC-827 non-small cell lung cancer (NSCLC) cells harbor stronger STAT3 activation than MDA-MB-231 cells (Fig. 4b) and also showed a more robust, up to four-fold increase in their apoptosis rate in response to **8** (Fig. 4d, Supplementary Fig. S8). In contrast, MDA-MB-453 breast cancer cells, which do not harbor constitutive STAT3 activation (Fig. 4b) did not show an increase in their apoptotic rate in response to **8** (Fig. 4e, Supplementary Fig. S9). The good correlation between the induction of apoptosis by **8** and the STAT3 Tyr705 phosphorylation status of the various cell lines used (Fig. 4b–e) is consistent with the notion that STAT3 inhibition by **8** is the underlying mechanism for the increased rate of apoptosis in MDA-MB-231 cells and HCC-827 cells. Since the tested cell lines do not display constitutive activation of STAT1, as indicated by the lack of constitutive phosphorylation of STAT1 Tyr701 (Supplementary Fig. S10), any effect of **8** on the STAT1 SH2 domain is unlikely to be relevant. Compound **9**, which was poorly active in FP assays (Table 1), did not significantly increase apoptosis in any of the cell lines (Fig. 4c–e, Supplementary Figs S7–S9). Since the aldehyde **9a** corresponding to the hydrazone **9** displays higher *in vitro*-activity against STAT3 than aldehyde **8a** (Table 1), this argues against the possibility that the cell-based effects of the acyl hydrazones might be caused by intracellular hydrolysis to the corresponding aldehydes. This conclusion is further supported by the results of the NMR stability assay, which showed no sign of aldehyde release from the acyl hydrazone **8** within 24 h, the time of exposure of the cell-based assays (Supplementary Fig. S6).

In the clinical setting, a subset of NSCLC patients respond well to small-molecule inhibitors of the EGF receptor, such as Erlotinib (Tarceva, Roche)^32^. Unfortunately, therapy resistance is a frequent occurrence despite initial treatment success, leading to disease relapse^33,34^. Given the ability of **8** to induce apoptosis in HCC-827 cells, we asked whether it could also induce apoptosis in HCC-827-derived Erlotinib-resistant cells. Parental HCC-827 cells are highly sensitive to Erlotinib (EC_50_ = 0.015 ± 0.005 µM, Supplementary Fig. S11)^35^. By treating HCC-827 cells with increasing concentrations of Erlotinib over an extended period of time, we produced HCC-827-derived cells which had acquired over 400-fold reduced sensitivity to Erlotinib (EC_50_ = 6.4 ± 2.2 µM, Supplementary Fig. S11). While parental HCC-827 cells showed a significant increase in apoptosis in the presence of as little as 1 nM Erlotinib (p = 0.027, Supplementary Figs S11, S12) and a more than tenfold induction of apoptosis in the presence of 1 µM Erlotinib (Supplementary Fig. S11), HCC-827-derived cells with acquired resistance did not show increased apoptosis at 10 nM Erlotinib, and only a twofold increase of the apoptotic rate at 1 µM Erlotinib (Supplementary Figs S11, S13). In contrast, **8** was equally effective at increasing the apoptotic rate of Erlotinib-resistant HCC-827 cells and parental HCC-827 cells compared to the respective DMSO-treated control cells (Fig. 4d,f, Supplementary Figs S8, S14).

Western Blot analysis revealed a dose-dependent inhibitory effect of compound **8** on STAT3 Tyr705 phosphorylation in both parental HCC-827 and Erlotinib-resistant HCC-827 cells (Fig. 4g,h). In contrast, the control compound **9** did not increase the apoptotic rate of either HCC-827 cell line (Fig. 4d,f), and also did not reduce STAT3 Tyr705 phosphorylation (Fig. 4g,h), indicating that the induction of apoptosis by **8** in both parental HCC-827 and Erlotinib-resistant HCC-827 cells is caused by inhibition of STAT3. This notion is supported by the strong correlation between the STAT3 Tyr705 phosphorylation status of the tested cell lines (Fig. 4b) and the increase in rate of apoptosis caused by **8** (Fig. 4c–f). Whilst inhibition of the JAK2/STAT3 signaling pathway has been reported as a means by which to inhibit tumor cells with a poor response to EGFR inhibitors^36–47^, our data demonstrate that NSCLC cells with acquired resistance against Erlotinib can be targeted by direct inhibition of STAT3.

In summary, we have demonstrated that the introduction of suitable hydrophobic substituents at the 6-position of the chromone-based STAT5 inhibitor **1** results in activity against STAT3 and, to a lesser extent, STAT1, with a concomitant decrease in activity against STAT5. The binding mode of the most potent STAT3 inhibitor **8** was analyzed by the investigation of structure-activity relationships, which were facilitated by chemical synthesis and biochemical activity analysis, as well as molecular docking and point mutant analysis. **8** represents the first chromone-based acylhydrazone shown to target STAT3 with selectivity over STAT5 and STAT1 in cell-based assays, and increases the apoptotic rate of cultured NSCLC cells in a STAT3-dependent manner. This ability of **8** also extends to HCC-827 cells which have acquired resistance against Erlotinib, a clinically used inhibitor of the EGF receptor. These data indicate that direct inhibition of STAT3 is a powerful approach by which to target STAT3-dependent NSCLC cells that have acquired resistance against Erlotinib. Consequently, **8** was dubbed Erasin (**E**rlotinib-**r**esistance **a**ntagonizing **S**TAT3 **in**hibitor).

The chromone moiety contained in Erasin (**8**) is found in the natural product classes of flavones and isoflavones. Natural products are generally viewed as excellent starting points for inhibitor development^6^. However, this important source of chemical inspiration can currently only be poorly exploited for the design of inhibitors of protein-protein interactions owing to the sparsity of known natural product-based lead structures. The development of the STAT3 inhibitor Erasin (**8**) from the STAT5 inhibitor **1** validates chromone-based acyl hydrazones as privileged structures for the inhibition of protein-protein interactions mediated by STAT SH2 domains. Chromone-based acyl hydrazones thus represent a rare case of natural product-based structures that can be fine-tuned to inhibit members of protein-protein interaction domains by simple variation of the substitution pattern^18,48^.

## Methods

Synthesis and spectroscopic characterization of synthesized compounds can be found in the Supplementary Information.

### Fluorescence polarization assays

Assays were performed essentially as described^22^. In brief, protein was incubated with the test compounds for 1 h at room temperature at the following protein concentrations: STAT1: 400 nM, STAT3: 210 nM, STAT5b: 300 nM, Lck SH2: 35 nM. Subsequently, the corresponding fluorophore-labeled peptide was added: STAT1: (5-carboxyfluorescein)-GpYDKPHVL-OH, derived from the interferon-γ receptor^24^; STAT3: (5-carboxyfluorescein)-GpYLPQTV-NH_2_, derived from the gp130 subunit of the IL-6 receptor^25^; STAT5b: (5-carboxyfluorescein)-GpYLVLDKW-OH, derived from the erythropoietin receptor^26^; Lck SH2: (5-carboxyfluorescein)-GpYEEIP-OH, derived from the middle-T antigen^28^. After a further hour of incubation, fluorescence polarization was measured in a plate reader (Tecan Infinite F500). Apparent IC_50_ data shown in Tables 1, 2, and S1 and the inhibition curves shown in Fig. 2a correspond to this time point. The majority of the pipetting work was carried out using a Biomek FXp workstation (Beckman-Coulter). Buffer composition: 10 mM Tris pH 8.0, 50 mM NaCl, 1 mM EDTA, 1 mM dithiothreitol (DTT), 0.1% Nonidet P-40, and 2% DMSO.

### Selection of protein models

Protein structures for computational docking studies were taken from crystal structures stored in the Protein Data Bank: the 3.0 Å crystal structure of unphosphorylated STAT1 complexed with a phosphopeptide (1YVL)^49^ and the 2.25 Å structure of the STAT3 homodimer bound to its DNA recognition site (1BG1)^29^. To simplify docking, the proteins were truncated to include only the SH2 domain containing the ligand binding site. The protein structures then underwent eight rounds of energy minimization in Rosetta, creating 25 low-energy models that approximate the native state and ensure that residue side chains are packed correctly. At the time of writing, there was no crystal structure available depicting the binding of any STAT protein to the peptide used in the fluorescence polarization assay (pYLPQTV), so the crystal structure of the binding interaction between STAT3 and the peptide in PDB ID 1BG1 (pYLKTKF)^29^ was used to approximate peptide binding interactions in the following experiments.

### Docking of ligands into protein models

In preparation for docking, ligand conformers were generated by MOE (Molecular Operating Environment, Chemical Computing Group, Ontario, Canada) using the MMFF94x and Born solvation model. Conformers were generated using 10,000 iterations of the Low Mode MD method with a redundancy cutoff of 0.25 Å and an iteration rejection limit of 100. Prior to docking, each ligand was manually placed into the approximate binding site as indicated in the crystal structure of phosphorylated, dimeric STAT3 (PDB: 1BG1)^29^. During the low-resolution docking phase, each ligand was allowed to sample binding modes in a 5.0 Å radius from the center of the approximate binding site. During this phase, rigid body translation of the centroid of the ligand was performed until the position of the geometric center did not conflict with positions occupied by the atoms in the protein. Once this was satisfied, 1000 cycles of full rotational exploration were performed until Rosetta energy calculations fell below a threshold value. For limited docking of STAT1 and STAT5, ligands were aligned to starting position by pair fitting and the low resolution step were skipped. Next, high resolution docking carried out six cycles of side-chain rotamer and ligand conformer sampling coupled with 0.1 Å, 0.005 radian ligand movements simultaneously in a Monte Carlo simulated annealing algorithm. Rotatable bonds within the ligand, those not participating in planar conjugated bonds, were allowed full flexibility as indicated within the ligand parameters file generated by MOE. A final minimization combined side-chain rotamer sampling with backbone torsion angle minimization with harmonic constraints on the C-alpha atoms. The energy function used to score the docking models contains parameters for van der Waals attractive and repulsive forces, hydrogen bonding, electrostatic interactions between amino acids, statistical energy derived from side-chain conformation probability, and solvation assessments of both side-chain/side-chain and side-chain/ligand interactions. For each protein-ligand combination, 2500 docked complexes were produced.

### Analysis of binding modes

Following ligand docking, the top 1 percent of binding modes were taken for analysis. To enable quantitative comparison of ligand binding orientations, the root mean square deviation (RMSD) was computed over the ligands in their binding modes. Pairwise RMSD was then computed over all top-scoring models. The poses were then clustered using bcl::cluster with an RMSD cutoff of 3 Å. The largest clusters were analyzed qualitatively and quantitatively to determine possible binding interactions.

### NMR-based stability analysis of compound 8

Na_2_DPO_4_ (C/D/N Isotopes, 8.5 mM) and NaD_2_PO_4_ (C/D/N Isotopes, 3.3 mM) were dissolved in D_2_O to give a total phosphate concentration of 11.8 mM. The measured pH of 7.5 corresponds to a pD of 7.91 according to the literature^50^.

### Analysis of STAT phosphorylation by Western Blot

HepG2 cells were plated in 6-well plates (Corning #3516) and allowed to reach approximately 60% confluence. The medium was then replaced with serum-free medium for 24 h. Cells were subsequently pre-treated with compound or DMSO (final DMSO concentration: 0.2%) for 1 h at 37 °C, prior to stimulation for 15 min with 50 ng/mL IFNγ (Pepro Tech, for STAT1 phosphorylation), or 30 min with 50 ng/mL IL-6 (Pepro Tech, for STAT3 phosphorylation). Cells were treated with test compound or DMSO for 1 h (final DMSO concentration 0.2%). K562 cells (1.5 × 10^6^ cells per well) were seeded in 6-well plates (Corning #3516) and were treated with test compound or DMSO for 1.5 h (final DMSO concentration 0.2%). HCC-827 parental or HCC-827 cells with acquired Erlotinib resistance (both 1 × 10^6^ cells per well) were seeded in 6-well tissue culture plates (Corning #3516) and were allowed to adhere overnight. Afterwards, cells were treated with the test compounds or DMSO (final DMSO concentration: 0.2%) for 24 h. Afterwards, the cells were washed twice with cold phosphate buffered saline (PBS).

### Cell lysates

Whole-cell lysates were prepared with lysis buffer containing 50 mM Tris/HCl pH 7.5, 150 mM NaCl, 10 mM Na_4_P_2_O_7_, 10% glycerol, 1% Triton X-100 and 1 mM EDTA, with phosphatase/protease inhibitors 10 mM NaF, 1 mM Na_3_VO_4_, 1 mM PMSF and 100 ng/ml aprotinin added freshly prior to use. For lysates from HCC-827 cells, both the adherent cells and the cell components from the cell culture supernatant were combined and treated with lysis buffer to mimic the conditions of the apoptosis assay. Lysates were snap frozen in liquid nitrogen and stored at −80 °C.

### Western Blotting

Lysates were separated on a polyacrylamide gel under denaturing conditions, and transferred to a nitrocellulose membrane. STAT phosphorylation was assessed using rabbit monoclonal antibodies against the phosphorylated forms only of STAT1, STAT3 or STAT5 (Cell Signaling, 1:1000) with secondary antibody swine anti-rabbit HRP from Dako, 1:3000), followed by reblotting with rabbit monoclonal antibodies against total STAT1, STAT3 or STAT5 (Cell Signaling), and subsequently β-actin loading control. Visualization was carried out using an ImageQuant digital imaging system (GE Healthcare).

### Cell viability assay

Parental HCC-827 cells or HCC-827 cells with acquired Erlotinib resistance were seeded at a density of 4 × 10^3^ cells per well in a 96-well tissue culture plate (Corning #3596) and were treated with Erlotinib at the indicated concentrations (final DMSO concentration: 0.2%) for 92 h. Subsequently, 10 µl WST-1 solution (Roche, 1:10 dilution with cell culture medium) was added to each well, and incubated for 4 h (96 h total Erlotinib treatment). The absorbance at 440 nm was analyzed, using the absorbance at 650 nm as a reference. Experiments were carried out in triplicate.

### Apoptosis assay

MDA-MB-453 cells (3 × 10^5^ cells per well), MDA-MB-231 cells (2 × 10^5^ cells per well), parental HCC-827 or Erlotinib-resistant HCC-827 cells (both 2.5 × 10^5^ cells per well) were seeded in 24-well tissue culture plates (Corning #3524) and treated with test compound or DMSO (final DMSO concentration: 0.2%) for 24 h. Afterwards, the cell culture supernatant from each well was collected. Cells were washed twice with warm PBS and incubated with Accutase (BD Bioscience #561527) at 37 °C for 10 min. The supernatants were then returned to each well to neutralize the Accutase solution and the cells were centrifuged at 3000 rpm, at 4 °C for 5 min. The cell pellets were washed twice with cold PBS and centrifuged again. PE Annexin V Apoptosis Detection Kit I (BD Bioscience, 559763) was used for cell staining. Cells were resuspended in 1 x binding buffer and incubated with PE Annexin V and 7-AAD at 4 °C for 30 min. Apoptosis was measured using an LSR II flow cytometer (BD Bioscience). Experiments were carried out in at least triplicate.

### Data availability

The datasets generated during and/or analysed during the current study are available from the corresponding author on reasonable request.

## Electronic supplementary material

Supplementary Information
