# Supplementary material for: Development of Erasin: a chromone-based STAT3 inhibitor which induces apoptosis in Erlotinib-resistant lung cancer cells
Source: Sci Rep. 2017 Dec 12;7:17390. doi: 10.1038/s41598-017-17600-x (PMC5727211; doi:10.1038/s41598-017-17600-x)

Supplementary Information

Development of Erasin: a chromone-based STAT3 inhibitor which induces apoptosis in Erlotinib-resistant lung cancer cells

Christian Lis[#,1], Stefan Rubner[#,1], Martin Roatsch[#,1], Angela Berg[1], Tyler Gilcrest[2], Darwin Fu[2], Elizabeth Nguyen[2], Anne-Marie Schmidt[1], Harald Krautscheid[3], Jens Meiler[2], and Thorsten Berg*[,1]

Table of contents

Supplementary Figures and Table ... 2
Supplementary Table S1 ... 2
Supplementary Figure S1 ... 3
Supplementary Figure S2 ... 4
Supplementary Figure S3 ... 5
Supplementary Figure S4 ... 6
Supplementary Figure S5 ... 7
Supplementary Figure S6 ... 7
Supplementary Figure S7 ... 8
Supplementary Figure S8 ... 8
Supplementary Figure S9 ... 9
Supplementary Figure S10 ... 9
Supplementary Figure S11 ... 10
Supplementary Figure S12 ... 11
Supplementary Figure S13 ... 11
Supplementary Figure S14 ... 12
Supplementary Figure S15 ... 13
Synthesis and spectroscopic characterization of synthesized compounds ... 16
Synthesis and spectroscopic characterization of precursor molecules ... 16
Synthesis and spectroscopic characterization of Chromone-Based Acyl Hydrazones ... 18
Supplementary Methods ... 27
Supplementary references ... 28
NMR spectra ... 29

Supplementary Figures and Table

Supplementary Table S1

No	Structure	Lck app. IC_{50} [µM] or inhibition [%]
**1**	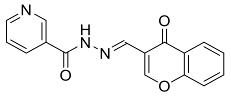	no inhibition at 80 µM
**2**	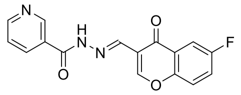	no inhibition at 80 µM
**3**	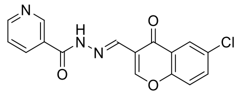	6 ± 6% inhibition at 80 µM
**4**	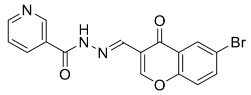	13 ± 2% inhibition at 80 µM
**5**	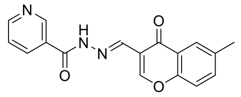	no inhibition at 80 µM
**6**	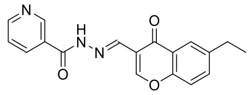	no inhibition at 80 µM
**7**	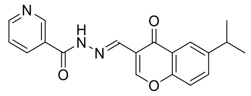	no inhibition at 80 µM
**8**	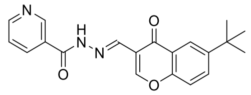	no inhibition at 80 µM
**9**	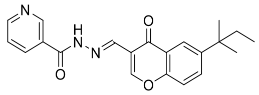	no inhibition at 80 µM
**10**	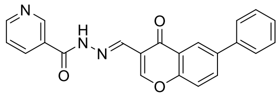	no inhibition at 80 µM
**11**	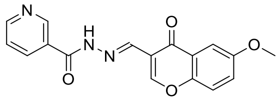	23 ± 2% inhibition at 80 µM
**12**	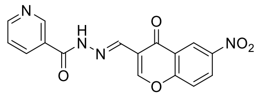	22 ± 6% inhibition at 80 µM

Supplementary Table S1: Activities of test compounds in fluorescence polarization assays against the SH2 domains of Lck. See the Supporting Methods for details.

Supplementary Figure S1

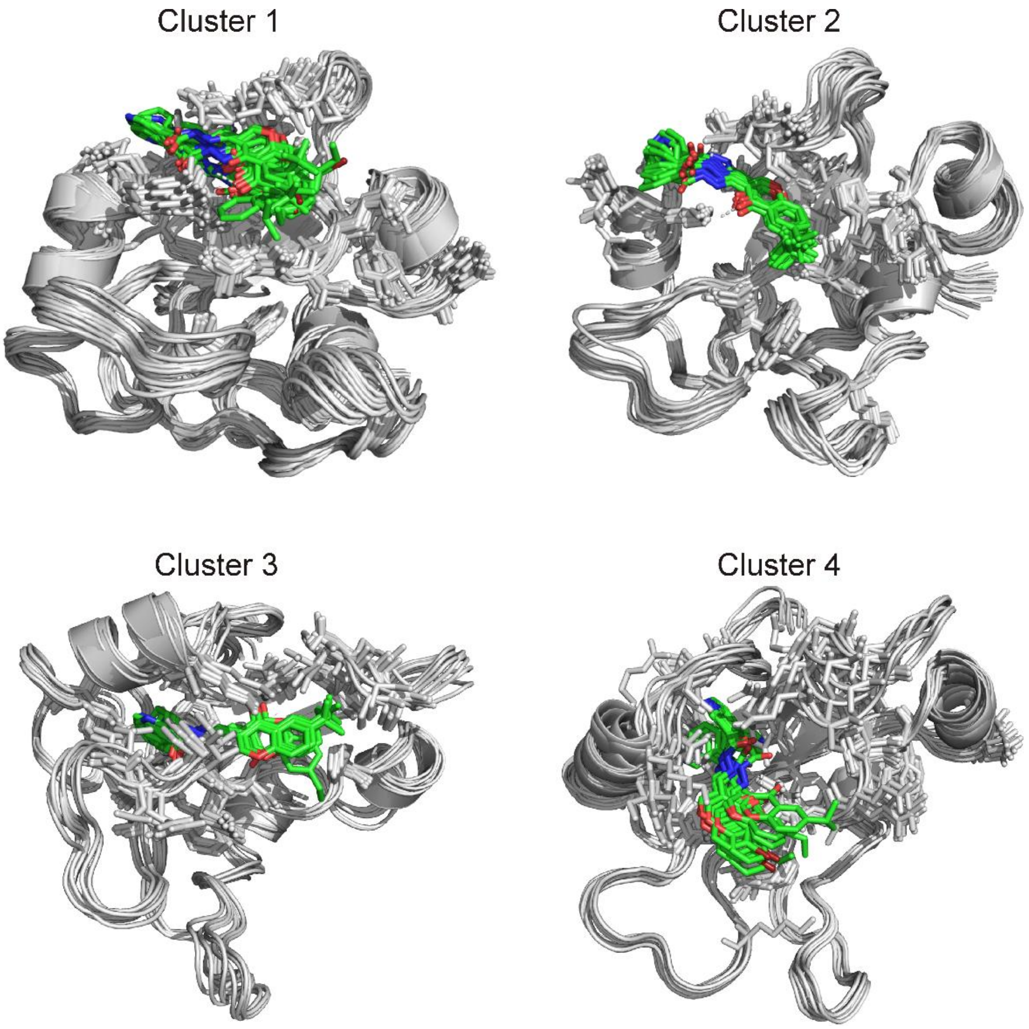

Supplementary Figure S1: Rosetta-based docking of compounds **1**, **4**, **6**, **7**, and **8** against the STAT3 SH2 domain proposes 4 clusters of docking poses.

Supplementary Figure S2

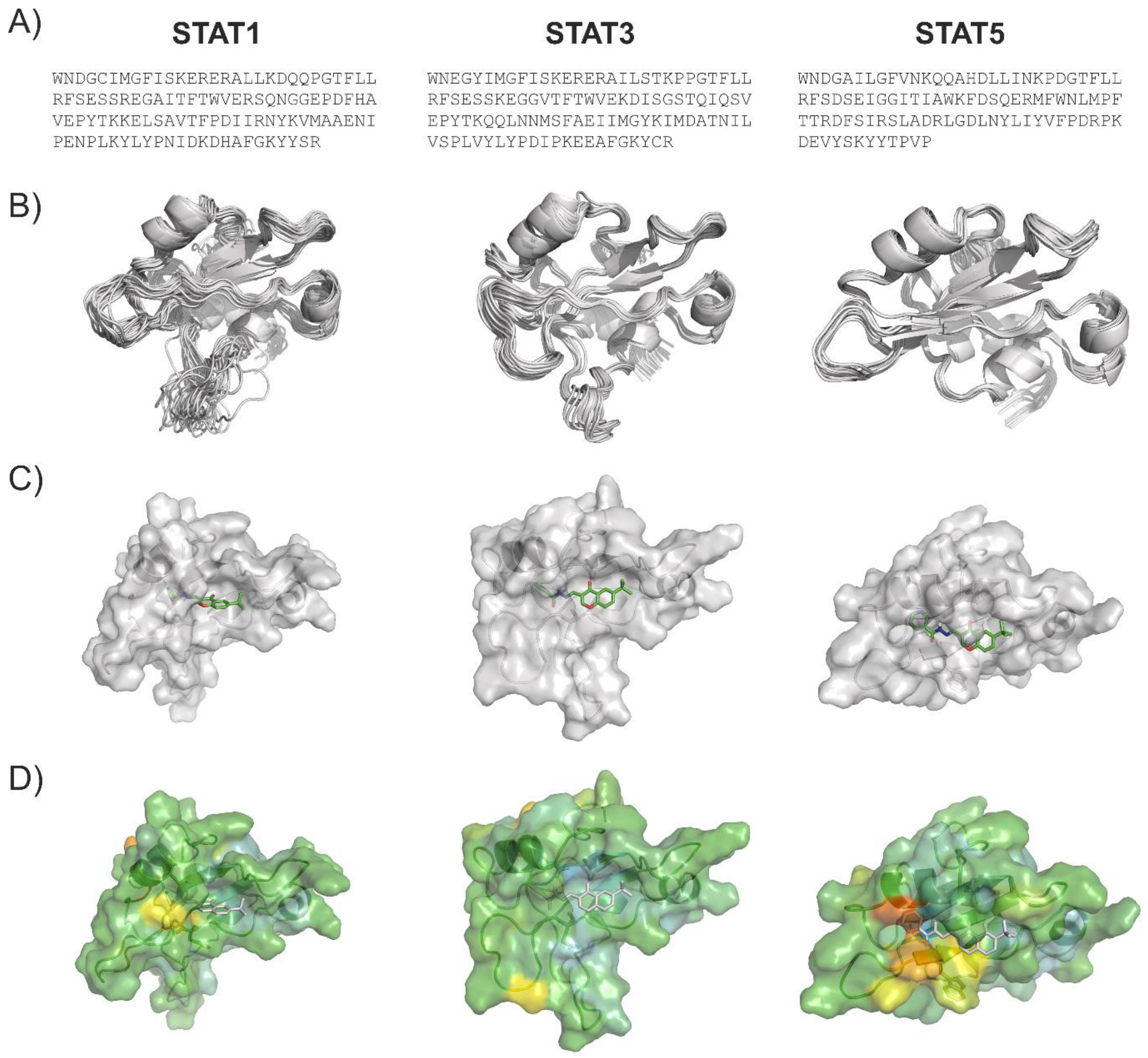

Supplementary Figure S2: A) Amino acid sequence of the SH2 domains of STAT1, STAT3, and STAT5b. B) Structural models of the SH2 domains of STAT1, STAT3, and STAT5b in the absence of a ligand show regions of protein flexibility. C) Representative model of a cluster 3 binding pose of **8** bound to the STAT3 SH2 domain, and similar poses for STAT1 and STAT5b obtained by docking starting from the pose of **8** as observed in STAT3. D) Surface representation for models in panel C showing per-residue Rosetta energy units using rainbow color codes (red-orange-yellow-green-blue), with the least favorable interactions shown in red and the most favorable interactions shown in blue. Clashes (indicated in orange and yellow) do not occur in the binding pocket of **8** in STAT3, but to a small extent, in the equivalent region of STAT1, and to a larger extent, in STAT5b. These clashes are primarily due to intermolecular repulsive interactions scored on a Lennard-Jones potential and unfavorable protein dihedral angles scored by a knowledge-based potential.

Supplementary Figure S3

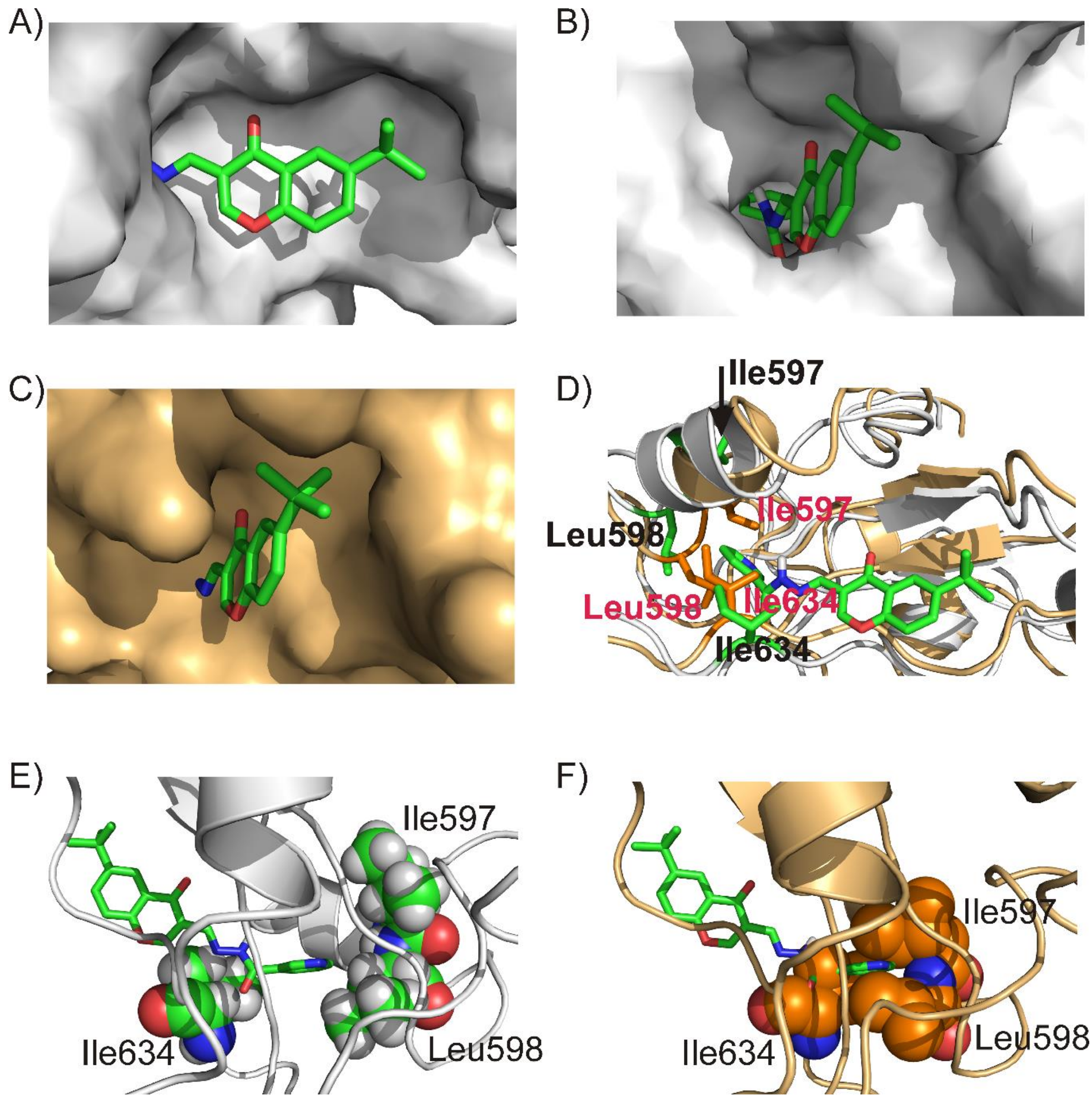

Supplementary Figure S3. A) Docking pose of **8** in the STAT3 SH2 domain, frontal view. B) View of the docking pose of **8** in the STAT3 SH2 domain along the tight channel accommodating the acyl hydrazone moiety of **8**. C) Overlay of the docking pose of **8** with the crystal structure of phosphorylated STAT3 (PDB 1BG1).[1] D) Overlay of the docking pose of **8** in STAT3 (STAT3 backbone in light grey, highlighted amino acid side chains in green, amino acid numbering in black) and the crystal structure of phosphorylated STAT3 (protein backbone shown in ochre, highlighted amino acid side chains shown in orange, amino acid numbering in red). E) View of the docking pose of **8** with STAT3 Ile597, Leu598, Ile634 displayed as spheres; F) The overlay of the docking pose of **8** and the STAT3 X-ray structure (PDB 1BG1),[1] with Ile597, Leu598, Ile634 displayed as spheres, indicates the need for significant conformational changes to accommodate **8**.

Supplementary Figure S4

A)

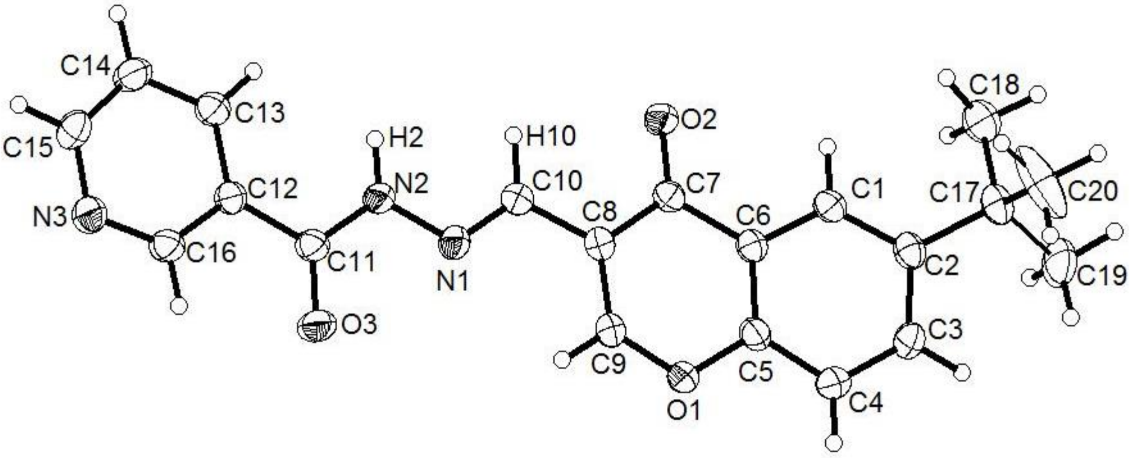

B)

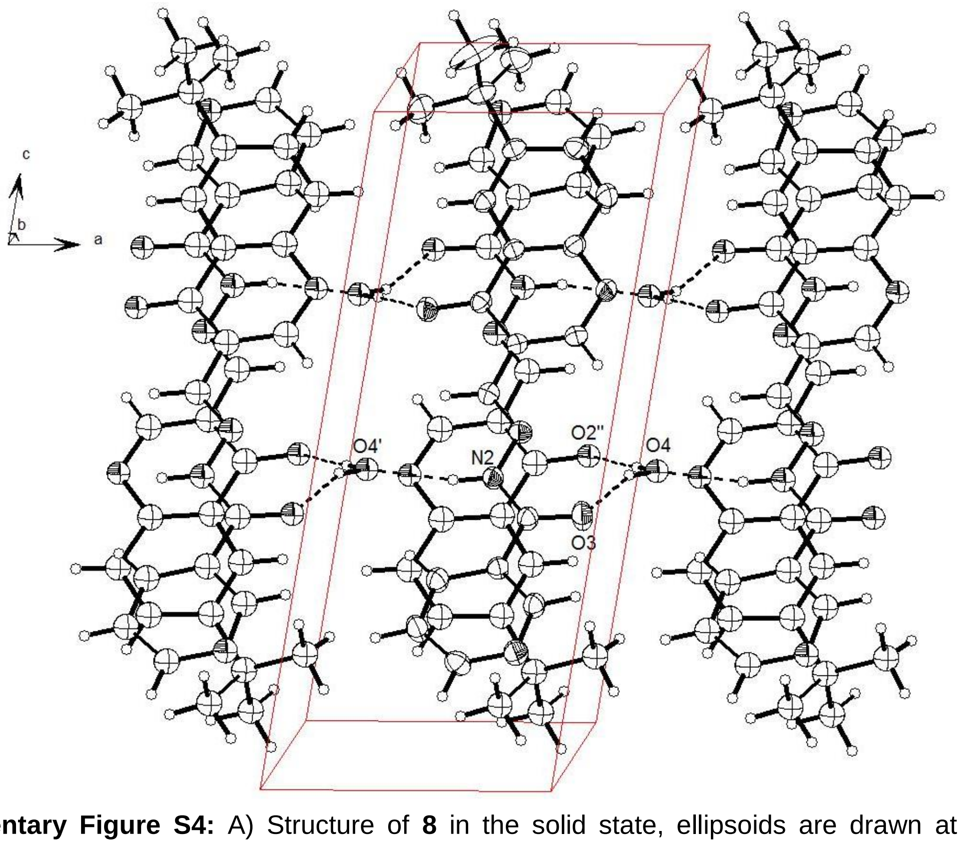

Supplementary Figure S4: A) Structure of **8** in the solid state, ellipsoids are drawn at the 50 % probability level. B) Packing diagram for **8**, hydrogen bonds are shown as dashed lines. Selected bond lengths and angles: C5–O1 1.3734(14), C6–C7 1.4738(15), C7–C8 1.4577(16), C7–O2 1.2306(14), C8–C9 1.3489(17), C8–C10 1.4685(16), C9–O1 1.3418(14), C10–N1 1.2771(16), C11–N2 1.3564(15), N1–N2 1.3754(14) Å; N1–C10–C8 118.58(11), O3–C11–N2 122.84(11), O3–C11–C12 120.07(10), N2–C11–C12 117.09(10), C10–N1–N2 117.16(10), C11–N2–N1 116.52(10), C5–O1–C5 118.29(10) °.

The co-crystallizing water molecule (O4, H3o, H4o) is involved in hydrogen bonding:

D–H	d(D–H)	d(H..A)	<DHA	d(D..A)	A
N2–H2	0.895	1.970	167.66	2.850	O4 [x–1, y, z]
O4–H3o	0.855	1.978	176.37	2.831	O2 [–x+1, –y+2, –z+1]
O4–H4o	0.871	1.989	158.49	2.818	O3

Supplementary Figure S5

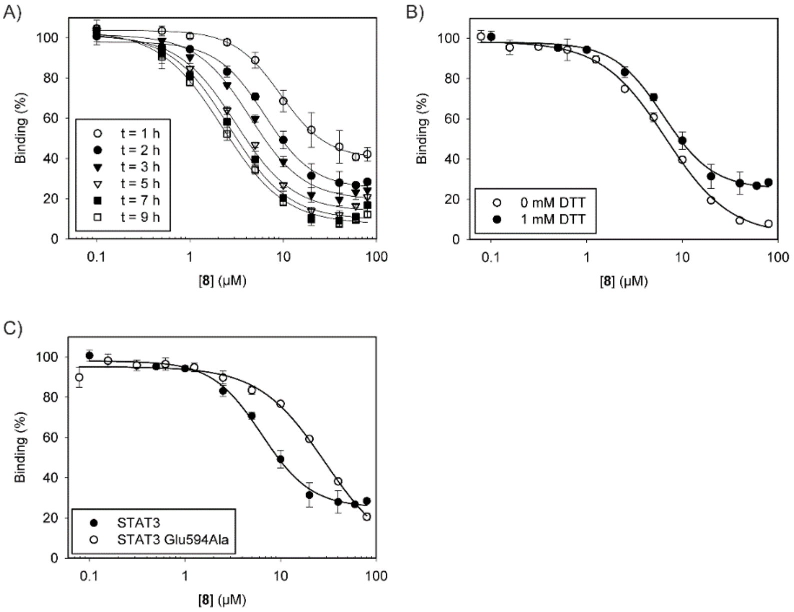

Supplementary Figure S5: A) The apparent IC_{50} values of **8** against wild-type STAT3 decrease with time, with an apparent IC_{50} of only 2.8 µM after 9 h. This reduction tails off, consistent with the idea that inhibition of STAT3 by **8** is a slow but reversible process. An alternative explanation for time-dependent inhibition would be covalent modification of the Michael acceptor of the chromones by the protein. While we cannot formally exclude this possibility, two observations argue against cysteine alkylation as the dominant mode of action. Firstly, the experimentally validated docking pose (shown in Figure 2B and Supplementary Figure S3) does not reveal any nucleophilic amino acids in the vicinity of the activated double bond of **8**. B) Secondly, omission of the strong nucleophile dithiothreitol (DTT) from the assay buffer does not significantly increase the inhibitory activity of **8** against STAT3, indicating that **8** is not easily susceptible to covalent modification by nucleophiles. Data after 2 h of incubation are shown. C) Activity of **8** in a fluorescence polarization assay against wild-type STAT3 or a STAT3 Glu594Ala mutant after 2 h incubation in the presence of 1 mM DTT.

Supplementary Figure S6

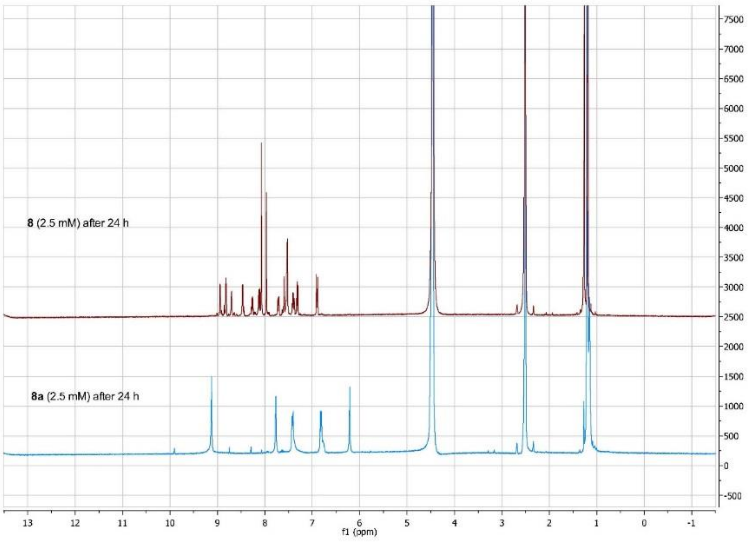

Supplementary Figure S6: ^{1}H NMR of acyl hydrazone **8** (2.5 mM, top) and the corresponding aldehyde **8a** (2.5 mM, bottom) after 24 h incubation in 50% 11.8 mM deuterated phosphate buffer (pD = 7.9) and 50% DMSO-d_6. The addition of DMSO-d_6 was required to achieve sufficient solubility under the conditions of the NMR studies.

Supplementary Figure S7

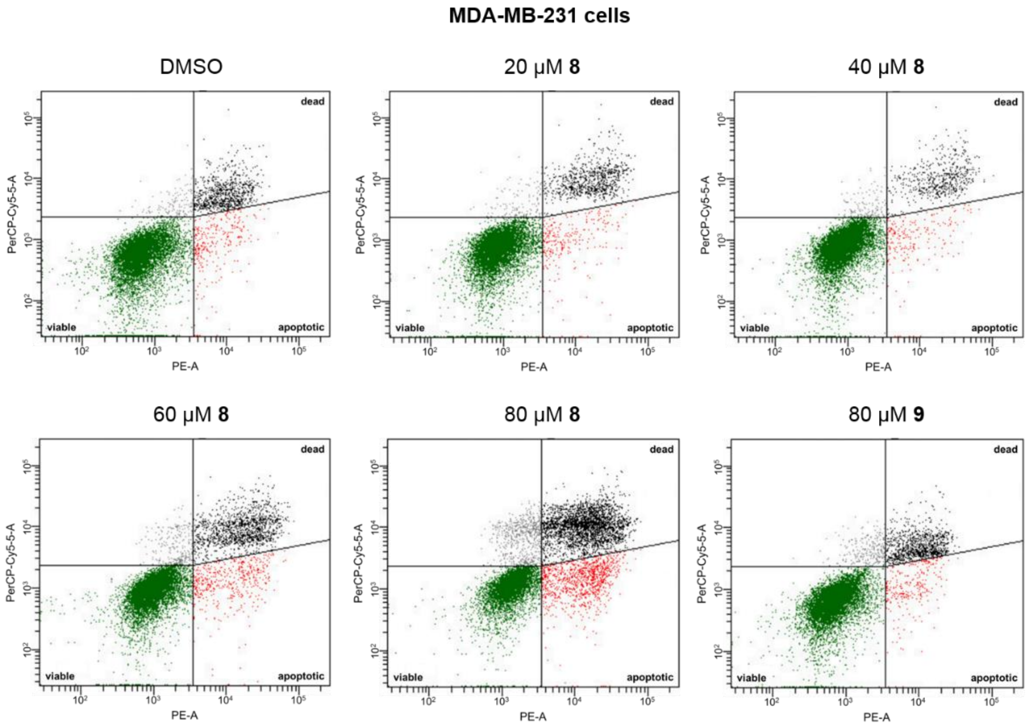

Supplementary Figure S7: Representative results of flow cytometry analysis of MDA-MB-231 cells treated with the indicated concentrations of **8** and **9** for 24 h. Apoptotic cells, as characterized by Annexin V staining and the absence of 7-AAD staining, are depicted in the lower right-hand quadrant.

Supplementary Figure S8

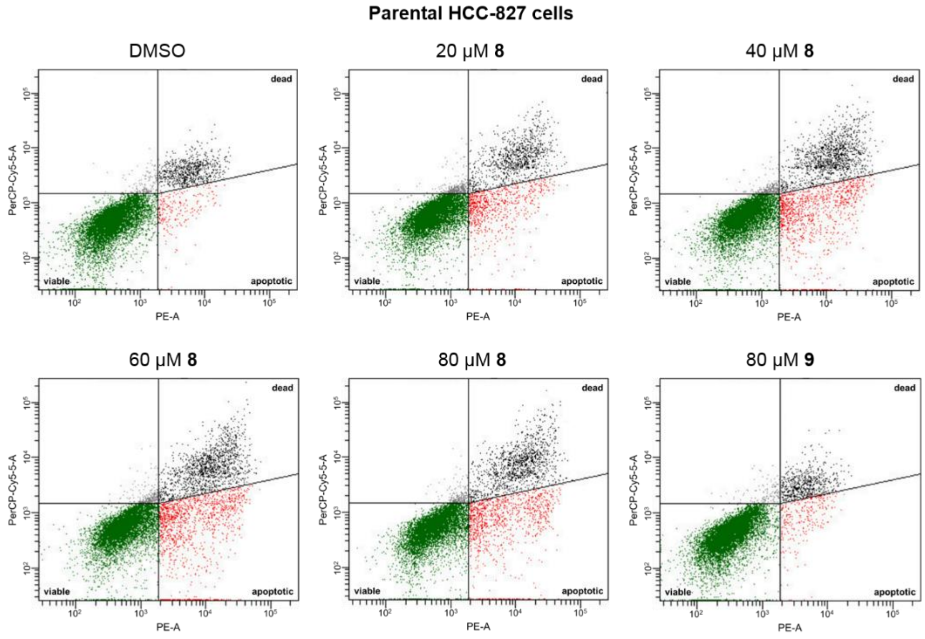

Supplementary Figure S8: Representative results of flow cytometry analysis of HCC-827 cells treated with the indicated concentrations of **8** and **9** for 24 h. Apoptotic cells, as characterized by Annexin V staining and the absence of 7-AAD staining, are depicted in the lower right-hand quadrant.

Supplementary Figure S9

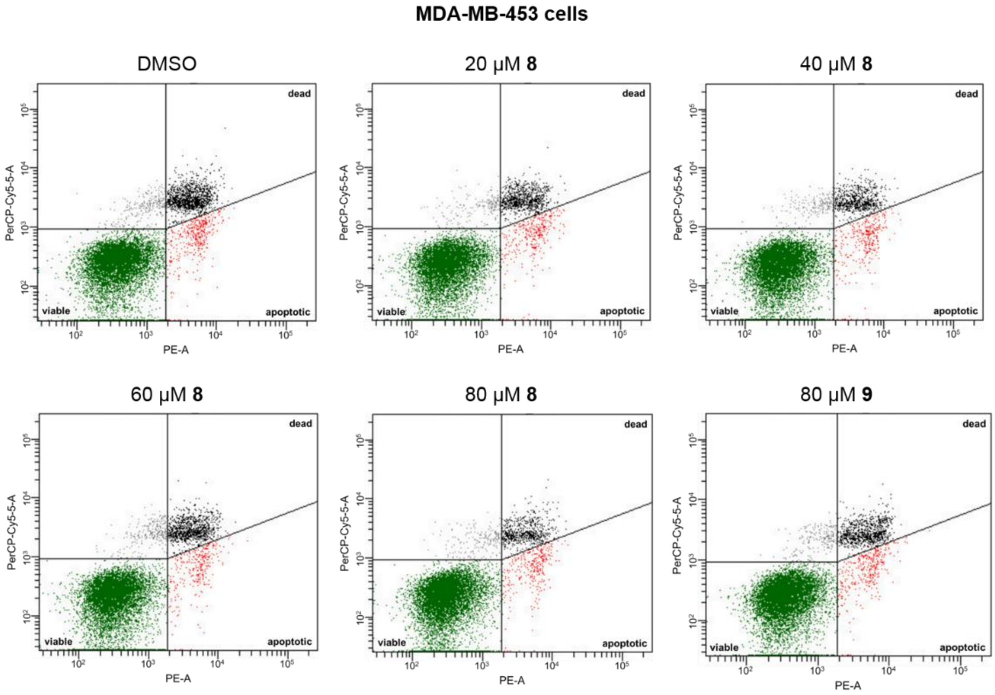

Supplementary Figure S9: Representative results of flow cytometry analysis of MDA-MB-453 cells treated with the indicated concentrations of **8** and **9** for 24 h. Apoptotic cells, as characterized by Annexin V staining and the absence of 7-AAD staining, are depicted in the lower right-hand quadrant.

Supplementary Figure S10

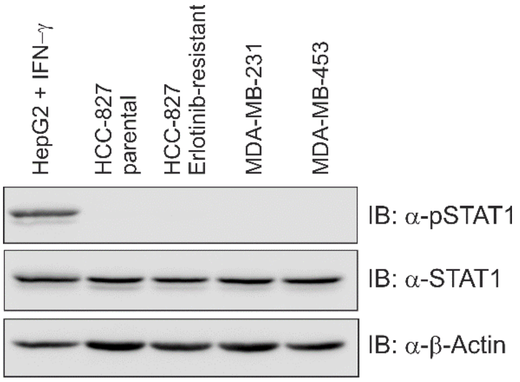

Supplementary Figure S10: Western Blot analysis of parental HCC-827, Erlotinib-resistant HCC-827, MDA-MB-453, and MDA-MB-231 cells against STAT1 Tyr701 phosphorylation. Lysates from HepG2 cells stimulated with IFN-γ (left lane) serve as positive control. Cropped blots are displayed; full-length blots are presented in Supplementary Fig. S15.

Supplementary Figure S11

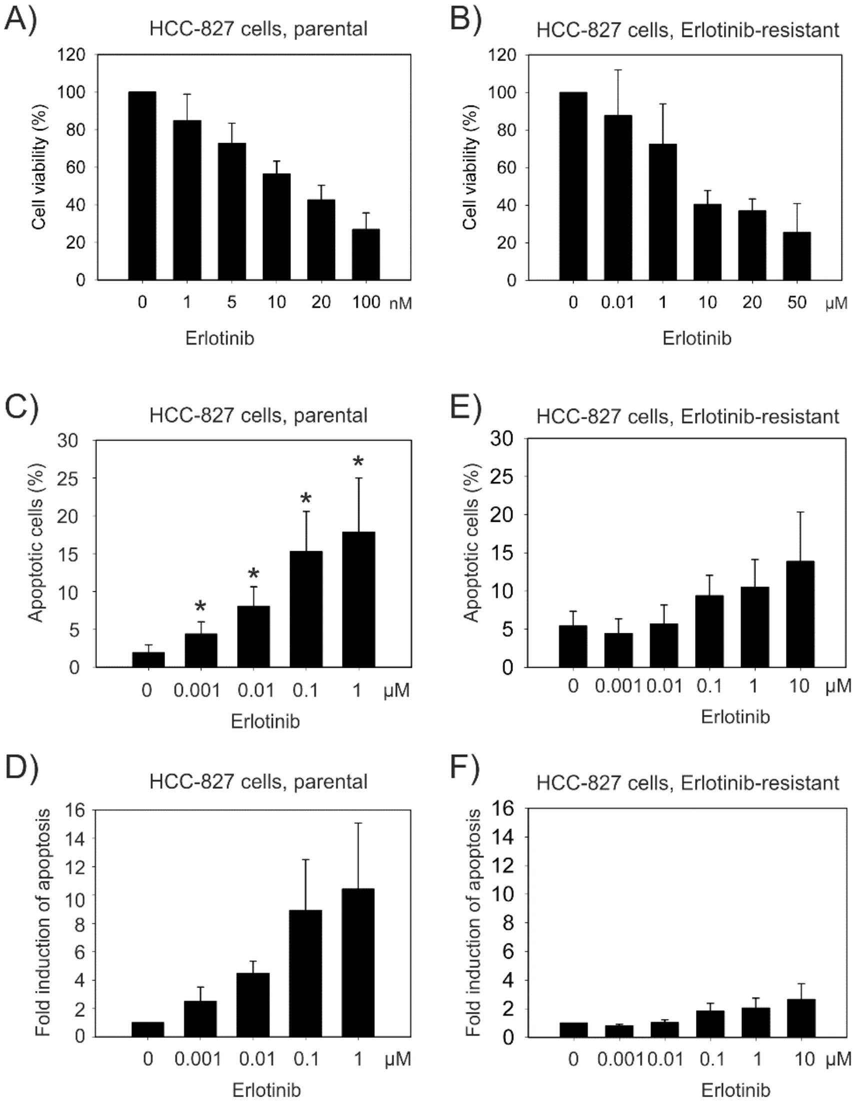

Supplementary Figure S11: Effect of Erlotinib on the cell viability of A) parental HCC-827 cells and B) Erlotinib-resistant HCC-827 cells. C, D) Effect of Erlotinib on the apoptotic rate of parental HCC-827 cells and E,F) Erlotinib-resistant HCC-827 cells. * $p<0.05$ (Student's t-test, two-tailed, paired).

Supplementary Figure S12

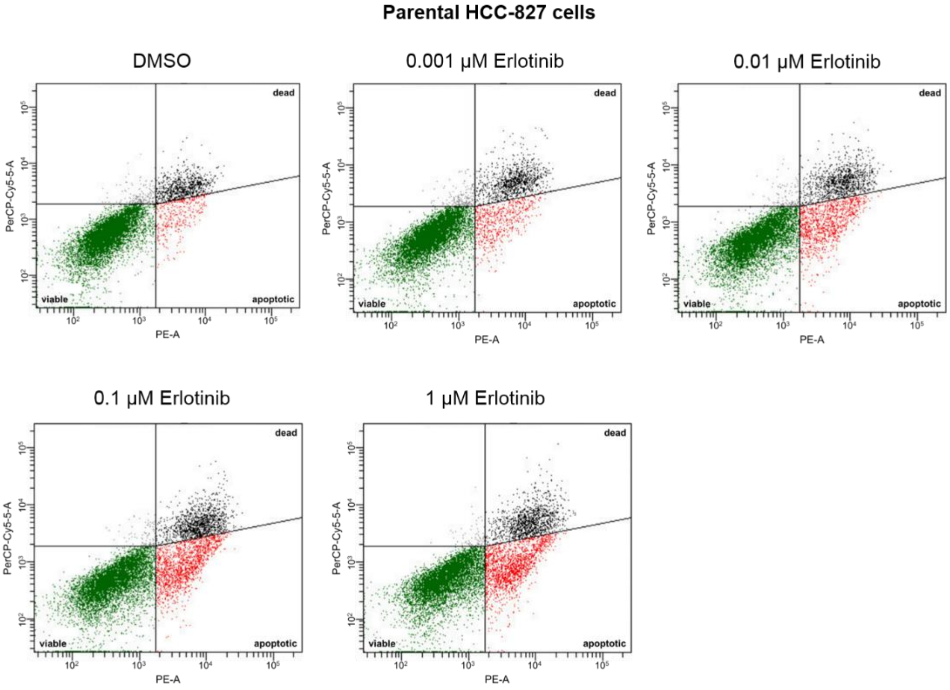

Supplementary Figure S12: Representative results of flow cytometry analysis of parental HCC-827 cells treated with the indicated concentrations of Erlotinib for 24 h. Apoptotic cells, as characterized by Annexin V staining and the absence of 7-AAD staining, are depicted in the lower right-hand quadrant.

Supplementary Figure S13

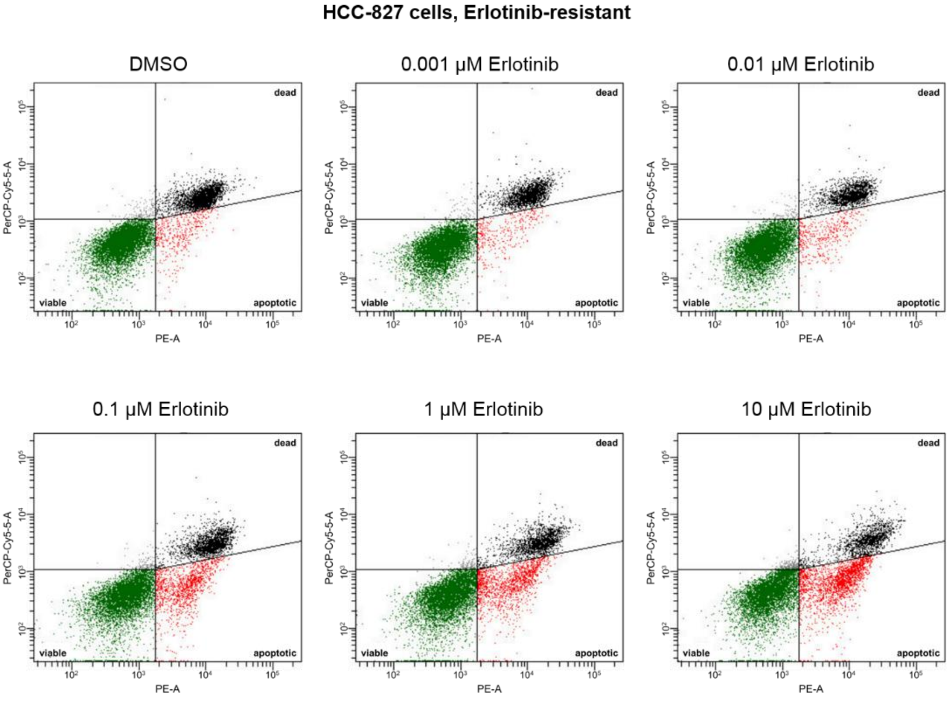

Supplementary Figure S13: Representative results of flow cytometry analysis of Erlotinib-resistant HCC-827 cells treated with the indicated concentrations of Erlotinib for 24 h. Apoptotic cells, as characterized by Annexin V staining and the absence of 7-AAD staining, are depicted in the lower right-hand section of the flow cytometry plot.

Supplementary Figure S14

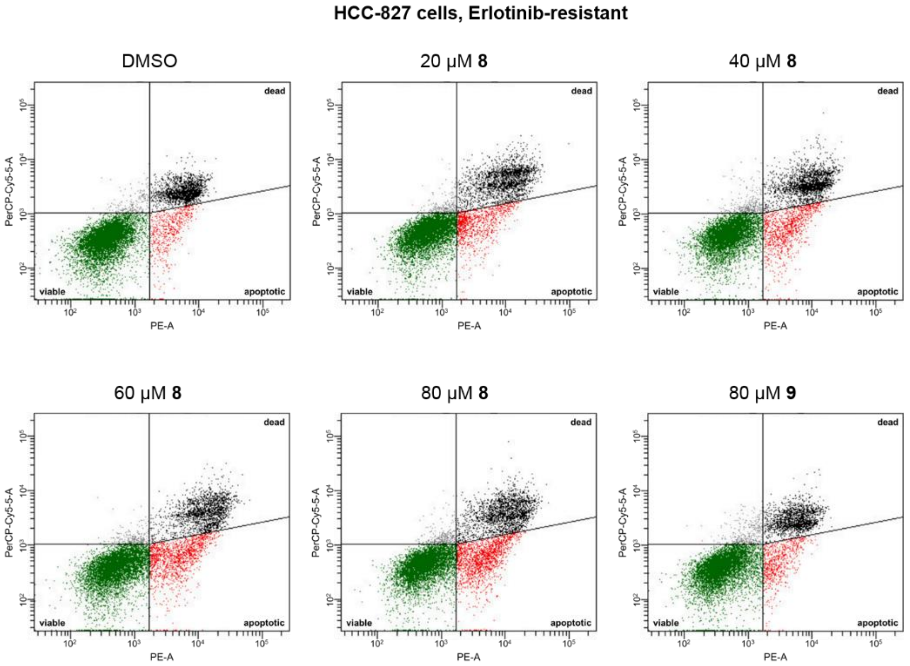

Supplementary Figure S14: Representative results of flow cytometry analysis of Erlotinib-resistant HCC-827 cells treated with the indicated concentrations of **8** and **9** for 24 h. Apoptotic cells, as characterized by Annexin V staining and the absence of 7-AAD staining, are depicted in the lower right-hand quadrant.

Supplementary Figure S15

Full-length images of the Western blot membranes shown in Fig. 3, Fig. 4, and Supplementary Fig. S10. The red boxes denote the cropped areas shown in the figures.

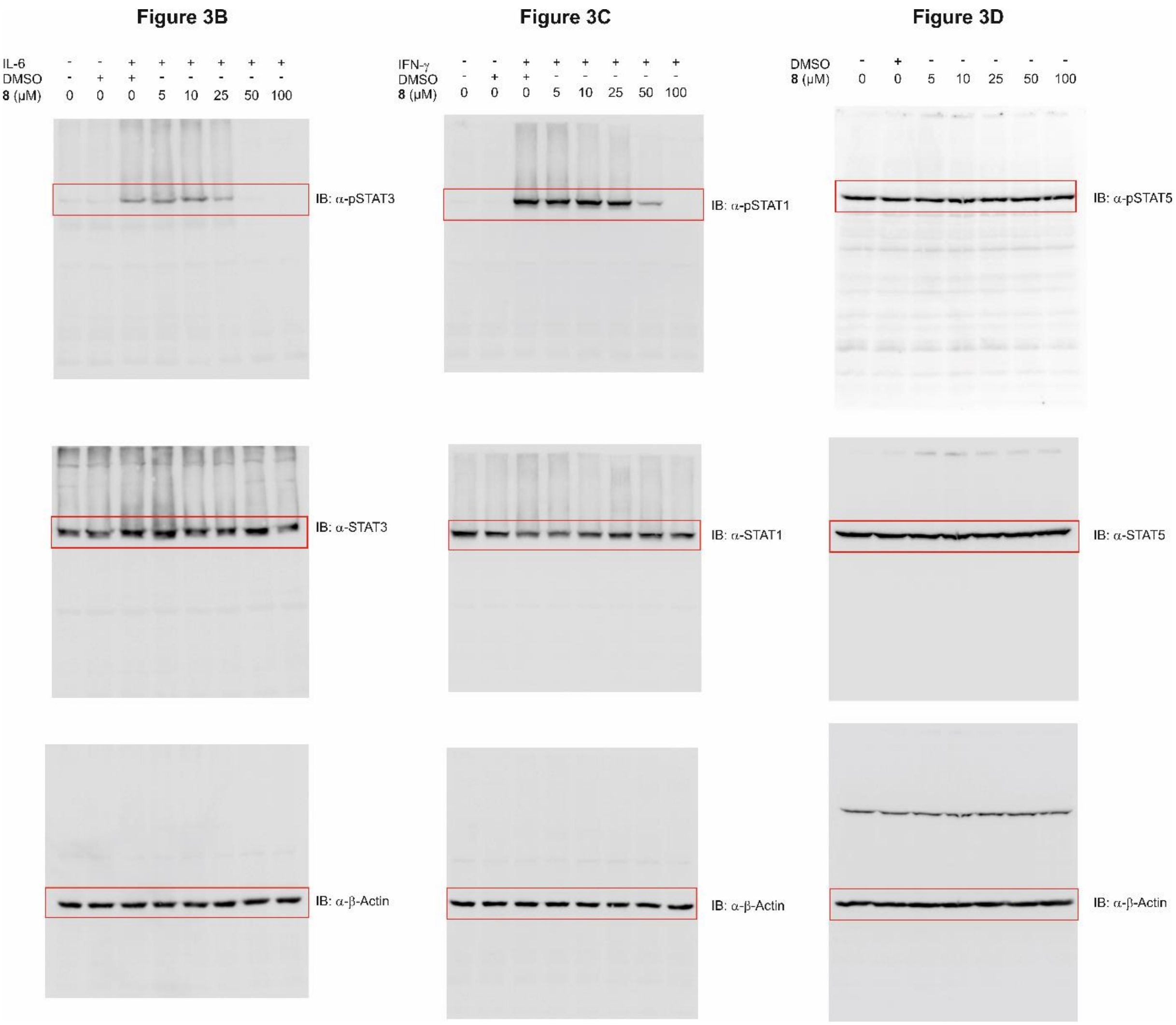

Figure 4B

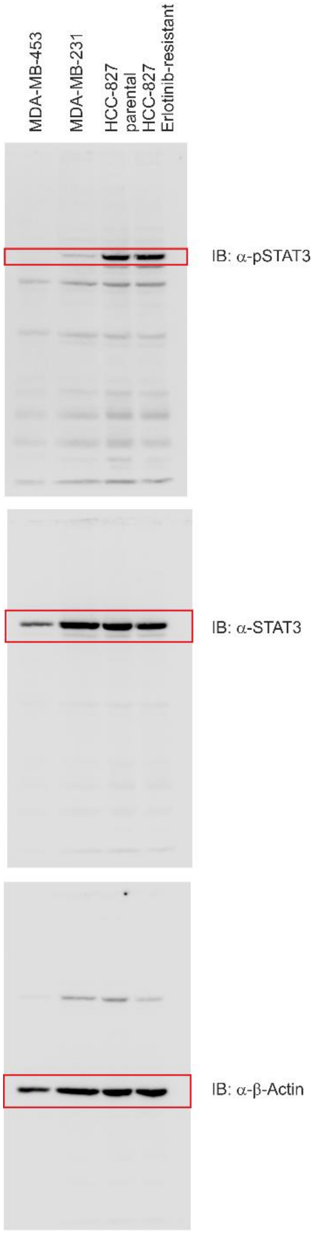

Figure 4G

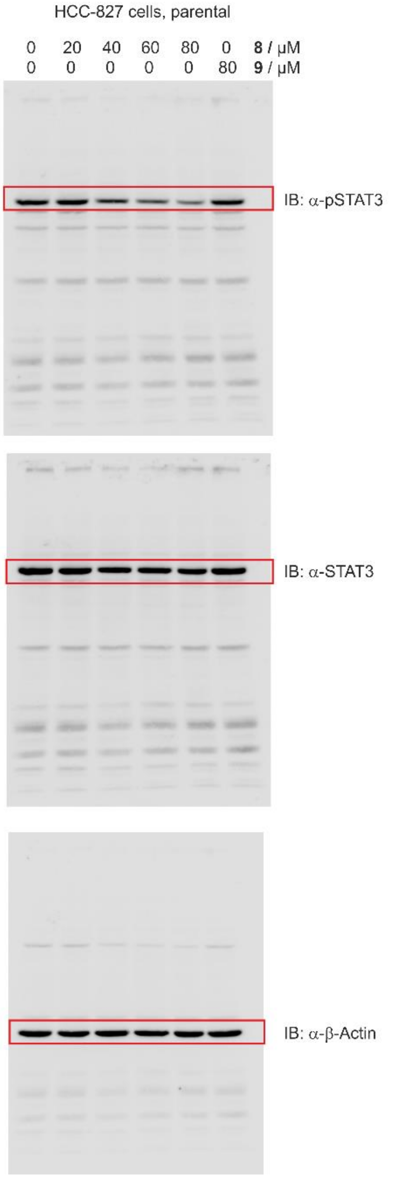

Figure 4H

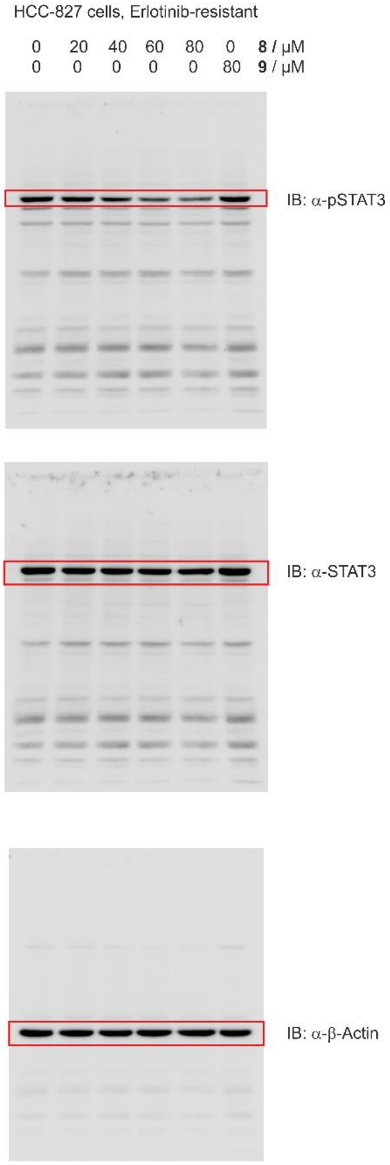

Supplementary Fig. S10

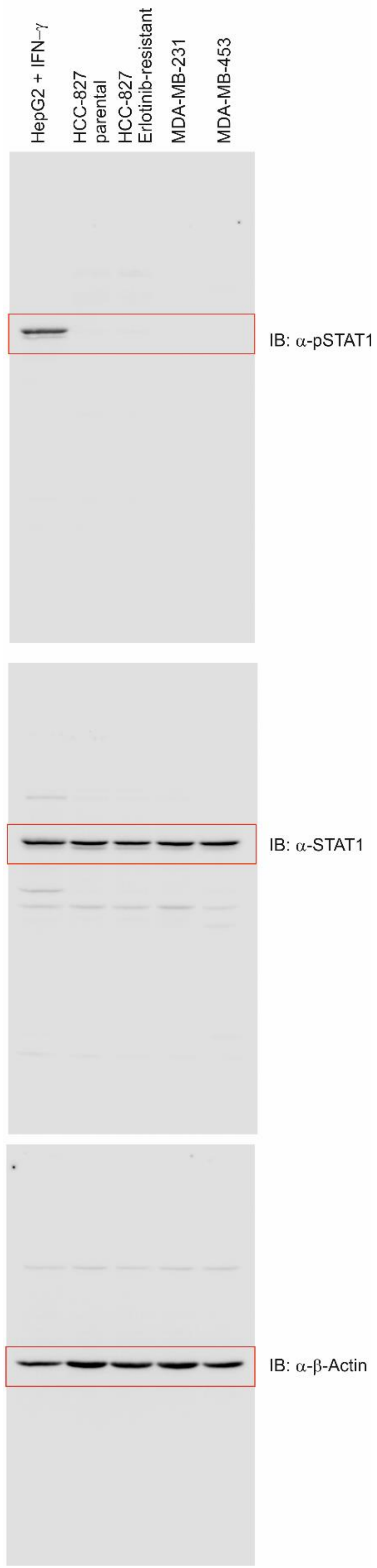

Synthesis and spectroscopic characterization of synthesized compounds

Synthesis and spectroscopic characterization of precursor molecules

1-(5-(*tert*-Butyl)-2-hydroxyphenyl)ethan-1-one (**8b**)[2,3]

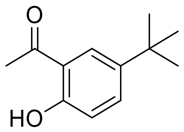

533.4 mg (4 mmol) aluminum chloride was suspended in 7.2 mL DCM. After the addition of 349 µL (2 mmol) 4-*tert*-butylanisole the reaction mixture was cooled to 0 °C. 143 µL (2 mmol) acetylchloride were added slowly and the reaction mixture was stirred for 30 min at 0 °C and for 1 h at room temperature. To quench the reaction, the mixture was poured into 10 mL of an ice/water mixture. The phases were separated. The aqueous layer was extracted with 2 x 20 mL DCM. The combined organic layers were washed with 20 mL water and 20 mL brine, dried over sodium sulfate and evaporated under reduced pressure. The oily residue was purified by column chromatography (eluent: *n*-hexane/ethyl acetate, 25:1) to provide the desired product. Yield: 272.6 mg (1.42 mmol, 71 %). ^{1}H-NMR (400 MHz, [D]Chloroform): δ = 1.32 (s, 9H), 2.65 (s, 3H), 6.93 (d, *J* = 8.8 Hz, 1H), 7.50 – 7.59 (m, 1H), 7.68 (s, 1H), 12.13 (s, 1H).

6-(*tert*-Butyl)-4-oxo-4*H*-chromene-3-carbaldehyde (**8a**)

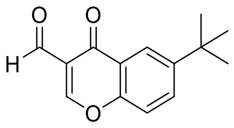

272.6 mg (1.42 mmol) **8b** was dissolved in 0.6 mL DMF and cooled to 0 °C. 278 µL (2.98 mmol) phosphoryl chloride was added slowly and the reaction mixture was stirred for 2 h at room temperature. The orange liquid was poured into 10 mL ice/water mixture and stirring was continued for 3 h. The precipitate formed was filtered off and dissolved in hot *n*-hexane/ethyl acetate 10:1. Addition of *n*-hexane and cooling to room temperature led to the crystallization of the desired product.[4] Yield: 65.8 mg (0.29 mmol, 20 %). m.p.: 102 °C. ^{1}H NMR (400 MHz, [D$_6$]DMSO) δ = 1.34 (s, 9H), 7.69 (d, *J* = 8.8 Hz, 1H), 7.91 – 8.02 (m, 1H), 8.05 (s, 1H), 8.90 (s, 1H), 10.12 (s, 1H). ^{13}C NMR (101 MHz, [D$_6$]DMSO) δ = 31.6, 35.3, 119.3, 120.5, 121.2, 124.7, 133.7, 150.1, 154.5, 163.9, 175.7, 189.2. IR (KBr): ṽ = 3440 (m), 2955 (m), 2908 (w), 2871 (w), 1699 (s), 1662 (s), 1638 (m), 1612 (m), 1600 (m), 1532 (m), 1483 (m), 1442 (m), 1397 (w), 1385 (w), 1369 (w), 1347 (w), 1332 (w), 1308 (m), 1266 (w), 1207 (w), 1167 (w), 1122 (w), 972 (w), 922 (w), 914 (w), 831 (w), 784 (w), 772 (m), 749 (w), 669 (w), 590 (w), 491 (w) cm^{-1}. UV/Vis (Methanol): λ_{max} = 340, 307, 227, 205 nm. HR-ESI-MS m/z: $C_{14}H_{14}O_3$ calcd: 253.0835 [M+Na$^+$], found: 253.0836.

4-Oxo-6-(*tert*-pentyl)-4*H*-chromene-3-carbaldehyde (**9a**)

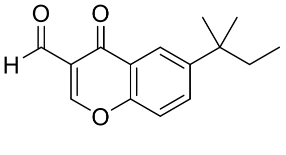

505.4 mg (2.45 mmol) 1-(2-hydroxy-5-(*tert*-pentyl)phenyl)ethan-1-one was dissolved in 1.1 mL DMF and cooled to 0 °C. 480 µL (5.15 mmol) phosphoryl chloride was added slowly and the reaction mixture was stirred for 2 h at room temperature. The yellow liquid was poured into 20 mL ice/water mixture and stirring was continued for 3 h. The precipitate formed was filtered off and recrystallized from *n*-hexane/ethyl acetate 10:1. Yield: 146.2 mg (0.60 mmol, 24 %). m.p.: 100 – 101 °C. ^{1}H NMR (300 MHz, [D$_6$]DMSO) δ = 0.62 (t, *J* = 7.4 Hz, 3H), 1.31 (s, 6H), 1.68 (q, *J* = 7.4 Hz, 2H), 7.69 (d, *J* = 8.8 Hz, 1H), 7.90 (dd, *J* = 8.9 Hz, 2.5 Hz, 1H), 8.01 (d, *J* = 2.5 Hz, 1H), 8.90 (s, 1H), 10.13 (s, 1H). ^{13}C NMR (101 MHz, [D$_6$]DMSO) δ = 8.9, 28.0, 35.9, 37.8, 118.6, 119.9, 121.3, 124.0, 133.4, 147.7, 153.8, 163.2, 175.0, 188.4. IR (KBr): ṽ = 3435 (s), 2965 (m), 2928 (w), 2876 (w), 1699 (s), 1661 (s), 1614 (s), 1603 (m), 1566 (m), 1481 (s), 1445 (w), 1386 (w), 1364 (w), 1330 (w), 1315 (m), 1246 (w), 1123 (w), 831 (w), 788 (w), 773 (m), 749 (w), 666 (w), 596 (w) cm^{-1}. UV/Vis (Acetonitrile): λ_{max} = 309, 278, 216 nm. HR-ESI-MS m/z: $C_{15}H_{16}O_3$ calcd: 267.0992 [M+Na$^+$], found: 267.0994.

4-oxo-6-phenyl-4*H*-chromene-3-carbaldehyde (**10a**)

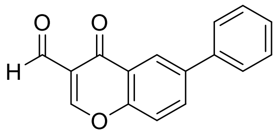

955.1 mg (4.5 mmol) 1-(4-hydroxy-[1,1'-biphenyl]-3-yl)ethan-1-one was dissolved in 2.0 mL DMF and cooled to 0 °C. 881 µL (9.45 mmol) phosphoryl chloride was added slowly and the reaction mixture was stirred for 1 h at room temperature. The yellow liquid was poured into 40 mL ice/water mixture and stirring was continued for 3 h. The precipitate formed was filtered off and purified using column chromatography (eluent: *n*-hexane/ethyl acetate, 7:1 → 3:1) to provide the desired product. Yield: 631.8 mg (2.525 mmol, 56 %). m.p.: 165 – 167 °C. ^{1}H NMR (400 MHz, [D$_6$]DMSO): δ = 7.44 (t, *J* = 7.3 Hz, 1H), 7.53 (t, *J* = 7.5 Hz, 2H), 7.76 (d, *J* = 7.8 Hz, 2H), 7.85 (d, *J* = 8.7 Hz, 1H), 8.18 (dd, *J* = 8.8, 2.2 Hz, 1H), 8.31 (d, *J* = 2.0 Hz, 1H). ^{13}C NMR (75 MHz, [D$_6$]DMSO) δ = 119.7, 120.0, 122.4, 124.9, 127.0, 128.2, 129.2, 133.5, 138.1, 138.5, 155.1, 163.3, 174.8, 188.3. IR (KBr): ṽ = 3435 (m), 3065 (w), 3041 (w), 2924 (w), 2849 (w), 1734 (w), 1698 (s), 1652 (s), 1614 (s), 1601 (m), 1566 (m), 1509 (w), 1471 (s), 1460 (m), 1434 (m), 1352 (w), 1318 (s), 1281 (m), 1267 (w), 1166 (w), 958 (w), 876 (w), 824 (w), 775 (m), 760 (m), 700 (w), 686 (m), 659 (w), 571 (w), 509 (w) cm^{-1}. UV/Vis (Acetonitrile): λ_{max} = 295, 244 nm. HR-ESI-MS m/z: $C_{16}H_{10}O_3$ calcd: 273.0522 [M+Na$^+$], found: 273.0523.

6-Methoxy-4-oxo-4*H*-chromene-3-carbaldehyde (**11a**)

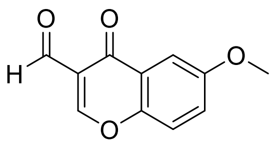

1.0 g (6.02 mmol) 1-(2-hydroxy-5-methoxyphenyl)ethan-1-one was dissolved in 2.8 mL DMF and cooled to 0 °C. 1.18 mL (12.64 mmol) phosphoryl chloride was added slowly and the reaction mixture was stirred for 1.5 h at room temperature. The highly viscous brown mixture was poured into 20 mL ice/water mixture and stirring was continued for 3 h. This mixture was diluted with 80 mL ethyl acetate, the phases were separated and the aqueous phase was extracted with 2 x 40 mL ethyl acetate. The combined organic layers were dried over Na_2SO_4

and concentrated at reduced pressure yielding a yellow solid that was suspended in 15 mL of methanol and stirred for several minutes, during which the product precipitated as a white crystalline solid, which was collected by filtration and dried *in vacuo*. Yield: 244 mg (1.20 mmol, 20 %). ^{1}H NMR (300 MHz, DMSO-d$_6$): δ = 10.16 (s, 1H), 8.92 (s, 1H), 7.76 (dd, J = 9.0 Hz, 0.5 Hz, 1H), 7.55-7.46 (m, 2H), 3.91 ppm (s, 3H). Conforms to literature.[5]

Synthesis and spectroscopic characterization of Chromone-Based Acyl Hydrazones

Compounds **1, 2, 6** were synthesized as described.[6]

(*E*)-*N*'-((6-chloro-4-oxo-4*H*-chromen-3-yl)methylene)nicotinohydrazide (**3**)

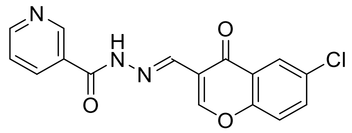

500 mg (2.40 mmol) 6-chloro-4-oxo-4*H*-chromene-3-carbaldehyde and 329 mg (2.40 mmol) nicotinic hydrazide were suspended in 160 mL of ethanol, to which were added 8 mL of water and 5 mL of glacial acetic acid. The mixture was stirred under reflux for 5 hours. After cooling to room temperature, solvents were removed under reduced pressure to give a yellow solid, which was recrystallized from ethanol and dried *in vacuo*. Yield: 207 mg (0.63 mmol, 26 %). m.p.: 213 – 217 ºC. ^{1}H NMR (400 MHz, DMSO-d$_6$): δ = 12.11 (s, 1H), 9.08 (d, J = 1.5 Hz, 1H), 8.89 (s, 1H), 8.77 (d, J = 3.5 Hz, 1H), 8.61 (s, 1H), 8.27 (d, J = 8.0 Hz, 1H), 8.08 (d, J = 2.5 Hz, 1H), 7.92 (dd, J = 9.0 Hz, 2.6 Hz, 1H), 7.83 (d, J = 9.0 Hz, 1H), 7.58 (dd, J = 7.7 Hz, 4.9 Hz, 1H). ^{13}C NMR (100 MHz, DMSO-d$_6$): δ = 174.0, 161.5, 154.4, 152.4, 150.1, 148.6, 140.7, 135.4, 134.6, 130.6, 128.9, 124.5, 124.2, 123.6, 121.3, 118.3. IR (KBr): ṽ = 3446 (br), 3064 (m), 2870 (m), 2359 (w), 2330 (w), 1705 (m), 1695 (s), 1655 (s), 1620 (s), 1590 (m), 1550 (s), 1440 (s), 1360 (s), 1340 (s), 1305 (s), 1270 (w), 1234 (m), 1222 (m), 1193 (m), 1102 (w), 962 (m), 941 (m), 928 (m), 887 (w), 841 (m), 797 (m), 780 (m), 770 (s), 716 (m) , 690 (m), 556 (m), 495 (m), 416 (m) cm^{-1}. UV/Vis (MeOH): λ_{max} = 221, 280, 310 nm. ESI-MS m/z: $C_{16}H_{10}ClN_3O_3$ calcd: 350.0 [M+Na$^+$], found: 350.0. Elemental Analysis: found: C 58.43 %, H 3.15 %, N 12.70 %. calcd. for $C_{16}H_{10}ClN_3O_3$: C 58.64 %, H 3.08 %, N 12.82 %.

(*E*)-*N*'-((6-bromo-4-oxo-4*H*-chromen-3-yl)methylene)nicotinohydrazide (**4**)

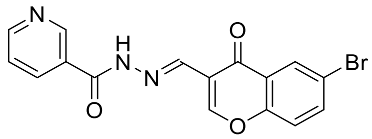

400 mg (1.58 mmol) 6-bromo-4-oxo-4*H*-chromene-3-carbaldehyde and 217 mg (1.58 mmol) nicotinic hydrazide were suspended in 105 mL of ethanol, to which were added 5 mL of water and 3 mL of glacial acetic acid. The mixture was stirred under reflux for 4.5 hours. After cooling to room temperature, solvents were removed under reduced pressure to give a yellow solid, which was recrystallized from ethanol and dried *in vacuo*. Yield: 111 mg (0.30 mmol, 19 %). m.p.: 226 ºC. ^{1}H NMR (400 MHz, DMSO-d$_6$): δ = 12.10 (s, 1H), 9.06 (s, 1H), 8.87 (s, 1H), 8.76 (d, J = 4.7 Hz, 1H), 8.59 (s, 1H), 8.25 (d, J = 7.9 Hz, 1H), 8.19 (d, J = 2.3 Hz, 1H), 8.01 (dd, J = 9.0 Hz, 2.3 Hz, 1H), 7.74 (d, J = 8.9 Hz, 1H), 7.56 (dd, J = 7.8 Hz, 4.8 Hz, 1H). ^{13}C NMR (100 MHz, DMSO-d$_6$): δ = 173.9, 161.5, 154.8, 152.4, 150.0, 148.6, 140.7, 137.3, 135.4, 128.9,

127.3, 124.9, 123.6, 121.5, 118.5, 118.4. IR (KBr): ṽ = 3240 (br), 3214 (w), 3061 m, 2360 (w), 2342 (w), 1680 (s), 1650 (s), 1595 (s), 1560 (s), 1460 (s), 1440 (s), 1380 (w), 1305 (s), 1258 (m), 1219 (m), 1197 (w), 1180 (w), 1145 (m), 1118 (m), 1028 (w), 949 (w), 897 (w), 864 (w), 832 (m), 789 (m), 704 (m), 647 (m), 627 (w), 607 (w), 544 (m), 474 (w), 417 (m) cm^{-1}. UV/Vis (MeOH): λ_{max} = 210, 283, 313 nm. ESI-MS m/z: $C_{16}H_{10}BrN_3O_3$ calcd: 394.0 [M+Na^+], found: 394.0. Elemental analysis: found: C 51.52 %, H 2.94 %, N 11.19 %. calcd. for $C_{16}H_{10}BrN_3O_3$: C 51.63 %, H 2.71 %, N 11.29 %.

(*E*)-*N'*-((6-methyl-4-oxo-4*H*-chromen-3-yl)methylene)nicotinohydrazide (**5**)

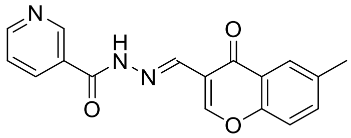

300 mg (1.59 mmol) 6-methyl-4-oxo-4*H*-chromene-3-carbaldehyde and 219 mg (1.59 mmol) of nicotinic hydrazide were suspended in 105 mL of ethanol, to which were added 5 mL of water and 4 mL of glacial acetic acid. The mixture was stirred under reflux for 5 hours. After cooling to room temperature, the mixture was concentrated under reduced pressure. The precipitate formed was filtered off, washed with cold ethanol and water, dried *in vacuo* and recrystallized from ethanol to give the product as an off-white crystalline solid that was dried *in vacuo*. Yield: 235 mg (0.77 mmol, 48 %). m.p.: 208 – 210 ºC. ^1H NMR (400 MHz, DMSO-d_6): δ = 12.07 (s, 1H), 9.08 (s, 1H), 8.84 (s, 1H), 8.77 (d, J = 3.4 Hz, 1H), 8.63 (s, 1H), 8.27 (d, J = 8.1 Hz, 1H), 7.93 (s, 1H), 7.77-7.61 (m, 2H), 7.57 (m, 1H), 2.45 (s, 3H). ^{13}C NMR (100 MHz, DMSO-d_6): δ = 175.0, 161.5, 154.6, 154.1, 152.3, 148.6, 141.2, 135.8, 135.8, 135.4, 128.9, 124.4, 123.6, 123.0, 118.5, 118.0, 20.5. IR (KBr): ṽ = 3443 (br), 3051 (w), 2360 (w), 2342 (w), 1660 (s), 1621 (s), 1594 (m), 1568 (m), 1551 (m), 1490 (s), 1433 (w), 1384 (w), 1322 (s), 1295 (m), 1238 (w), 1213 (w), 1152 (w), 1027 (w), 962 (w), 905 (w), 887 (w), 828 (m), 793 (m), 702 (m), 547 (w), 418 (w) cm^{-1}. UV/Vis (Methanol): λ_{max} = 456, 432, 408, 312, 279, 203 nm. HR-ESI-MS m/z: $C_{17}H_{13}N_3O_3$ calcd: 308.1030 [M+H^+], found: 308.1028.

(*E*)-*N'*-((6-isopropyl-4-oxo-4*H*-chromen-3-yl)methylene)nicotinohydrazide (**7**)

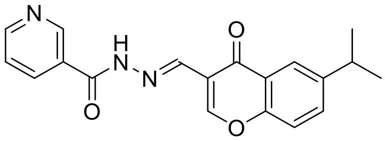

A solution of 127 mg (0.92 mmol) nicotinic hydrazide in 1.85 mL ethanol and 0.5 mL water was added dropwise to a solution of 200 mg (0.92 mmol) 6-isopropyl-4-oxo-4*H*-chromene-3-carbaldehyde in 0.93 mL chloroform. The reaction was stirred for 24 h at room temperature. The phases were separated and the organic phase was concentrated under reduced pressure to give a yellow solid which was dried *in vacuo*. The crude product was suspended in a small amount of ethyl acetate. The product precipitated as a white solid, was filtered off and dried *in vacuo*. Yield: 126 mg (0.38 mmol, 41 %). m.p.: 177 ºC. ^1H NMR (400 MHz, DMSO-d_6): δ = 12.06 (s, 1H), 9.07 (s, 1H), 8.83 (s, 1H), 8.75 (d, J = 3.4 Hz, 1H), 8.63 (s, 1H), 8.26 (d, J = 8.0 Hz, 1H), 7.95 (d, J = 2.0 Hz, 1H), 7.77 (dd, J = 8.7 Hz, 2.1 Hz, 1H), 7.66 (d, J = 8.7 Hz, 1H), 7.56 (dd, J = 7.8 Hz, 4.9 Hz, 1H), 3.07 (sept, J = 6.9 Hz, 1H), 1.25 (d, J = 6.9 Hz, 6H). ^{13}C NMR (100 MHz, DMSO-d_6): δ = 175.0, 161.4, 154.5, 154.2, 152.3, 148.6, 146.4, 141.2, 135.4, 133.5, 128.9, 123.6, 123.0, 121.6, 118.7, 117.9, 32.9, 23.7. IR (KBr): ṽ = 3445 (br), 3050 (w), 2962 (w), 2871 (w), 2360 (w), 2342 (w), 1654 (s), 1619 (m), 1595 (m), 1568 (m), 1482 (s),

1449 (w), 1420 (w), 1384 (w), 1312 (m), 1243 (w), 1213 (w), 1149 (m), 1067 (w), 1026 (w), 961 (w), 866 (w), 826 (m), 793 (m), 707 (m), 594 (w) cm^{-1}. UV/Vis (Methanol): λ_{max} = 411, 310, 278, 205 nm. HR-ESI-MS m/z: $C_{19}H_{17}N_3O_3$ calcd: 336.1343 [M+H$^+$], found: 336.1342.

(*E*)-*N*'-((6-(*tert*-butyl)-4-oxo-4*H*-chromen-3-yl)methylene)nicotinohydrazide (**8**)

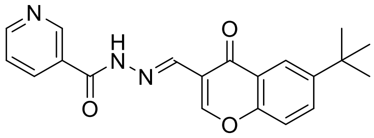

A solution of 54.9 mg (0.4 mmol) nicotinic hydrazide in 0.8 mL ethanol and 0.2 mL water was added dropwise to a suspension of 92.1 mg (0.40 mmol) **8a** in 0.4 mL of chloroform. The reaction was stirred for 24 h at room temperature. The solvent was removed *in vacuo* and the residue was recrystallized from ethyl acetate. Yield: 85.4 mg (0.24 mmol, 61 %) m.p.: 206 – 207 ºC. ^{1}H NMR (400 MHz, DMSO-d$_6$): δ = 12.07 (s, 1H), 9.06 (d, J = 1.4 Hz, 1H), 8.82 (s, 1H), 8.75 (d, J = 3.6 Hz, 1H), 8.62 (s, 1H), 8.26 (d, J = 8.0 Hz, 1H), 8.04 (d, J = 2.3 Hz, 1H), 7.93 (dd, J = 8.8 Hz, 2.4 Hz, 1H), 7.65 (d, J = 8.9 Hz, 1H), 7.55 (dd, J = 7.8 Hz, 4.9 Hz, 1H), 1.33 (s, 9H). ^{13}C NMR (100 MHz, DMSO-d$_6$): δ = 175.1, 161.4, 154.5, 154.0, 152.3, 148.7, 148.6, 141.2, 135.4, 132.6, 128.9, 123.6, 122.6, 120.3, 118.5, 118.0, 35.0, 30.9. IR (KBr): ṽ = 3445 (br), 3251 (m), 3051 (w), 2963 (m), 2907 (w), 2869 (w), 1660 (s), 1620 (s), 1596 (m), 1570 (m), 1480 (s), 1466 (w), 1440 (w), 1419 (w), 1365 (m), 1320 (s), 1294 (m), 1264 (s), 1225 (w), 1207 (w), 1186 (w), 1149 (m), 1124 (m), 1067 (w), 1026 (w), 959 (w), 904 (w), 884 (w), 825 (m), 794 (w), 735 (w), 707 (m), 663 (w), 622 (w), 599 (m), 509 (w), 418 (w) cm^{-1}. UV/Vis (Methanol): λ_{max} = 458, 430, 407, 309, 276, 231, 202 nm. HR-ESI-MS m/z: $C_{20}H_{19}N_3O_3$ calcd: 372.1319 [M+Na$^+$], found: 372.1315.

(*E*)-*N*'-((4-oxo-6-(*tert*-pentyl)-4*H*-chromen-3-yl)methylene)nicotinohydrazide (**9**)

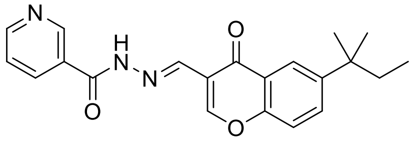

A solution of 54.9 mg (0.4 mmol) nicotinic hydrazide in 0.8 mL ethanol and 0.2 mL water was added dropwise to a solution of 97.7 mg (0.4 mmol) **9a** in 0.4 mL chloroform. The reaction was stirred for 1 h at room temperature. The precipitate formed was filtered off and recrystallized by dissolving in 5 mL ethyl acetate and subsequent addition of 5 ml *n*-hexane. Yield: 110.5 mg (0.3 mmol, 76 %). m.p.: 169 – 171 °C. ^{1}H NMR (300 MHz, [D$_6$]DMSO) δ = 0.63 (t, *J* = 7.3 Hz, 3H), 1.32 (s, 6H), 1.68 (q, *J* = 7.4 Hz, 2H), 7.57 (dd, *J* = 8.0 Hz, 4.8 Hz, 1H), 7.68 (d, *J* = 8.8 Hz, 1H), 7.88 (dd, *J* = 9.0 Hz, 2.5 Hz, 1H), 8.00 (d, *J* = 2.4 Hz, 1H), 8.18 – 8.38 (m, 1H), 8.64 (s, 1H), 8.77 (dd, *J* = 4.8 Hz, 1.7 Hz, 1H), 8.84 (s, 1H), 9.08 (s, 1H), 12.09 (s, 1H). ^{13}C NMR (75 MHz, [D$_6$]DMSO) δ = 9.0, 28.1, 35.9, 37.8, 118.0, 118.5, 121.2, 122.6, 123.6, 128.9, 132.9, 135.4, 141.2, 147.1, 148.6, 152.3, 154.0, 154.5, 161.5, 175.1. IR (KBr): ṽ = 3441 (m), 3201 (w), 3155 (w), 3066 (w), 2966 (m), 2930 (w), 2876 (w), 1736 (w), 1668 (m), 1637 (m), 1618 (m), 1591 (m), 1570 (w), 1550 (w), 1483 (m), 1443 (w), 1421 (w), 1365 (m), 1316 (m), 1301 (m), 1243 (w), 1223 (w), 1191 (w), 1149 (m), 1124 (w), 1026 (w), 954 (w), 823 (w), 826 (w), 793 (w), 736 (w), 707 (w), 635 (w), 623 (w), 600 (w) cm^{-1}. UV/Vis (Acetonitrile): λ_{max} = 308, 278, 218 nm. HR-ESI-MS m/z: $C_{21}H_{21}N_3O_3$ calcd: 364.1656 [M+H$^+$], found: 364.1659.

(*E*)-*N'*-((4-oxo-6-phenyl-4*H*-chromen-3-yl)methylene)nicotinohydrazide (**10**)

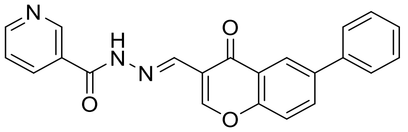

A solution of 109.7 mg (0.8 mmol) nicotinic hydrazide in 1.6 mL ethanol and 0.4 mL water was added dropwise to a solution of 97.7 mg (0.4 mmol) **10a** in 0.8 mL chloroform. The reaction was stirred for 2 h at room temperature. The precipitate formed was filtered off and subsequently washed with *n*-hexane and cold ethanol. Yield: 213.4 mg (0.58 mmol, 72 %). m.p.: 201 – 203 °C. ^{1}H NMR (300 MHz, [D$_6$]DMSO, 90 °C) δ = 7.37 – 7.48 (m, 1H), 7.49 – 7.63 (m, 3H), 7.67 – 7.87 (m, 3H), 8.13 (dd, *J* = 8.7 Hz, 2.2 Hz, 1H), 8.27 (d, *J* = 7.9 Hz, 1H), 8.33 (d, *J* = 2.1 Hz, 1H), 8.63 (s, 1H), 8.71 – 8.84 (m, 2H), 8.99 – 9.19 (m, 1H), 11.85 (s, 1H). ^{13}C NMR (101 MHz, [D$_6$]DMSO, 90 °C) δ = 118.1, 118.8, 119.0, 121.9, 122.2, 123.3, 126.4, 126.5, 128.7, 128.8, 132.4, 132.7, 137.7, 138.0, 151.4, 151.7, 153.9, 154.1, 154.9, 174.4. IR (KBr): ṽ = 3435 (s), 3217 (w), 3057 (w), 1639 (s), 1618 (m), 1599 (m), 1568 (w), 1543 (w), 1506 (w), 1472 (s), 1459 (w), 1430 (w), 1383 (w), 1321 (m), 1290 (w), 1265 (w), 1223 (w), 1150 (w), 1027 (w), 831 (w), 766 (w), 706 (w), 566 (w) cm^{-1}. UV/Vis (Acetonitrile): λ_{max} = 312, 286, 275, 233 nm. HR-ESI-MS m/z: $C_{22}H_{15}N_3O_3$ calcd: 392.1006 [M+Na$^+$], found: 392.1006.

(*E*)-*N'*-((6-methoxy-4-oxo-4*H*-chromen-3-yl)methylene)nicotinohydrazide (**11**)

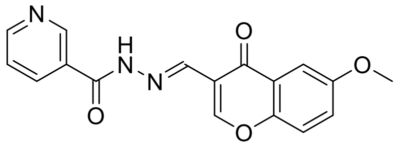

100 mg (0.49 mmol) **11a** and 67.2 mg (0.49 mmol) nicotinic hydrazide were suspended in 33 mL ethanol, to which were added 1.7 mL water and 1.0 mL glacial acetic acid. The mixture was stirred for 30 min under reflux and for 4 h at room temperature. The white precipitate formed was filtered off, washed with *n*-hexane and dried on a heating plate. Yield: 138 mg (0.43 mmol, 87 %). m.p.: 215 - 221 ºC. ^{1}H NMR (400 MHz, DMSO-d$_6$): δ = 12.06 (s, 1H), 9.06 (d, J = 1.8 Hz, 1H), 8.83 (s, 1H), 8.75 (dd, J = 4.8 Hz, 1.5 Hz, 1H), 8.63 (s, 1H), 8.32-8.20 (m, 1H), 7.70 (d, J = 9.0 Hz, 1H), 7.55 (dd, J = 7.8 Hz, 5.0 Hz, 1H), 7.51-7.39 (m, 2H), 3.87 (s, 3H). ^{13}C NMR (100 MHz, DMSO-d$_6$): δ = 174.7, 161.4, 160.6, 157.0, 152.3, 150.5, 148.6, 141.2, 135.4, 128.9, 124.0, 123.7, 123.6, 120.3, 117.3, 104.9, 55.8. IR (KBr): ṽ = 3445 (br), 3059 (w), 1660 (s), 1620 (s), 1608 (m), 1594 (m), 1567 (m), 1490 (s), 1456 (w), 1438 (w), 1387 (w), 1372 (w), 1320 (s), 1292 (m), 1253 (w), 1220 (m), 1160 (m), 1129 (w), 1027 (m), 959 (w), 908 (w), 887 (w), 832 (m), 802 (w), 789 (w), 722 (m), 700 (w), 573 (w), 485 (w), 418 (w) cm^{-1}. UV/Vis (Methanol): λ_{max} = 218 nm, 287 nm, 318 nm. HR-ESI-MS m/z: $C_{17}H_{13}N_3O_4$ calcd: 346.0798 [M+Na$^+$], found: 339.0799.

(*E*)-*N'*-((6-nitro-4-oxo-4H-chromen-3-yl)methylene)nicotinohydrazide (**12**)

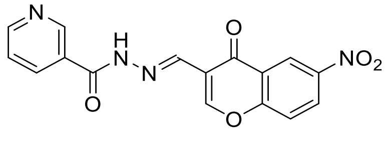

A solution of 131 mg (0.96 mmol) nicotinic hydrazide in 1.83 mL ethanol and 0.5 mL water was added dropwise to a suspension of 200 mg (0.91 mmol) 6-nitro-4-oxo-4*H*-chromene-3-

carbaldehyde in 2.00 mL chloroform. Within minutes, the yellow color intensified to orange before the entire reaction mixture solidified. It was suspended in ethanol and diluted with chloroform and water. Phases were separated. The organic phase was concentrated under reduced pressure yielding a yellow solid that was suspended in a few milliliters of ethanol. The product precipitated as a yellow solid which was filtered off and dried *in vacuo*. Yield: 147 mg (0.44 mmol, 48 %). m.p.: 233 – 239 ºC. ^{1}H NMR (400 MHz, [D$_6$]DMSO) δ = 7.51 – 7.65 (m, 1H), 8.01 (d, *J* = 9.2 Hz, 1H), 8.20 – 8.33 (m, 1H), 8.56 – 8.65 (m, 2H), 8.73 – 8.85 (m, 1H), 8.95 (s, 1H), 9.08 (s, 1H), 12.16 (s, 1H). ^{13}C NMR (101 MHz, [D$_6$]DMSO) δ = 118.8, 121.1, 121.2, 123.4, 123.7, 128.8, 128.8, 135.5, 140.2, 144.7, 148.6, 152.5, 155.3, 158.7, 161.6, 174.3. IR (KBr): ṽ = 3418 (br), 3057 (m), 2841 (w), 2359 (w), 2342 (w), 1670 (s), 1625 (s), 1290 (s), 1570 (s), 1520 (s), 1460 (s), 1422 (m), 1386 (w), 1350 (s), 1310 (s), 1252 (m), 1220 (s), 1188 (w), 1168 (m), 1146 (s), 1123 (m), 1067 (m), 1026 (m), 965 (w), 890 (m), 847 (m), 822 (m), 793 (m), 747 (m), 707 (m), 650 (m), 624 (m), 599 (w) cm^{-1}. UV/Vis (Methanol): λ_{max} = 409, 315, 282, 231 nm. HR-ESI-MS m/z: $C_{16}H_{10}N_4O_5$ calcd: 339.0724 [M+H$^+$], found: 339.0723.

(*E*)-*N'*-((6-(*tert*-butyl)-4-oxo-4*H*-chromen-3-yl)methylene)benzohydrazide (**13**)

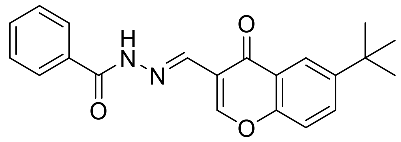

A solution of 108.9 mg (0.8 mmol) benzhydrazide in 1.6 mL ethanol and 0.4 mL water was added dropwise to a solution of 184.2 mg (0.8 mmol) **8a** in 0.8 mL chloroform. The reaction was stirred for 1 h at room temperature. The precipitate formed was filtered off and recrystallized from ethanol. Yield: 136.6 mg (0.39 mmol, 49 %). m.p.: 196 – 198 °C. ^{1}H NMR (300 MHz, [D$_6$]DMSO) δ = 1.33 (s, 9H), 7.45 – 7.64 (m, 3H), 7.65 (d, *J* = 8.9 Hz, 1H), 7.86 – 7.99 (m, 3H), 8.04 (d, *J* = 2.5 Hz, 1H), 8.63 (s, 1H), 8.80 (s, 1H), 11.93 (s, 1H). ^{13}C NMR (75 MHz, [D$_6$]DMSO) δ = 30.9, 34.6, 118.2, 118.5, 120.3, 122.6, 127.6, 128.4, 131.8, 132.5, 133.1, 140.4, 148.7, 154.0, 154.3, 162.9, 175.1. IR (KBr): ṽ = 3455 (m), 3227 (m), 3061 (m), 2963 (s), 2907 (w), 2868 (m), 2346 (w), 1648 (s), 1619 (s), 1572 (s), 1547 (s), 1484 (s), 1464 (m), 1448 (m), 1379 (m), 1365 (s), 1317 (s), 1263 (s), 1224 (m), 1207 (m), 1183 (m), 1142 (m), 1125 (m), 1102 (w), 1077 (m), 1028 (w), 1001 (w), 948 (w), 928 (w), 903 (w), 883 (w), 825 (m), 795 (m), 750 (w), 709 (m), 693 (m), 683 (m), 660 (w), 598 (m), 506 (w). UV/Vis (Methanol): λ_{max} = 405, 311, 279, 204 nm. HR-ESI-MS m/z: $C_{21}H_{20}N_2O_3$ calcd: 371.1366 [M+Na$^+$], found: 371.1367.

(*E*)-*N'*-((6-(*tert*-butyl)-4-oxo-4*H*-chromen-3-yl)methylene)-4-hydroxybenzohydrazide (**14**)

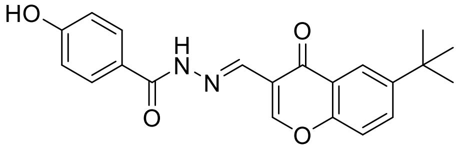

A solution of 92.1 mg (0.4 mmol) **8a** in 0.4 mL chloroform was added dropwise to a suspension of 60.9 mg (0.4 mmol) 4-hydroxybenzhydrazide in 0.8 mL ethanol and 0.2 mL water. The reaction was stirred for 3 d at room temperature and was subsequently refluxed for 3 h. The solvent was removed *in vacuo* and the residue was recrystallized from ethanol. Yield: 9 mg (0.025 mmol, 6 %). m.p.: 189 – 193 °C. ^{1}H NMR (300 MHz, [D$_6$]DMSO) δ = 1.36 (s, 9H), 6.85 (d, *J* = 8.7 Hz, 2H), 7.67 (d, *J* = 8.9 Hz, 1H), 7.82 (d, *J* = 8.7 Hz, 2H), 7.95 (dd, *J* = 8.9 Hz,

2.5 Hz, 1H), 8.06 (d, J = 2.5 Hz, 1H), 8.60 (s, 1H), 8.79 (s, 1H), 10.11 (s, 1H), 11.73 (s, 1H). ^{13}C NMR (100 MHz, [D$_6$]DMSO) δ = 31.6, 35.3, 115.7, 119.0, 119.2, 121.0, 123.3, 124.3, 130.4, 133.2, 140.1, 149.3, 154.7, 154.8, 161.4, 163.3, 175.9. IR (KBr): ṽ = 3433 (m), 3222 (m), 2962 (w), 2870 (w), 2247 (w), 2123 (w), 1644 (s), 1608 (s), 1588 (m), 1572 (m), 1558 (m), 1510 (m), 1484 (m), 1465 (w), 1442 (w), 1384 (w), 1365 (w), 1319 (m), 1278 (m), 1263 (s), 1225 (m), 1174 (m), 1143 (w), 1122 (w), 1107.9 (w), 1049 (w), 1026 (w), 1005 (m), 903 (w), 885 (w), 849 (w), 826 (w), 790 (w), 764 (w), 667 (w), 617 (w), 598 (w), 521 (w). UV/Vis (Methanol): λ_{max} = 312, 284, 215, 202 nm. HR-ESI-MS m/z: $C_{21}H_{20}N_2O_4$ calcd: 387.1315 [M+Na$^+$], found: 387.1307.

(*E*)-*N*'-((6-(*tert*-butyl)-4-oxo-4*H*-chromen-3-yl)methylene)-4-methoxybenzohydrazide (**15**)

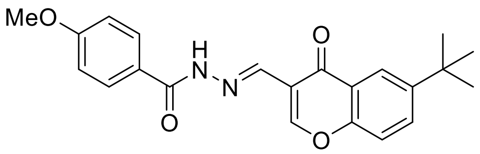

A solution of 132.9 mg (0.8 mmol) 4-methoxybenzhydrazide in 1.6 mL ethanol and 0.4 mL water was added dropwise to a solution of 184.2 mg (0.8 mmol) **8a** in 0.8 mL chloroform. The reaction was stirred for 1 h at room temperature. The precipitate formed was filtered off and recrystallized from ethanol. Yield: 189.2 mg (0.50 mmol, 63 %). m.p.: 194 – 195 °C. ^{1}H NMR (400 MHz, [D$_6$]DMSO) δ = 1.35 (s, 9H), 3.83 (s, 3H), 7.05 (d, J = 8.3 Hz, 2H), 7.66 (d, J = 8.9 Hz, 1H), 7.89 – 7.98 (m, 3H), 8.05 (d, J = 2.6 Hz, 1H), 8.62 (s, 1H), 8.81 (s, 1H), 11.84 (s, 1H). ^{13}C NMR (101 MHz, [D$_6$]DMSO) δ = 30.9, 34.6, 55.4, 113.7, 118.3, 118.5, 120.3, 122.6, 125.2, 129.6, 132.6, 139.8, 148.7, 154.0, 154.2, 162.1, 162.3, 175.2. IR (KBr): ṽ = 3476 (m), 3245 (w), 2962 (m), 2907 (w), 2869 (w), 2838 (w), 1645 (s), 1607 (s), 1571 (m), 1550 (m), 1508 (s), 1485 (s), 1465 (m), 1442 (m), 1383 (w), 1365 (m), 1316 (s), 1258 (s), 1224 (m), 1207 (w), 1176 (s), 1142 (m), 1121 (m), 1067 (w), 1029 (m), 951 (w), 903 (w), 843 (m), 825 (m), 801 (m), 795 (m), 784 (w), 763 (m), 666 (m), 648 (w), 632 (w), 611 (m), 598 (m). UV/Vis (Methanol): λ_{max} = 407, 308, 283, 238, 207, 205 nm. HR-ESI-MS m/z: $C_{22}H_{22}N_2O_4$ calcd: 401.1472 [M+Na$^+$], found: 401.1471.

(*E*)-*N*'-((6-(*tert*-butyl)-4-oxo-4*H*-chromen-3-yl)methylene)-3-methoxybenzohydrazide (**16**)

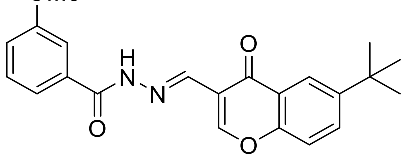

A solution of 132.9 mg (0.8 mmol) 3-methoxybenzhydrazide in 1.6 mL ethanol and 0.4 mL water was added dropwise to a solution of 184.2 mg (0.8 mmol) **8a** in 0.8 mL chloroform. The reaction was stirred for 4 h at room temperature. The precipitate formed was filtered off and recrystallized from ethanol. Yield: 98 mg (0.26 mmol, 33 %). m.p.: 118 – 120 °C. ^{1}H NMR (400 MHz, [D$_6$]DMSO) δ = 1.35 (s, 9H), 3.84 (s, 3H), 7.16 (d, J = 8.0 Hz, 1H), 7.38 – 7.55 (m, 3H), 7.68 (d, J = 8.8 Hz, 1H), 7.95 (dd, J = 8.9 Hz, 2.5 Hz, 1H), 8.06 (s, 1H), 8.65 (s, 1H), 8.83 (s, 1H), 11.91 (s, 1H). ^{13}C NMR (100 MHz, [D$_6$]DMSO) δ = 31.4, 35.1, 55.8, 113.2, 118.2, 118.6, 119.0, 120.4, 120.8, 123.1, 130.1, 133.1, 135.0, 141.0, 149.2, 154.5, 154.9, 159.7, 163.1, 175.7. IR (KBr): ṽ = 3440 (m), 3227 (w), 3060 (w), 2961 (w), 2870 (w), 2835 (w), 1652 (s), 1619 (s), 1581 (s), 1552 (m), 1485 (s), 1465 (m), 1434 (m), 1365 (m), 1318 (s), 1287 (s),

1264 (s), 1243 (m), 1222 (m), 1206 (w), 1182 (w), 1124 (m), 1079 (w), 1044 (m), 995 (w), 950 (w), 909 (w), 879 (w), 826 (m), 797 (m), 748 (m), 688 (w), 675 (w), 659 (w), 643 (w), 598 (w), 507 (w), 455 (w), 404 (w). UV/Vis (Ethanol): λ_{max} = 313, 280, 211, 204 nm. HR-ESI-MS m/z: $C_{22}H_{22}N_2O_4$ calcd: 401.1472 [M+Na$^+$], found: 401.1466.

(*E*)-*N*'-((6-(*tert*-butyl)-4-oxo-4*H*-chromen-3-yl)methylene)furan-2-carbohydrazide (**17**)

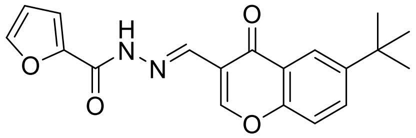

A solution of 25.2 mg (0.2 mmol) 2-furoic hydrazide in 0.4 mL ethanol and 0.1 mL water was added dropwise to a solution of 46.1 mg (0.2 mmol) **8a** in 0.2 mL chloroform. The reaction was stirred for 15 h at room temperature. The precipitate formed was filtered off and recrystallized from ethanol. Yield: 40.1 mg (0.12 mmol, 59 %). m.p.: 183 – 185 °C. ^{1}H NMR (400 MHz, [D$_6$]DMSO) δ = 1.36 (s, 9H), 6.70 (dd, *J* = 3.5 Hz, 1.7 Hz, 1H), 7.32 (s, 1H), 7.68 (d, *J* = 8.8 Hz, 1H), 7.90 – 7.99 (m, 2H), 8.06 (d, *J* = 2.5 Hz, 1H), 8.62 (s, 1H), 8.82 (s, 1H), 11.96 (s, 1H); ^{13}C NMR (75 MHz, [D$_6$]DMSO) δ = 30.9, 34.6, 109.6, 112.1, 115.0, 118.1, 118.5, 120.4, 122.7, 132.6, 140.5, 146.0, 146.4, 148.7, 154.0, 154.5, 175.1; IR (KBr): $\tilde{\nu}$ = 3442 (m), 3256 (w), 2964 (w), 2867 (w), 1647 (s), 1620 (m), 1568 (w), 1542 (w), 1485 (m), 1471 (w), 1440 (w), 1407 (w), 1396 (w), 1384 (w), 1365 (w), 1316 (w), 1265 (w), 1226 (w), 1190 (w), 1173 (w), 1140 (w), 1083 (w), 1020 (w), 947 (w), 885 (w), 857 (w), 827 (w), 784 (w), 759 (w), 665 (w), 608 (w), 597 (w). UV/Vis (Ethanol): λ_{max} = 314, 283, 218, 203 nm. HR-ESI-MS m/z: $C_{19}H_{18}N_2O_4$ calcd: 361.1159 [M+Na$^+$], found: 361.1159.

(*E*)-*N*'-((6-(*tert*-butyl)-4-oxo-4*H*-chromen-3-yl)methylene)thiophene-2-carbohydrazide (**18**)

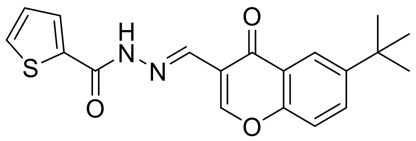

A solution of 56.9 mg (0.4 mmol) 2-thiophenecarboxylic acid hydrazide in 0.8 mL ethanol and 0.2 mL water was added dropwise to a solution of 92.1 mg (0.4 mmol) 8a in 0.4 mL chloroform. The reaction was stirred for 10 min at room temperature. The precipitate formed was filtered off and recrystallized from ethanol. Yield: 15.9 mg (0.05 mmol, 11 %). m.p.: 192 – 196 °C. ^{1}H NMR (400 MHz, [D$_4$]Methanol) δ = 1.41 (s, 9H), 7.20 (t, *J* = 4.4 Hz, 1H), 7.60 (d, *J* = 8.9 Hz, 1H), 7.77 (d, *J* = 5.0 Hz, 1H), 7.81 – 7.90 (m, 1H), 7.94 (dd, *J* = 8.9 Hz, 2.5 Hz, 1H), 8.21 (d, *J* = 2.5 Hz, 1H), 8.56 (s, 1H), 8.99 (s, 1H). ^{13}C NMR (100 MHz, [D$_6$]DMSO) δ = 30.9, 34.6, 118.1, 118.5, 120.4, 122.6, 128.2, 129.1, 132.0, 132.6, 136.5, 138.1, 140.2, 148.7, 154.0, 154.5, 175.2. IR (KBr): $\tilde{\nu}$ = 3446 (m), 2962 (w), 1655 (m), 1618 (m), 1570 (w), 1558 (w), 1542 (w), 1517 (w), 1485 (w), 1465 (w), 1438 (w), 1419 (w), 1380 (w), 1315 (w), 1265 (w), 1224 (w), 1150 (w), 1122 (w), 844 (w), 828 (w), 793 (w),734 (w), 602 (w), 515 (w), 496 (w), 458 (w), 438 (w). UV/Vis (Ethanol): λ_{max} = 315, 284, 275, 253, 235, 223, 203 nm. HR-ESI-MS m/z: $C_{19}H_{18}N_2O_3S$ calcd: 377.0930 [M+Na$^+$], found: 377.0928.

(*E*)-*N*'-((6-(*tert*-butyl)-4-oxo-4*H*-chromen-3-yl)methylene)-1*H*-pyrrole-2-carbohydrazide (**19)**

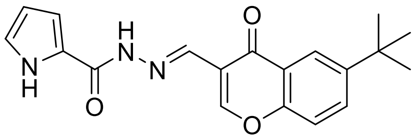

A solution of 92.1 mg (0.4 mmol) 8a in 0.4 mL chloroform was added dropwise to a suspension of 50.1 mg (0.4 mmol) 1H-pyrrole-2-carbohydrazide hydrazide in 0.8 mL ethanol and 0.2 mL water. The reaction was stirred for 45 min at room temperature. The precipitate formed was filtered off and recrystallized from ethanol. Yield: 59.9 mg (0.17 mmol, 43 %). m.p.: 140 – 142 °C. ^{1}H NMR (300 MHz, [D_6]DMSO) δ = 1.36 (s, 9H), 6.10 – 6.22 (m, 1H), 7.00 (s, 2H), 7.67 (d, *J* = 8.8 Hz, 1H), 7.94 (dd, *J* = 8.9 Hz, 2.5 Hz, 1H), 8.07 (d, *J* = 2.5 Hz, 1H), 8.53 (s, 1H), 8.80 (s, 1H), 11.53 (s, 1H), 11.78 (s, 1H). ^{13}C NMR (100 MHz, [D_6]DMSO) δ = 31.0, 34.6, 109.1, 109.5, 111.3, 118.3, 118.5, 120.4, 122.7, 122.9, 124.4, 132.6, 138.2, 148.7, 154.0, 154.2, 175.2. IR (KBr): ṽ = 3435 (m), 3256 (w), 2963 (w), 2870 (w), 2361 (w), 1716 (w), 1698 (w), 1653 (w), 1618 (w), 1570 (w), 1558 (w), 1543 (w), 1507 (w), 1485 (w), 1457 (w), 1422 (w), 1363 (w), 1317 (w), 1264 (w), 1200 (w), 1180 (w), 1128 (w), 1034 (w), 857 (w), 828 (w), 793 (w), 745 (w), 690 (w), 665 (w), 647 (w), 614 (w), 598 (w), 533 (w), 516 (w). UV/Vis (Ethanol): λ_{max} = 318, 290, 250, 219 nm. HR-ESI-MS m/z: $C_{19}H_{19}N3O_3$ calcd: 360.1319 [M+Na$^+$], found: 360.1318.

(*E*)-*N*'-((6-(*tert*-butyl)-4-oxo-4*H*-chromen-3-yl)methylene)-4-fluorobenzohydrazide (**20**)

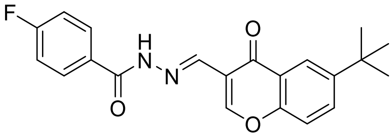

A solution of 123.3 mg (0.8 mmol) 4-fluorobenzhydrazide in 1.6 mL ethanol and 0.4 mL water was added dropwise to a solution of 184.2 mg (0.8 mmol) **8a** in 0.8 mL chloroform. The reaction was stirred for 1 h at room temperature. The precipitate formed was filtered off and recrystallized from ethanol. Yield: 0.215 g (0.59 mmol, 74 %). m.p.: 195 – 196 °C. ^{1}H NMR (400 MHz, [D_6]DMSO) δ = 1.36 (s, 9H), 7.38 (t, *J* = 8.7 Hz, 2H), 7.68 (d, *J* = 8.9 Hz, 1H), 7.95 (dd, *J* = 9.0 Hz, 2.5 Hz, 1H), 8.02 (dd, *J* = 8.5 Hz, 5.7 Hz, 2H), 8.06 (d, *J* = 2.4 Hz, 1H), 8.64 (s, 1H), 8.83 (s, 1H), 11.98 (s, 1H). ^{13}C NMR (101 MHz, [D_6]DMSO) δ = 31.38 , 35.08 , 115.96 (d, *J*=21.8), 118.59, 118.97, 120.80, 123.10, 130.07, 130.84 (d, *J*=9.1), 133.08, 141.04, 149.18, 154.51, 154.87, 162.29, 164.67 (d, *J* = 249.6 Hz), 175.64. ^{19}F NMR (377 MHz, [D_6]DMSO) δ = -108.45 – -108.10 (m). IR (KBr): ṽ = 3454 (m), 3199 (w), 3069 (w), 3039 (w), 2965 (m), 2908 (w), 2870 (w), 1650 (s), 1619 (s), 1605 (s), 1570 (m), 1553 (m), 1506 (s), 1484 (s), 1465 (m), 1441 (w), 1382 (w), 1366 (m), 1317 (s), 1279 (m), 1263 (s), 1234 (m), 1228 (m), 1208 (w), 1184 (w), 1160 (w), 1143 (m), 1125 (m), 1099 (w), 1067 (w), 945 (w), 903 (w), 850 (m), 826 (m), 813 (w), 794 (w), 760 (m), 666 (m), 605 (m), 599 (w), 577 (w), 516 (w), 506 (w). UV/Vis (Methanol): λ_{max} = 405, 310, 279, 231, 206 nm. HR-ESI-MS m/z: $C_{21}H_{19}FN_2O_3$ calcd: 389.1272 [M+Na$^+$], found: 389.1271.

(*E*)-*N*'-((6-(*tert*-butyl)-4-oxo-4*H*-chromen-3-yl)methylene)-4-chlorobenzohydrazide (**21**)

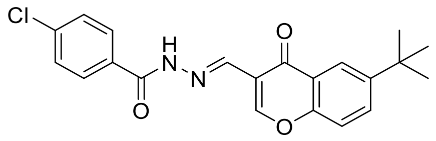

A solution of 136.5 mg (0.8 mmol) 4-chlorobenzhydrazide in 1.6 mL ethanol and 0.4 mL water was added dropwise to a solution of 184.2 mg (0.8 mmol) **8a** in 0.8 mL chloroform. The reaction was stirred for 1 h at room temperature. The precipitate formed was filtered off and recrystallized from ethanol. Yield: 0.251 g (0.65 mmol, 81 %). m.p.: 202 – 204 °C. ^{1}H NMR (300 MHz, [D$_6$]DMSO) δ = 1.35 (s, 9H), 7.60 (d, *J* = 8.4 Hz, 2H), 7.66 (d, *J* = 8.9 Hz, 1H), 7.90 – 8.01 (m, 3H), 8.05 (d, *J* = 2.5 Hz, 1H), 8.64 (s, 1H), 8.82 (s, 1H), 11.99 (s, 1H). ^{13}C NMR (75 MHz, [D$_6$]DMSO) δ = 30.9, 34.6, 118.1, 118.5, 120.3, 122.6, 128.6, 129.6, 131.8, 132.6, 136.7, 140.8, 148.7, 154.0, 154.4, 161.8, 175.1. IR (KBr): ṽ = 3476 (m), 3433 (m), 3209 (w), 3069 (w), 2963 (m), 2906 (w), 2869 (w), 1658 (s), 1637 (s), 1619 (s), 1599 (s), 1568 (s), 1550 (m), 1485 (s), 1464 (m), 1440 (m), 1398 (w), 1382 (w), 1365 (w), 1316 (s), 1263 (s), 1223 (m), 1206 (w), 1181 (m), 1146 (m), 1127 (m), 1113 (m), 1095 (m), 1069 (w), 1014 (m), 943 (w), 913 (w), 902 (m), 846 (m), 824 (m), 804 (m), 793 (m), 783 (m), 752 (m), 664 (m), 598 (m), 531 (w), 505 (w), 457 (w). UV/Vis (Methanol): λ_{max} = 405, 308, 282, 238, 206 nm. HR-ESI-MS m/z: $C_{21}H_{19}ClN_2O_3$ calcd: 383.1157 [M+H$^+$], found: 383.1155.

(*E*)-3-((2-benzylhydrazono)methyl)-6-(*tert*-butyl)-4*H*-chromen-4-one (**22**)

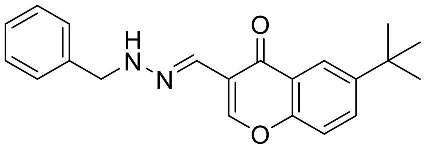

78 mg (0.4 mmol) benzylhydrazin dihydrochloride and 78.7 mg (0.96 mmol) sodium acetate were dissolved in 0.6 mL water. The obtained solution was added to a solution of 92.1 mg (0.4 mmol) **8a** in 1.2 mL ethanol. The reaction was stirred for 3 h at room temperature. The solvent was removed *in vacuo*. The residue was purified by column chromatography (eluent: *n*-hexane/ethyl acetate, 10:1 → 2:1) to provide the desired product. Yield: 17 mg (0.05 mmol, 13 %).[7] m.p.: 126 – 128 °C. ^{1}H NMR (400 MHz, [D$_6$]DMSO) δ = 1.15 (s, 9H), 5.12 (d, *J* = 15.7 Hz, 1H), 5.31 (d, *J* = 15.4 Hz, 1H), 6.88 – 7.02 (m, 3H), 7.09 (d, *J* = 2.5 Hz, 1H), 7.17 – 7.33 (m, 3H), 7.38 (dd, *J* = 8.6 Hz, 2.5 Hz, 1H), 8.05 (s, 1H), 9.46 (s, 1H), 10.03 (s, 1H). ^{13}C NMR (100 MHz, [D$_6$]DMSO) δ = 33.0, 35.5, 54.7, 114.9, 117.6, 123.4, 129.0, 129.3, 130.2, 130.3, 138.5, 140.7, 143.3, 147.9, 154.8, 186.5. IR (KBr): ṽ = 3418 (m), 3127 (w), 3065 (w), 3033 (w), 2961 (m), 2906 (w), 2866 (w), 1678 (s), 1655 (s), 1614 (m), 1541 (w), 1508 (m), 1485 (s), 1456 (w), 1408 (m), 1383 (m), 1362 (w), 1293 (m), 1279 (m), 1203 (m), 1149 (w), 1130 (w), 1077 (w), 1030 (w), 893 (w), 876 (w), 825 (m), 801 (w), 781 (m), 727 (m), 696 (w), 660 (w), 634 (w), 584 (w), 553 (w), 456 (w). UV/Vis (Methanol): λ_{max} = 292, 250, 217 nm. HR-ESI-MS m/z: $C_{21}H_{22}N_2O_2$ calcd: 357.1574 [M+Na$^+$], found: 357.1574.

(*E*)-*N*'-((6-(*tert*-butyl)-4-oxo-4*H*-chromen-3-yl)methylene)benzenesulfonohydrazide (**23**)

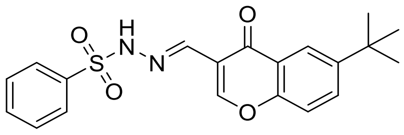

A solution of 137.8 mg (0.8 mmol) benzene sulfonylhydrazide in 1.6 mL ethanol and 0.4 mL water was added dropwise to a solution of 184.2 mg (0.8 mmol) **8a** in 0.8 mL chloroform. The reaction was stirred for 1 h at room temperature. The precipitate formed was filtered off and recrystallized from ethanol. Yield: 0.163 g (0.42 mmol, 53 %). m.p.: 183 °C (decomposition). ^1H NMR (400 MHz, [D_6]DMSO) δ = 1.29 (s, 9H), 7.55 – 7.70 (m, 4H), 7.84 – 7.92 (m, 3H), 7.96 (d, *J* = 2.5 Hz, 1H), 8.01 (d, *J* = 0.7 Hz, 1H), 8.58 (s, 1H), 11.56 (s, 1H). ^{13}C NMR (101 MHz, [D_6]DMSO) δ = 30.9, 34.6, 117.6, 118.4, 120.2, 122.6, 127.2, 129.3, 132.6, 133.1, 138.9, 139.9, 148.7, 153.9, 154.2, 174.8. IR (KBr): $\tilde{\nu}$ = 3447 (m), 3155 (m), 3085 (w), 2963 (m), 2907 (w), 2871 (w), 1633 (s), 1619 (s), 1572 (m), 1484 (s), 1465 (m), 1447 (m), 1382 (m), 1366 (m), 1338 (m), 1316 (m), 1265 (m), 1226 (w), 1207 (w), 1170 (s), 1125 (w), 1089 (m), 1069 (m), 1026 (w), 1002 (w), 931 (m), 882 (w), 825 (m), 797 (m), 781 (w), 756 (m), 720 (m), 688 (m), 666 (w), 586 (s), 573 (m), 458 (w). UV/Vis (Methanol): λ_{max} = 301, 265, 218, 202 nm. HR-ESI-MS m/z: $C_{20}H_{20}N_2O_4S$ calcd: 385.1217 [M+H$^+$], found: 385.1217.

Supplementary Methods

X-ray structure determination

The single crystal X-ray diffraction data of compound **8** were collected on a StadiVari diffractometer (Stoe & Cie GmbH, Darmstadt, Germany) using Cu-Kα radiation (λ = 1.54186 Å) of an X-ray microsource. The structure was solved by direct methods using SHELXS and refined using SHELXL-2014/7.[8] All non-hydrogen atoms were refined anisotropically. Hydrogen atoms at C10, N2 and at the O atom of the co-crystallizing water molecule were localized and refined, all other hydrogen atoms were included in idealized positions.

Crystal data of $C_{20}H_{19}N_3O_3 \cdot H_2O$: M_m = 367.40 g mol^{-1}, colorless plate, crystal size 0.035 × 0.10 × 0.16 mm^3, triclinic, space group $P\bar{1}$ (no. 2), *z* = 2, *a* = 6.6257(2), *b* = 9.2862(2), *c* = 15.5005(3) Å, α = 85.577(2), β = 82.165(2), γ = 73.604(2) °, *V* = 905.63(4) Å^3, T = 180(2) K, d_{calc} = 1.347 g cm^{-3}, μ = 0.783 mm^{-1}, 37152 measured, 3354 independent reflections, R_{int} = 0.018, 2980 observed reflections with I > 2σ(I), 264 parameters; R1 (observed reflections) = 0.0352, wR2 (all data) = 0.1016; max. and min. residual electron density peaks 0.39 and −0.26 e−/10^6 pm^3.

CCDC 1557393 contains the supplementary crystallographic data for **8**. These data can be obtained free of charge from the Cambridge Crystallographic Data Centre via www.ccdc.cam.ac.uk/data_request/cif.

Cell culture

HepG2 cells were maintained in MEM (Modified Eagle's Medium, Invitrogen) containing 10% FBS (Life Technologies), 2 mM L-glutamine (PAA Laboratories), 0.1 mM non-essential amino acids (Invitrogen), 1 mM sodium pyruvate (Invitrogen), 1.5 g/L sodium bicarbonate (PAA Laboratories), 100 µg/mL penicillin/streptomycin (PAA Laboratories) at 37 ºC, 5% CO_2 and 95% humidity. MDA-MB-453 and MDA-MB-231 cells were cultured in Leibovitz's L-15 medium (Gibco Life Technologies) supplemented with 10 % (v/v) FBS (Gibco Life Technologies) and 1 % (v/v) penicillin/streptomycin (Gibco Life Technologies) at 37 °C and 95 % humidity with no additional CO_2. K562 cells were cultured in RPMI 1640 medium (Gibco Life Technologies), containing 10% FBS (Gibco Life Technologies), 2 mM L-glutamine (Gibco Life Technologies) and penicillin/streptomycin (Gibco Life Technologies), at 37 °C, 5 % CO_2 and 95 % humidity. Parental and Erlotinib-resistant HCC-827 cells were cultured in RPMI 1640 medium (Gibco Life Technologies), containing 10 % (v/v) FBS (Gibco Life Technologies), 2 mM L-glutamine (Gibco Life Technologies), 1 mM sodium pyruvate (Gibco Life Technologies) and 1 % (v/v)

penicillin/streptomycin (Gibco Life Technologies) at 37 °C, 5 % CO_2 and 95 % humidity. HCC-827 cells with acquired resistance against Erlotinib were obtained by treating parental HCC-827 cells with subtoxic concentrations of Erlotinib over a period of several months. Concentrations of Erlotinib were increased in the course of the incubation, until the cells could be maintained in culture at an Erlotinib concentration of 10 µM.

Supplementary references

1. Becker, S., Groner, B. & Muller, C. W. Three-dimensional structure of the Stat3beta homodimer bound to DNA. *Nature* **394**, 145-151, (1998).
2. Itoh, H. *et al.* 2-Acyl-6-aminomethylphenol derivatives. *United States Patent 4245099*, (1981).
3. Du, Z. T., Lu, J., Yu, H. R., Xu, Y. & Li, A. P. A facile demethylation of ortho substituted aryl methyl ethers promoted by AlCl3. *J Chem Res*, 222-227, (2010).
4. Harnisch, H. Chromone-3-Carbaldehydes. *Liebigs Ann Chem* **765**, 8-14, (1972).
5. Nohara, A., Umetani, T. & Sanno, Y. Studies on Antianaphylactic Agents .1. Facile Synthesis of 4-Oxo-4h-1-Benzopyran-3-Carboxaldehydes by Vilsmeier Reagents. *Tetrahedron* **30**, 3553-3561, (1974).
6. Müller, J., Sperl, B., Reindl, W., Kiessling, A. & Berg, T. Discovery of chromone-based inhibitors of the transcription factor STAT5. *ChemBioChem* **9**, 723-727, (2008).
7. A. K. Baruah, D. Prajapati & Sandhu, J. S. Studies in Chromone Derivatives: Cycloaddition Reactions of 4-oxo-4H-1-Benzopyran-3-Carboxyaldehyde Imines with Benzonitrile oxide and Nitrilimine of 4-oxo-4H-1-Benzopyran-3-Carboxyaldehyde with alkenes. *Tetrahedron* **44**, 1241, (1988).
8. Sheldrick, G. M. A short history of SHELX. *Acta Crystallogr A* **64**, 112-122, (2008).

NMR spectra

^{1}H NMR of compound **3**

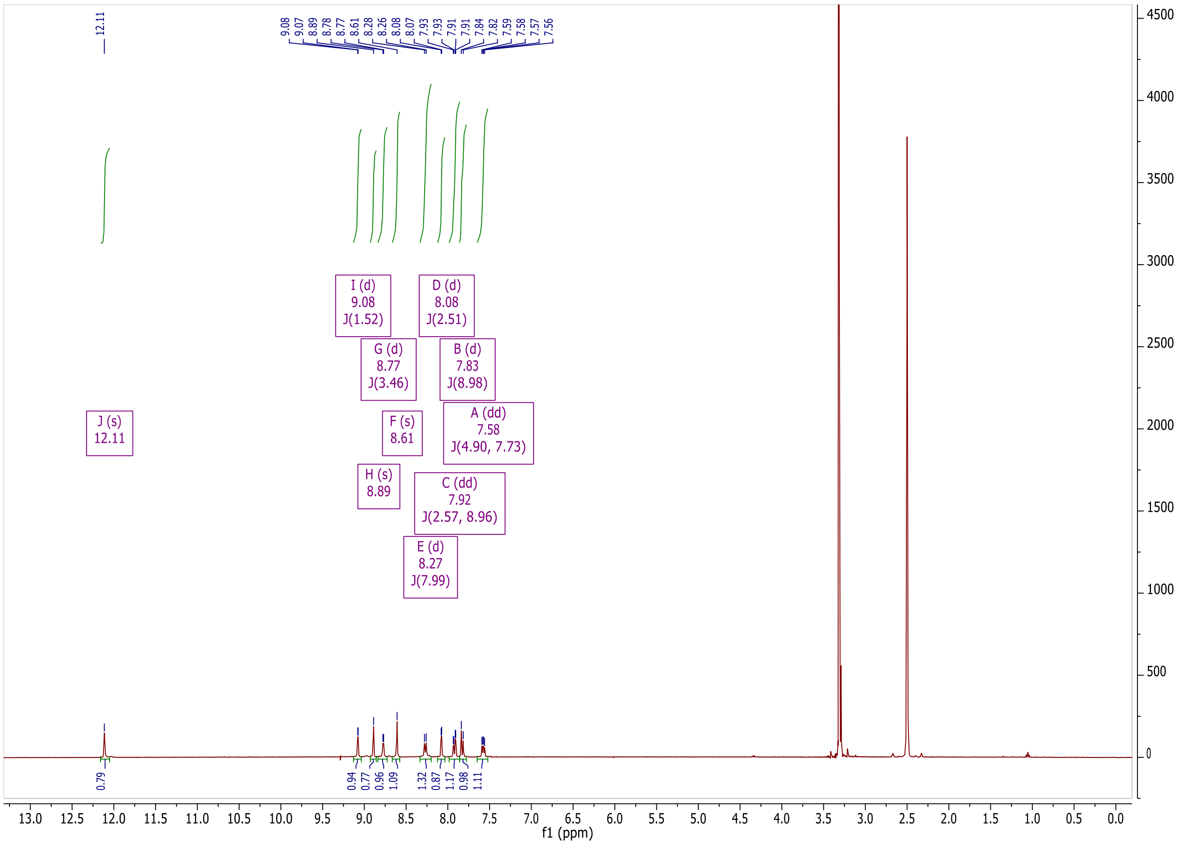

^{13}C NMR of compound **3**

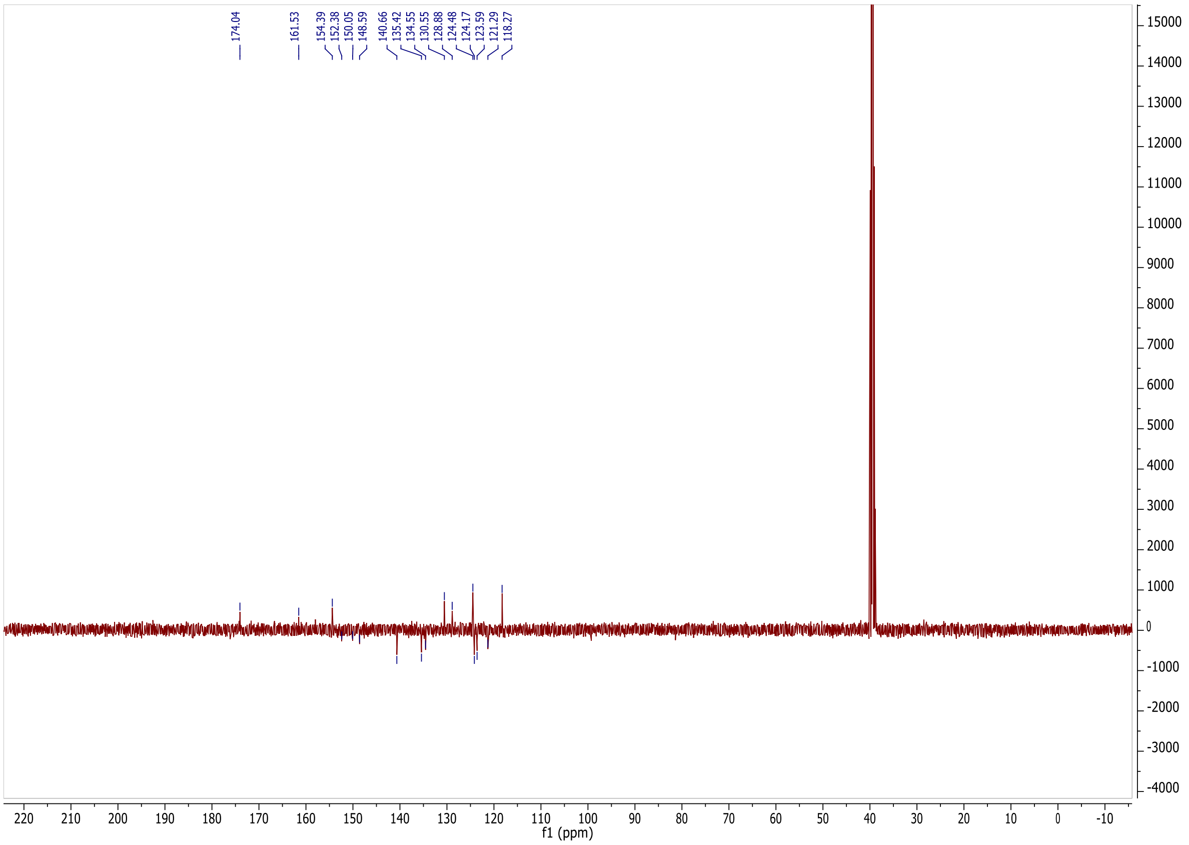

^{1}H NMR of compound **4**

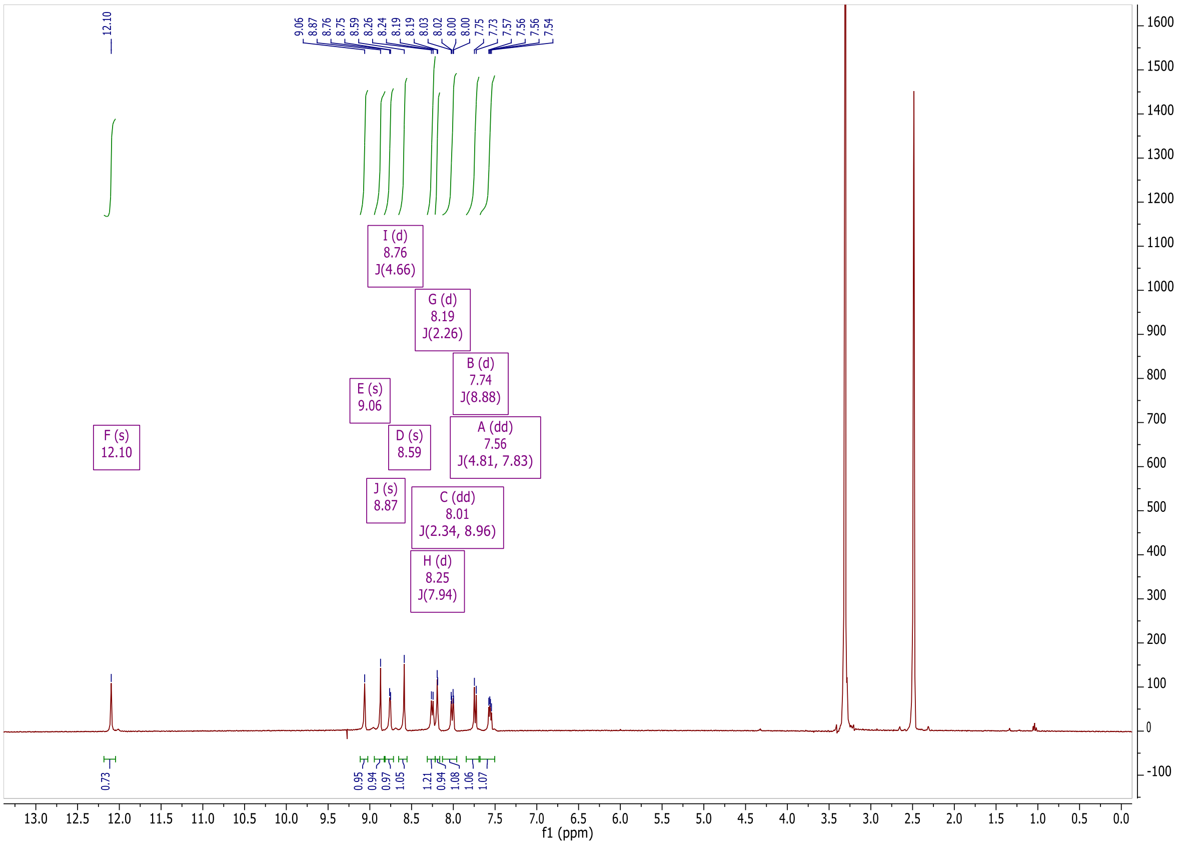

^{13}C NMR of compound **4**

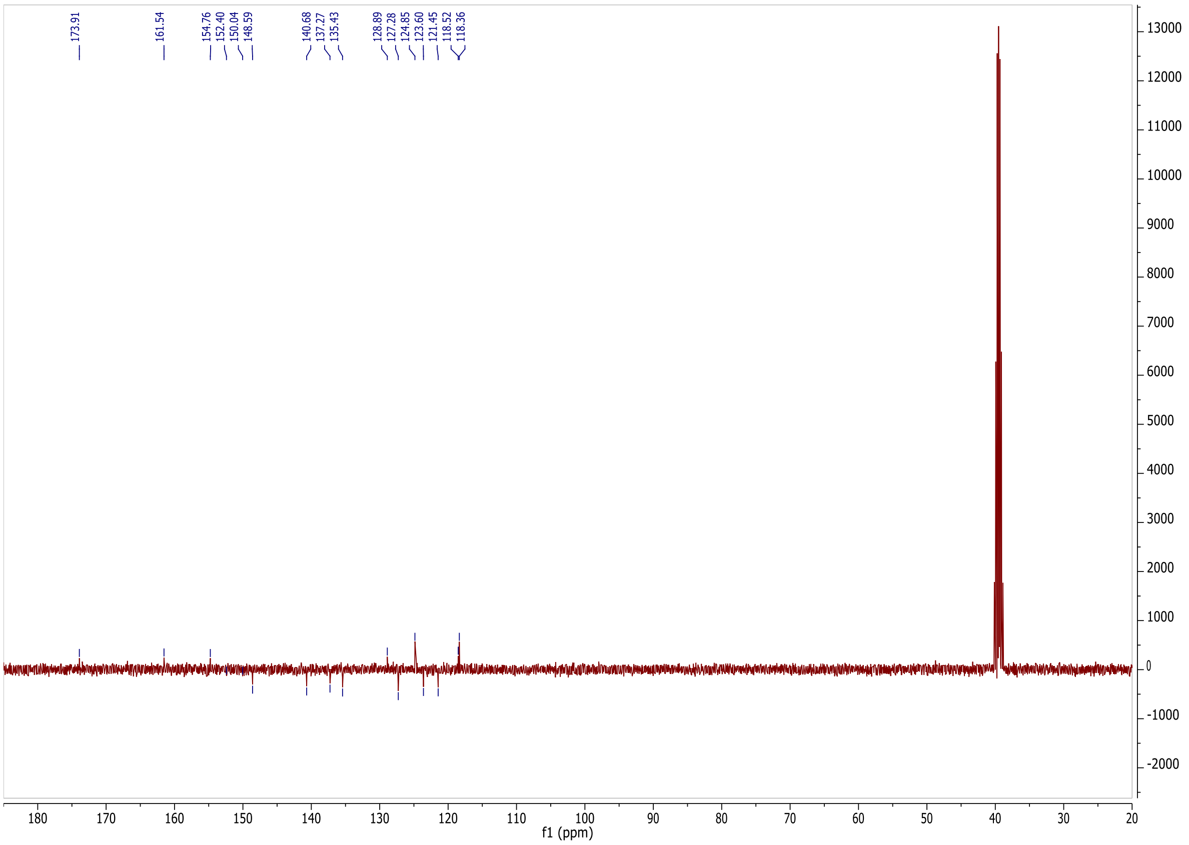

^{1}H NMR of compound **5**

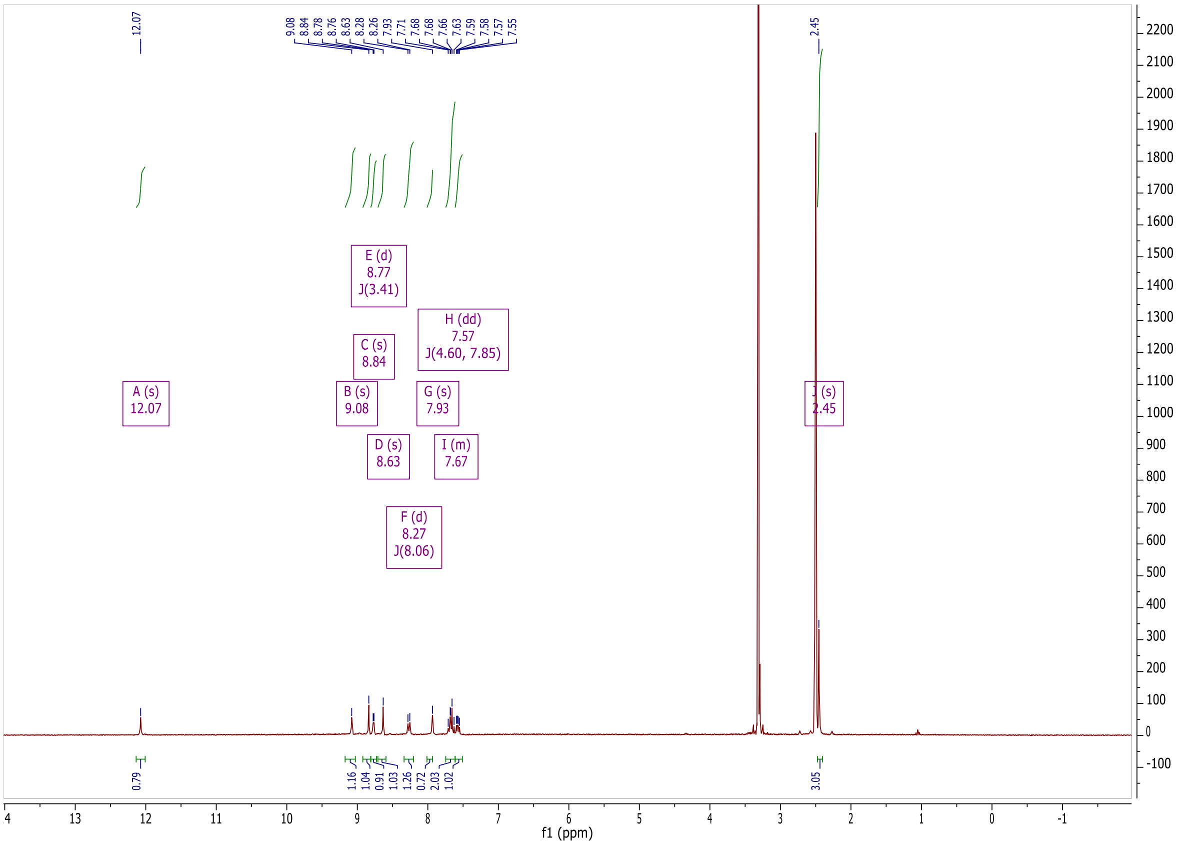

^{13}C NMR of compound **5**

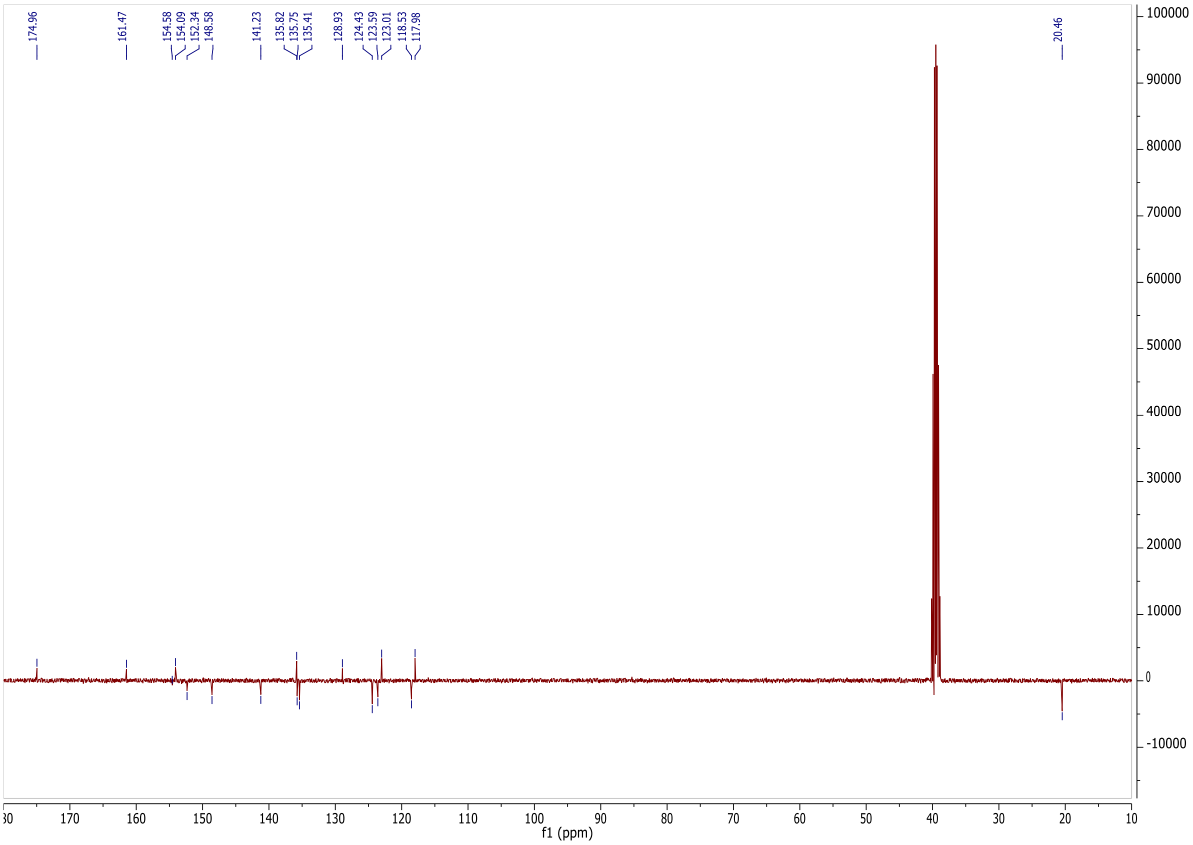

^{1}H NMR of compound **7**

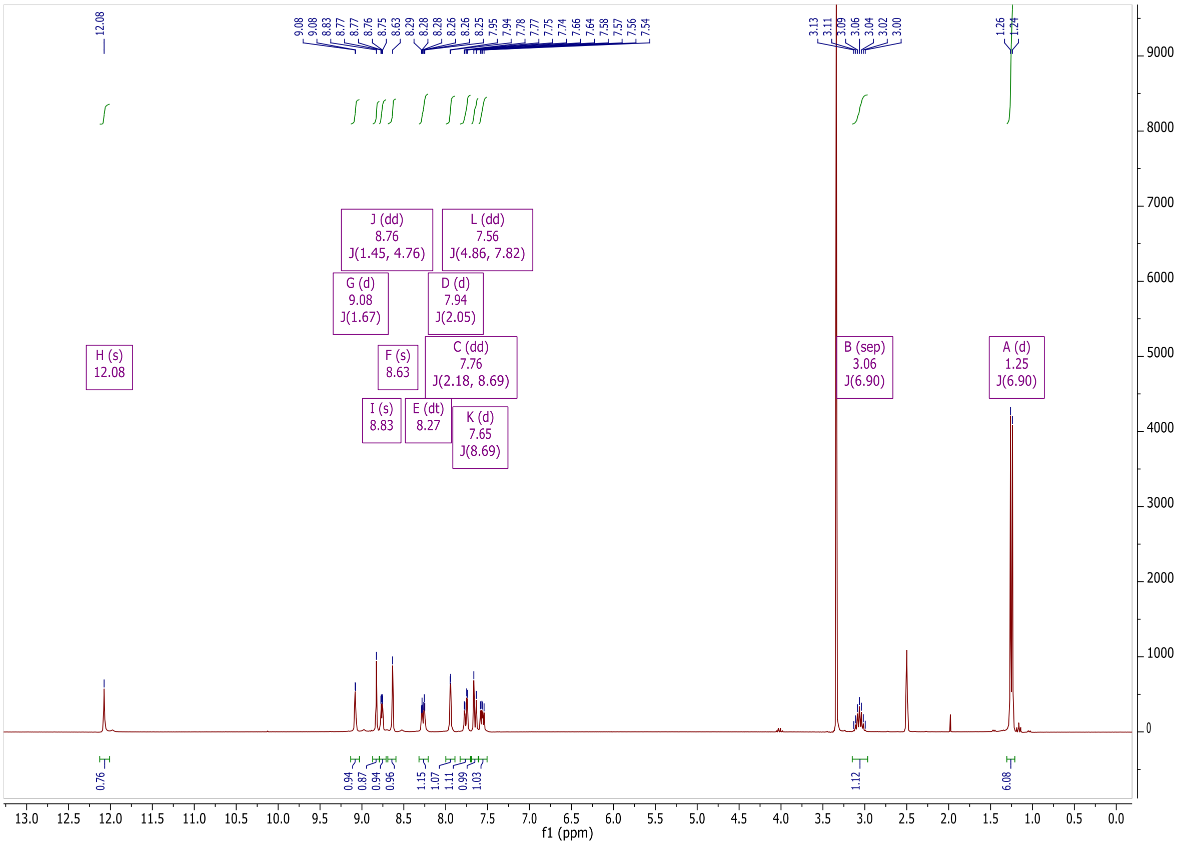

^{13}C NMR of compound **7**

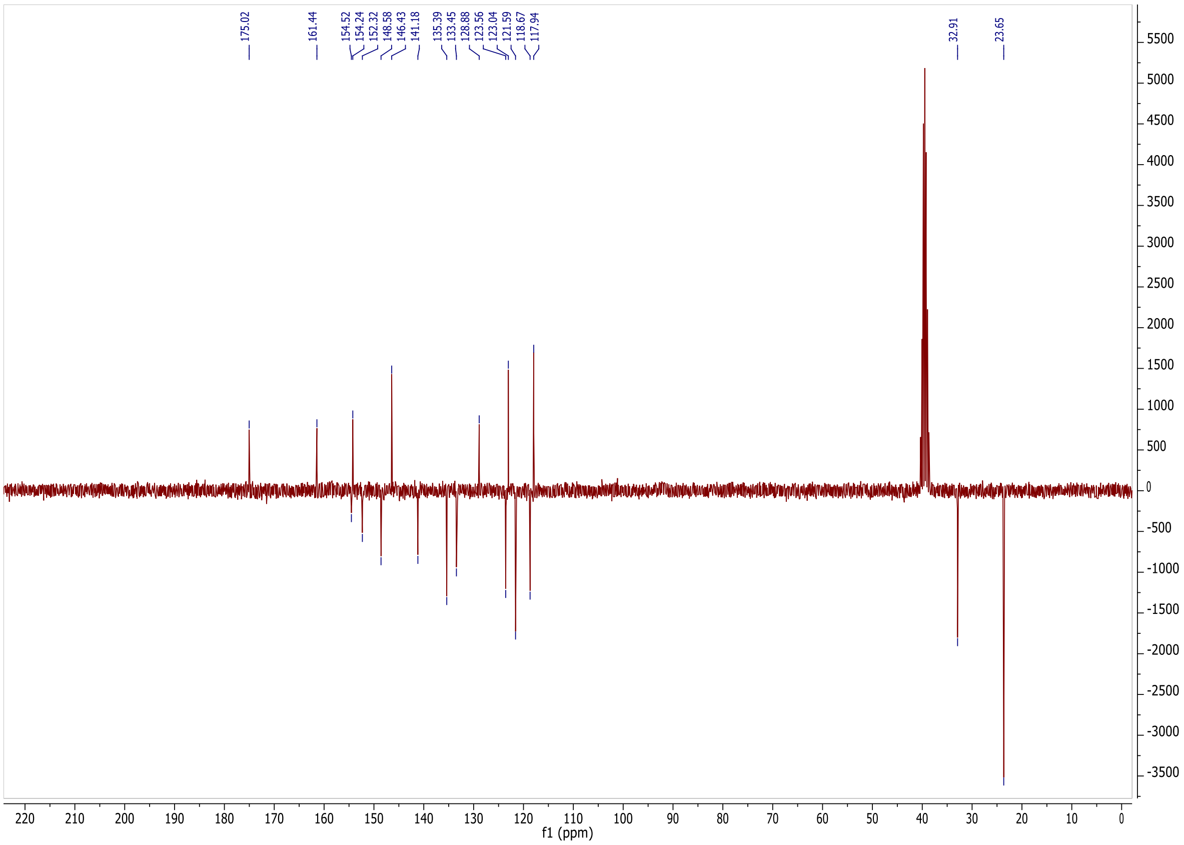

^{1}H NMR of compound **8**

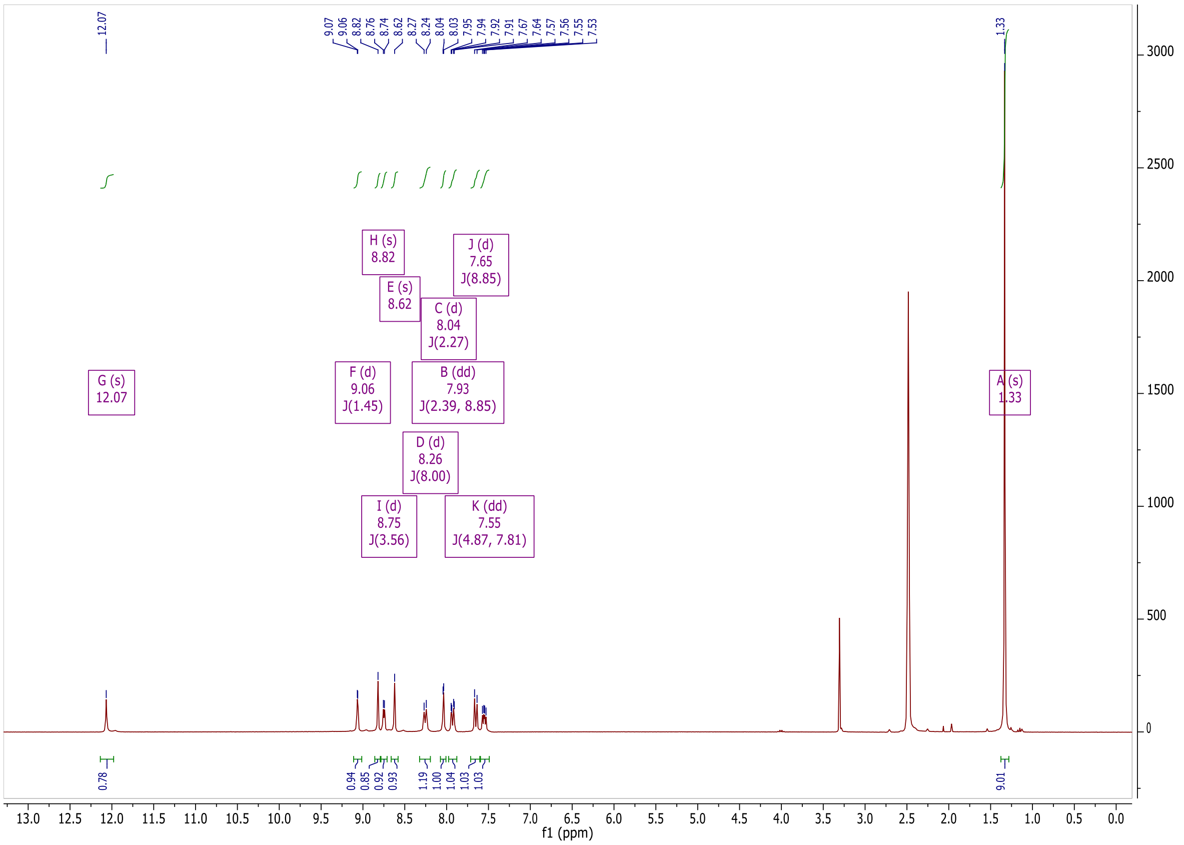

^{13}C NMR of compound **8**

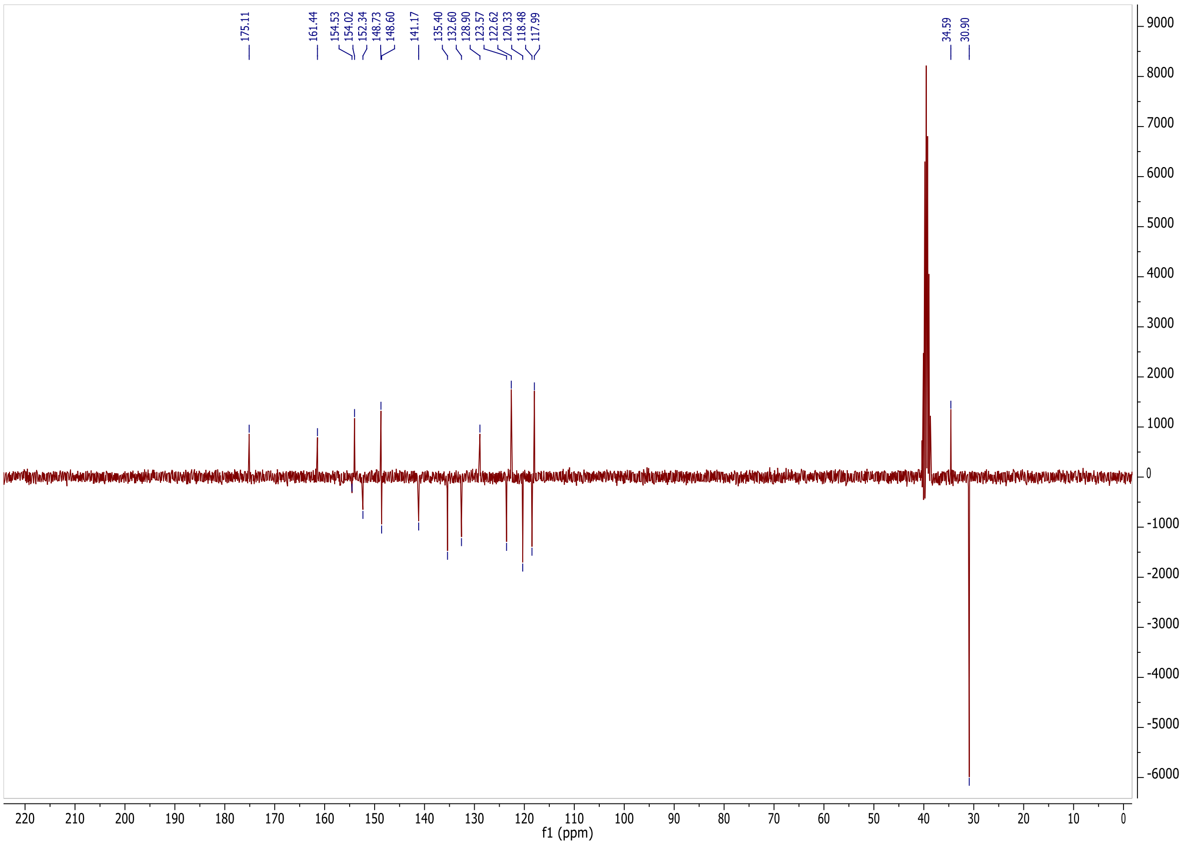

^{1}H NMR of compound **9**

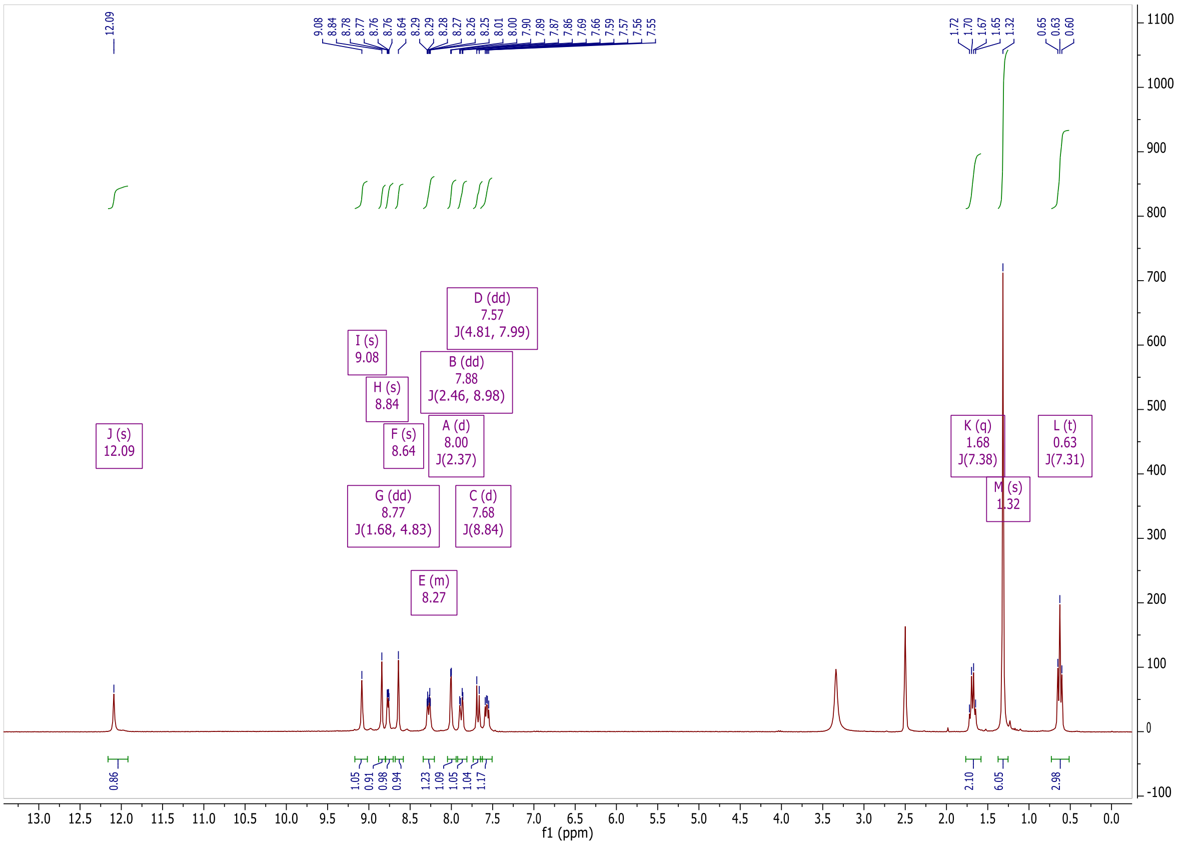

^{13}C NMR of compound **9**

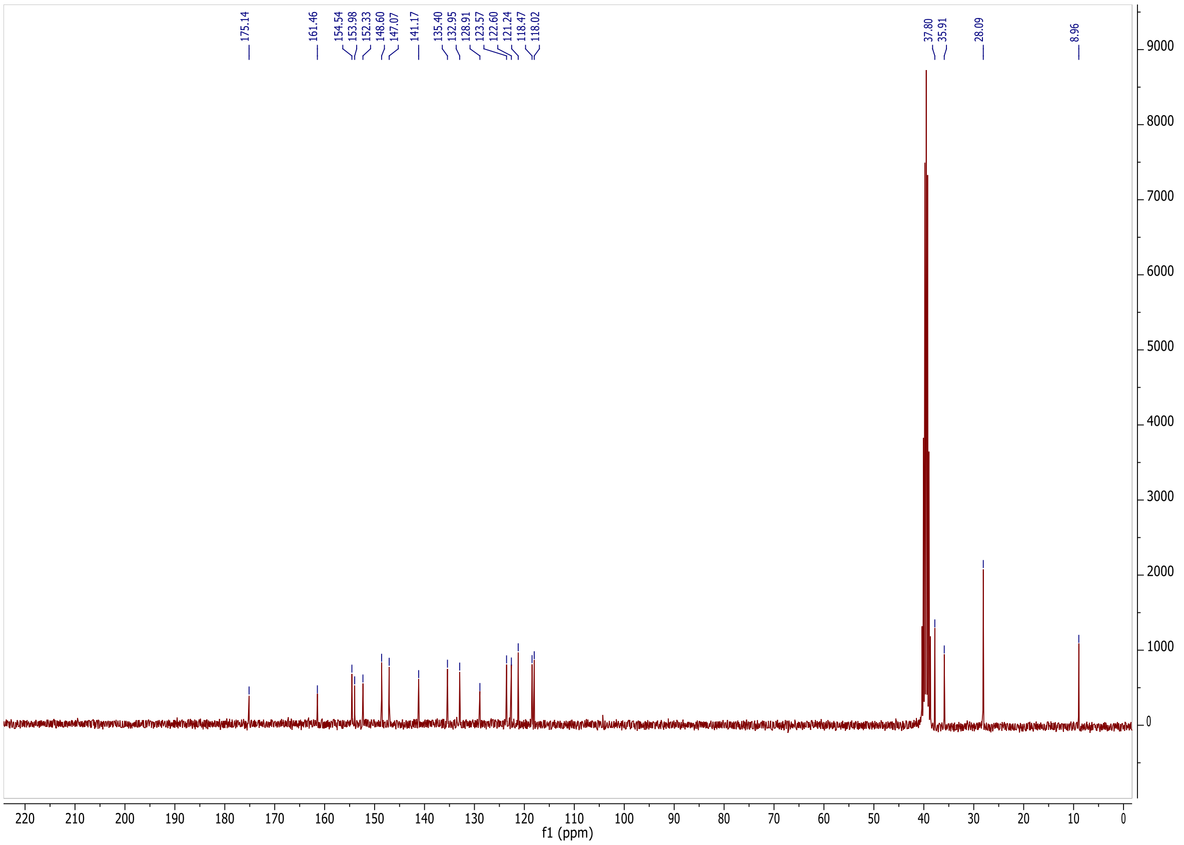

^{1}H NMR of compound **10**

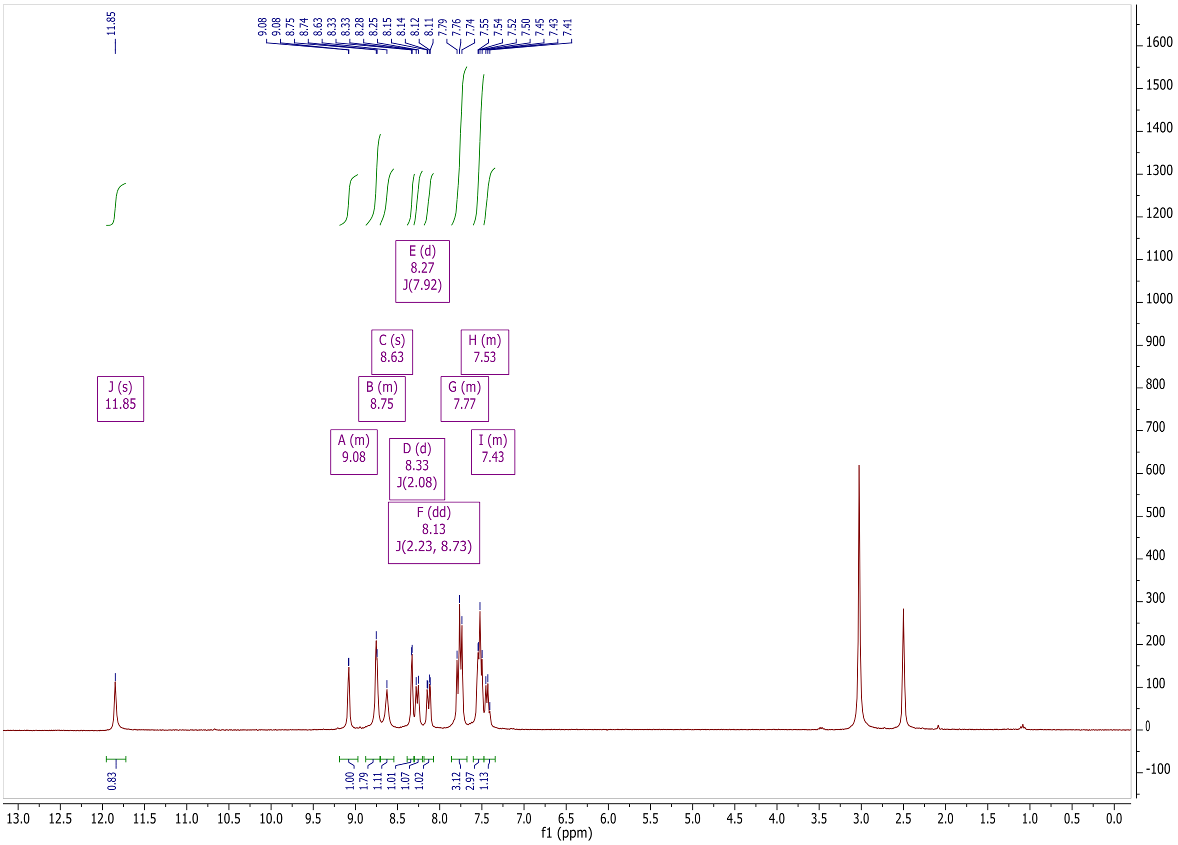

^{13}C NMR of compound **10**

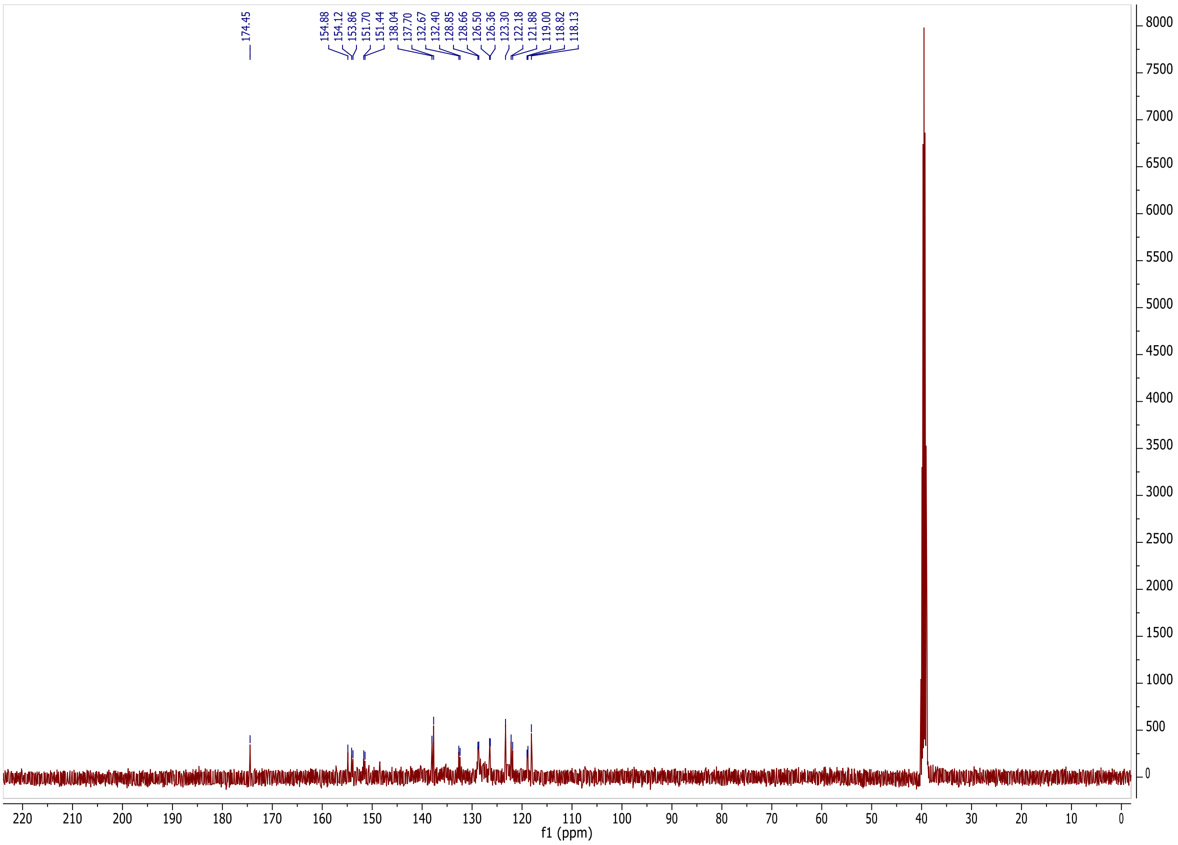

^1H NMR of compound **11**

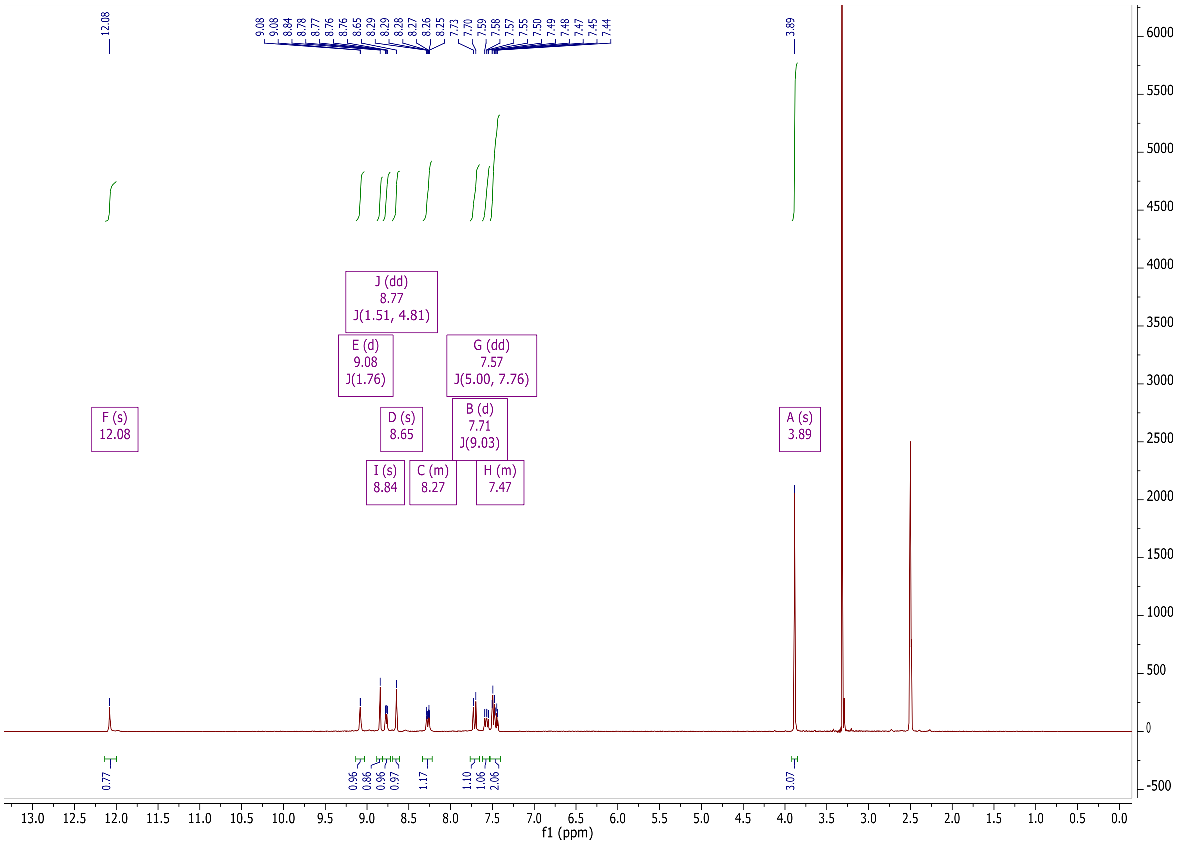

^{13}C NMR of compound **11**

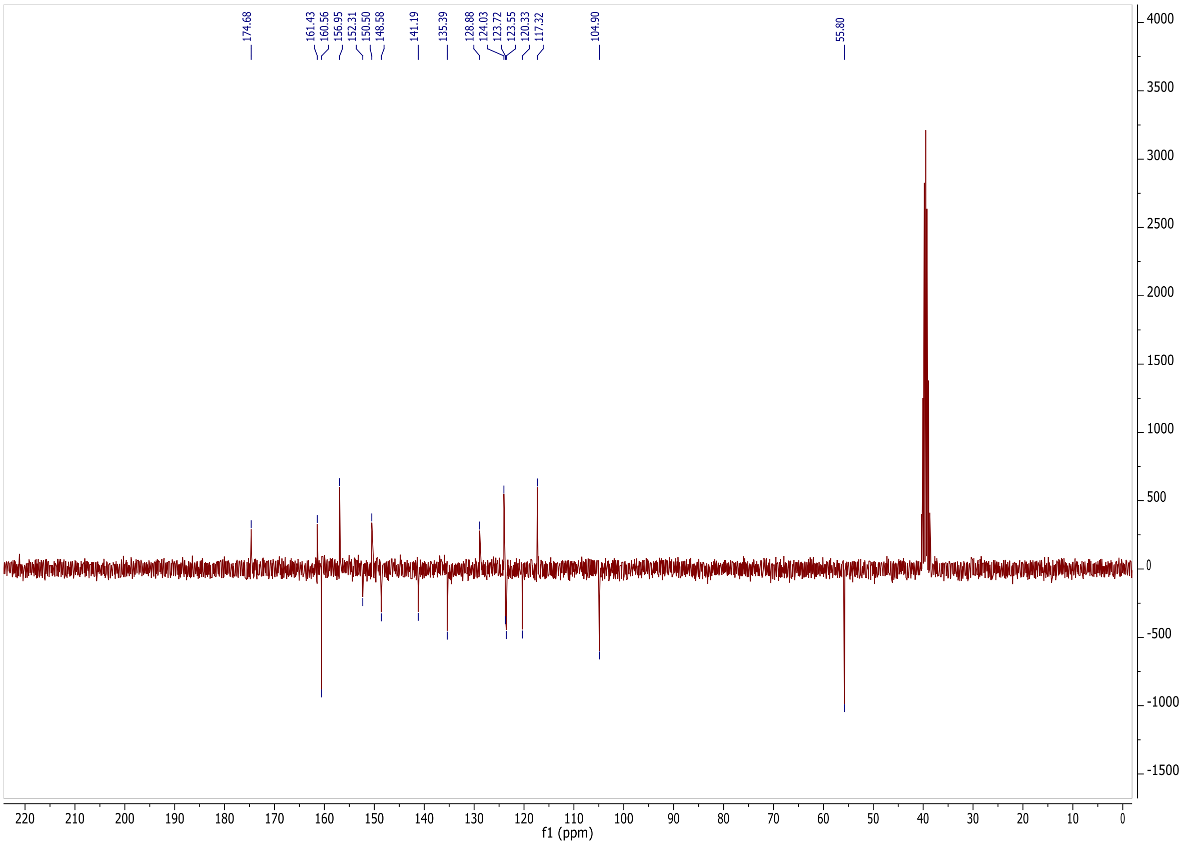

^{1}H NMR of compound **12**

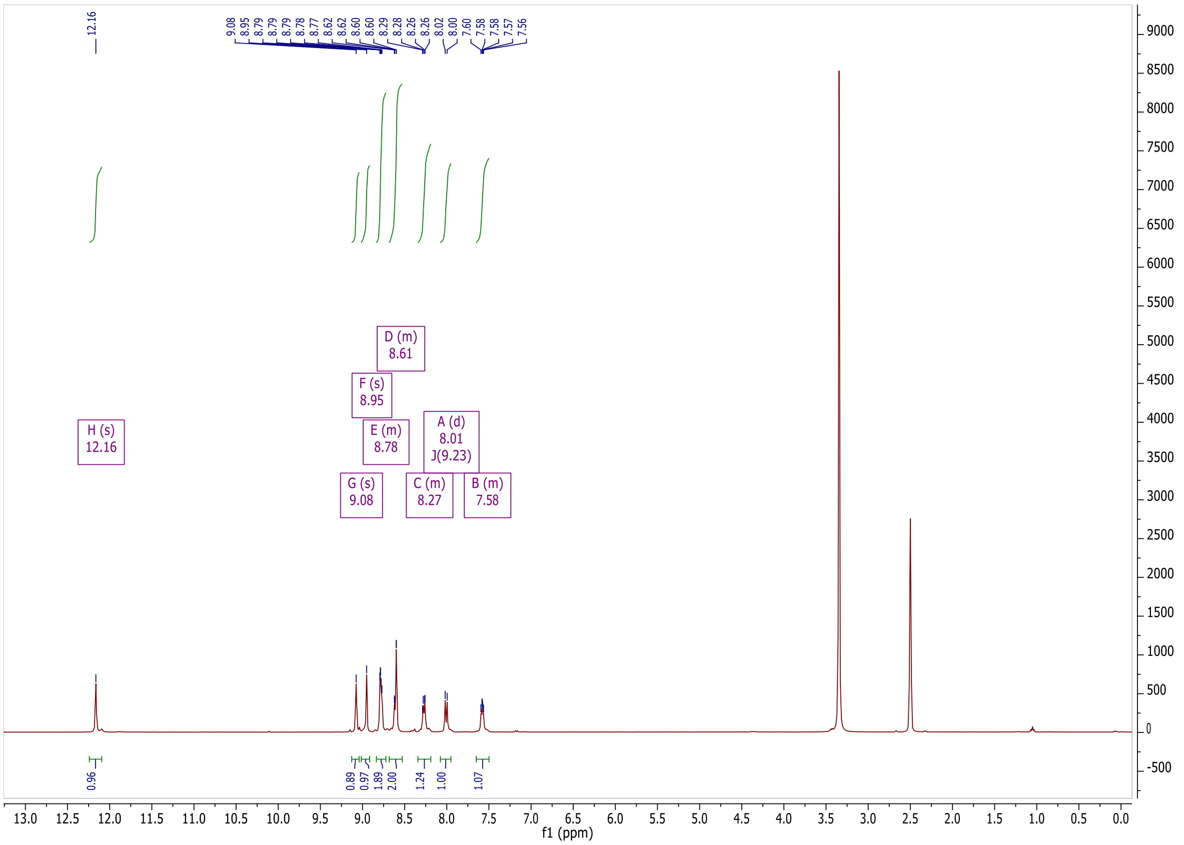

^{13}C NMR of compound **12**

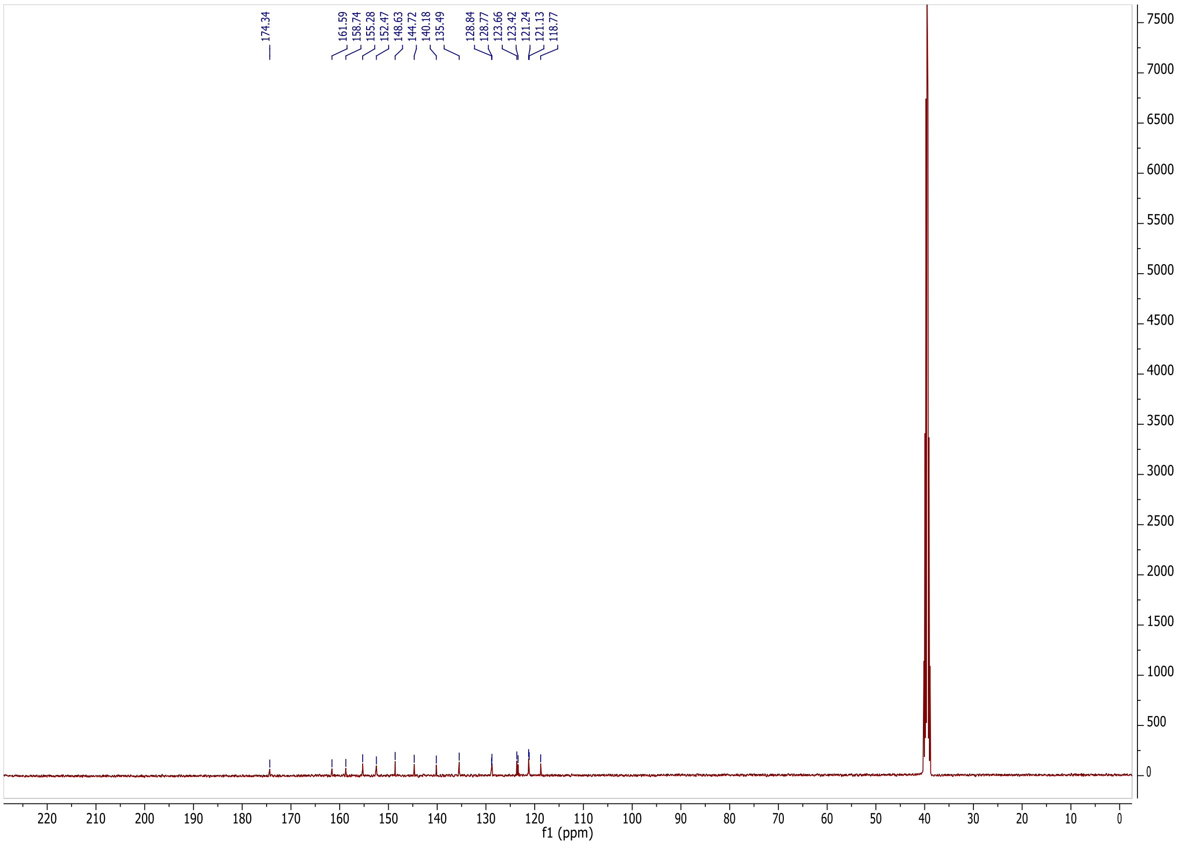

^{1}H NMR of compound **13**

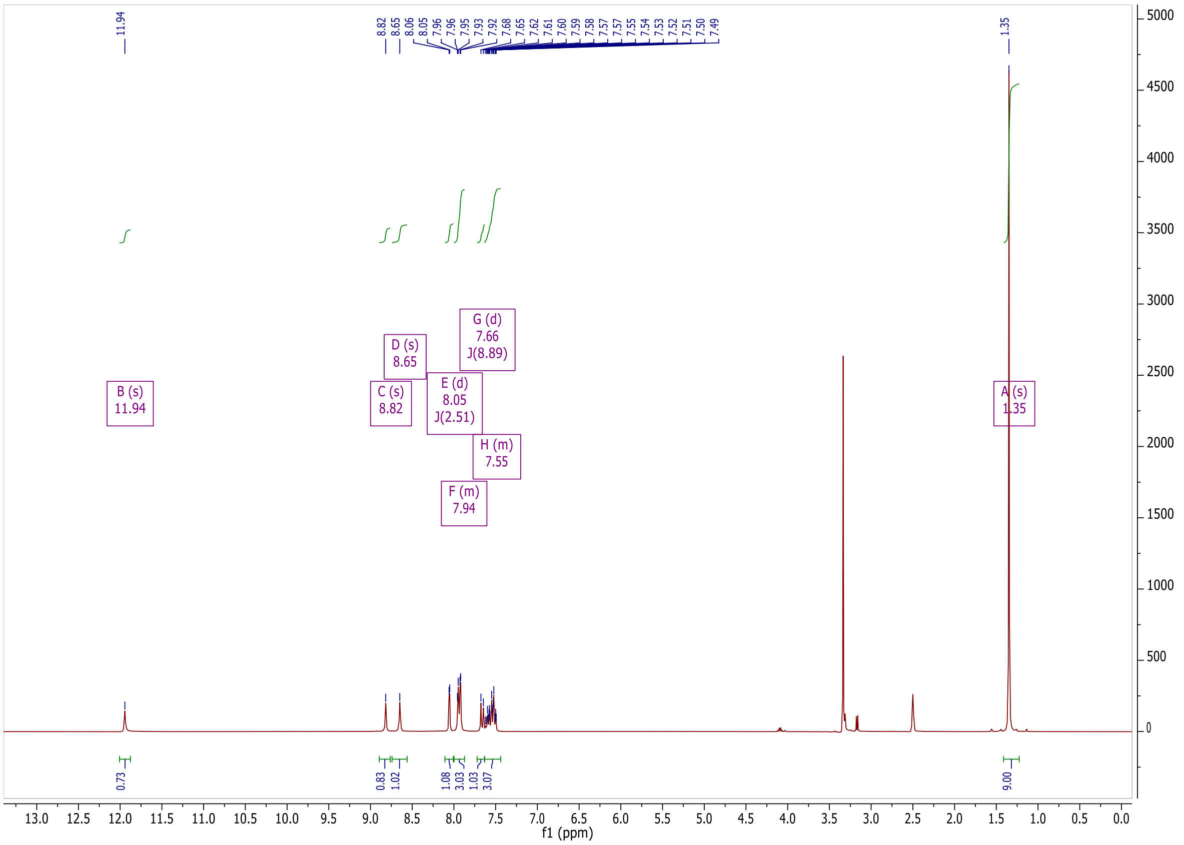

^{13}C NMR of compound **13**

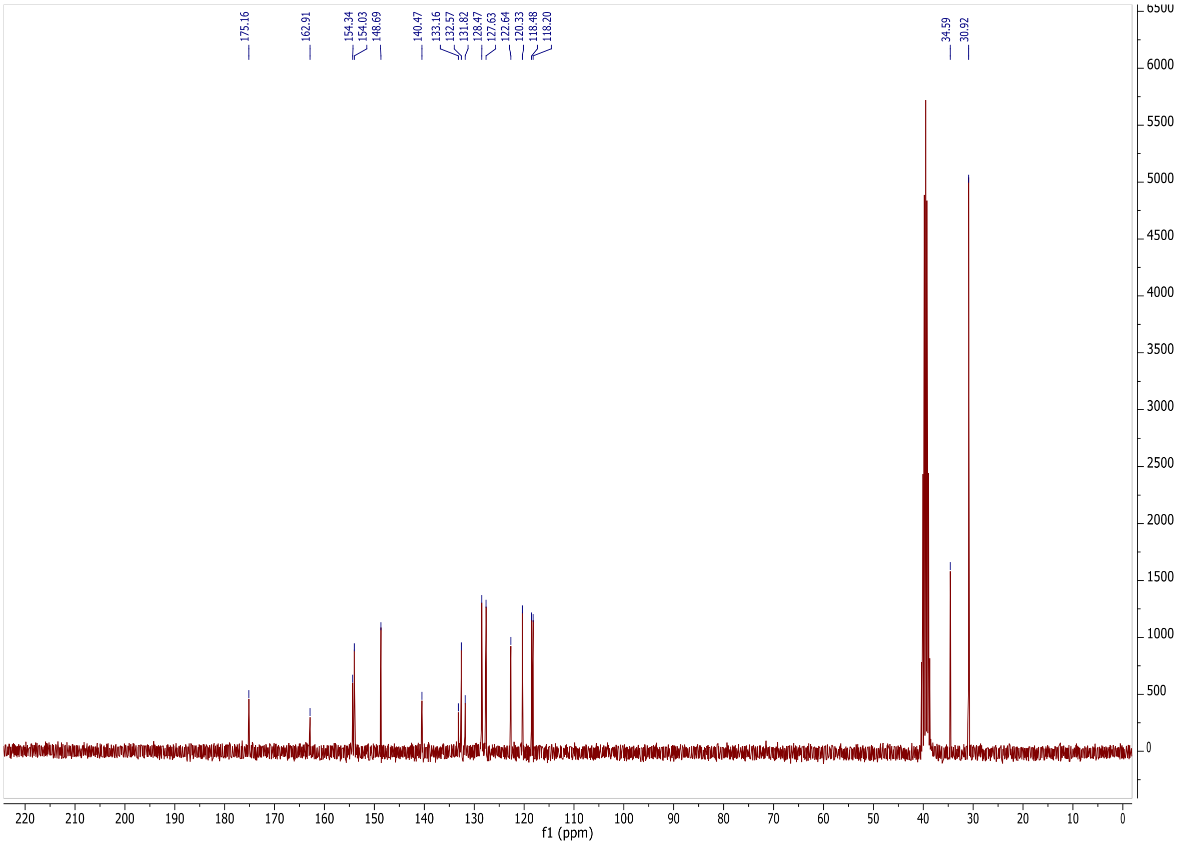

^{1}H NMR of compound **14**

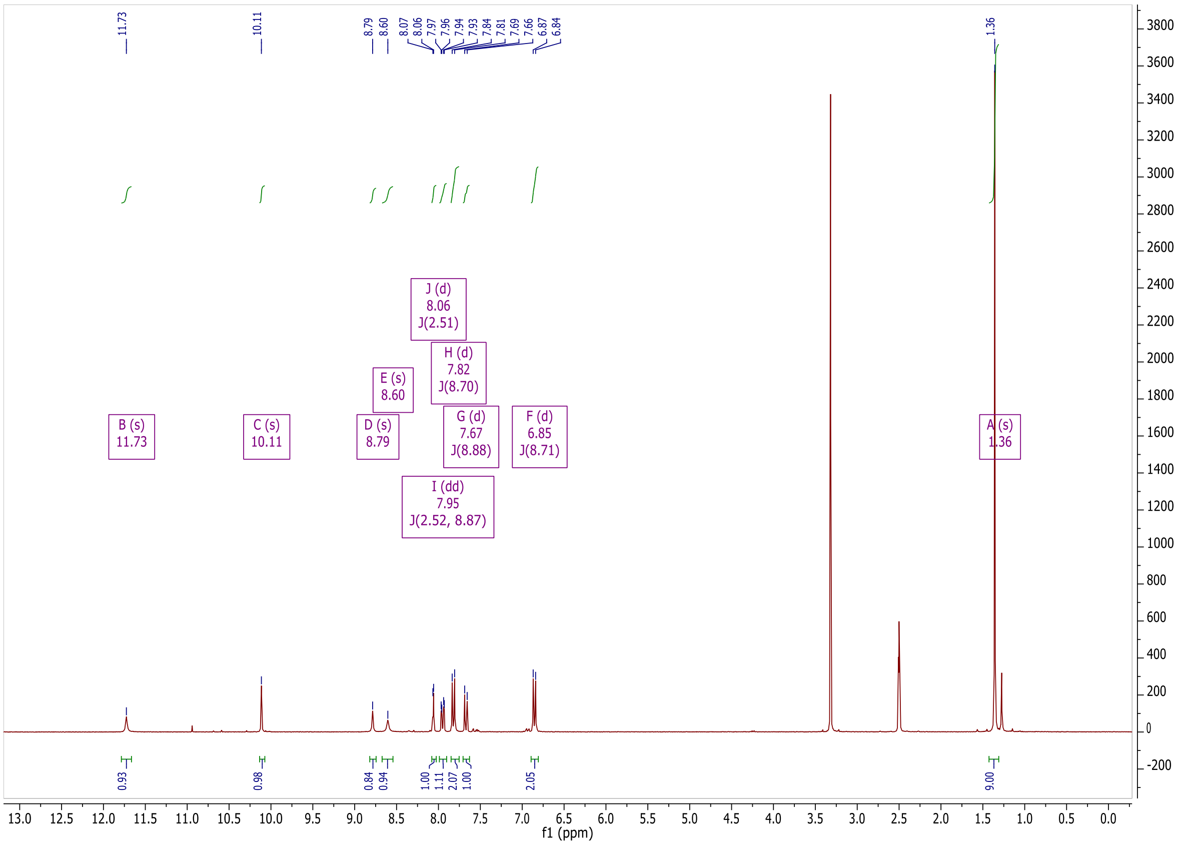

^{13}C NMR of compound **14**

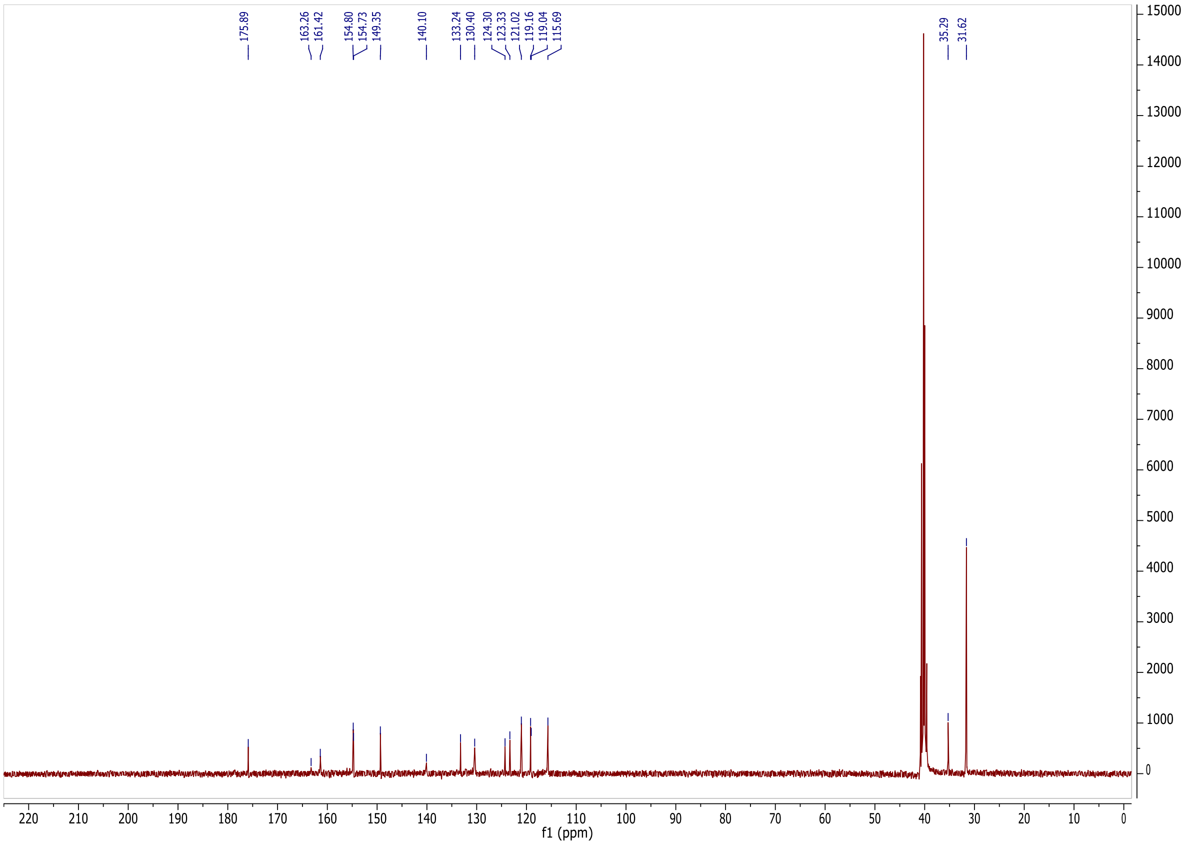

^{1}H NMR of compound **15**

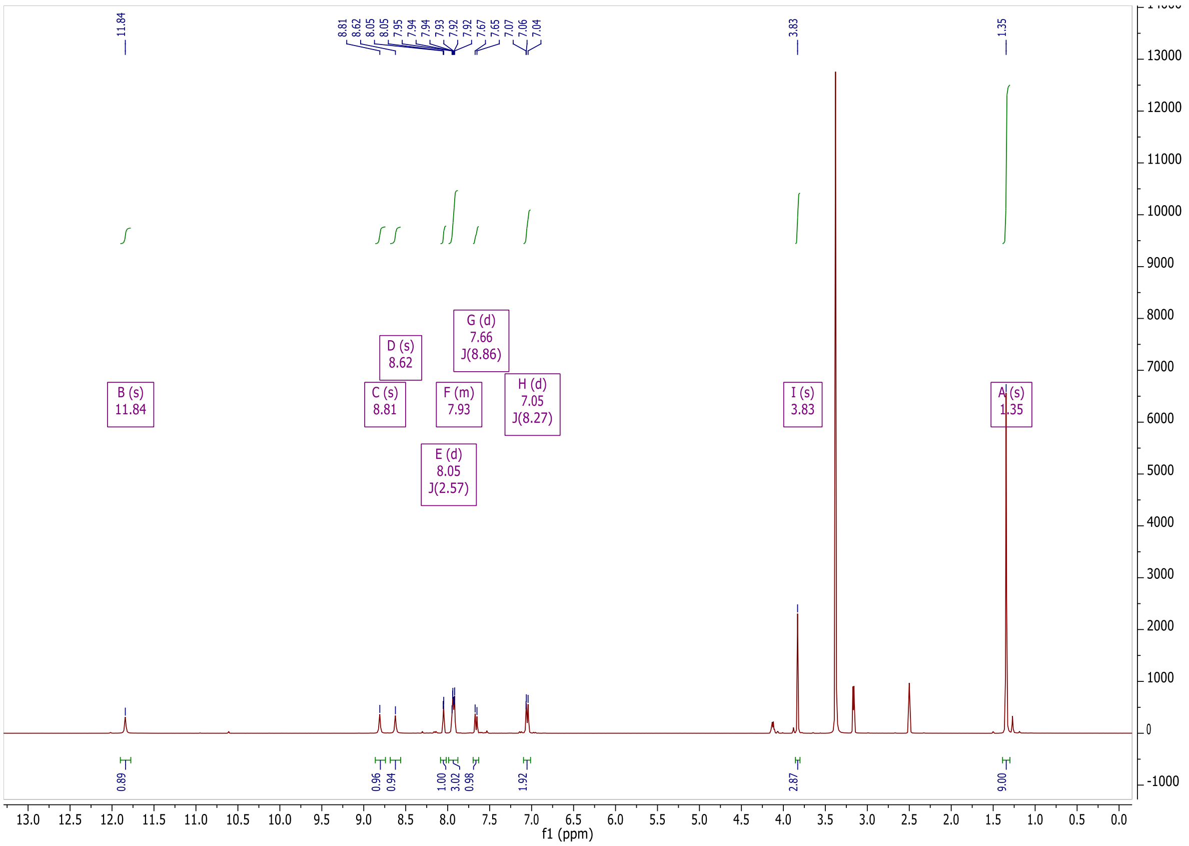

^{13}C NMR of compound **15**

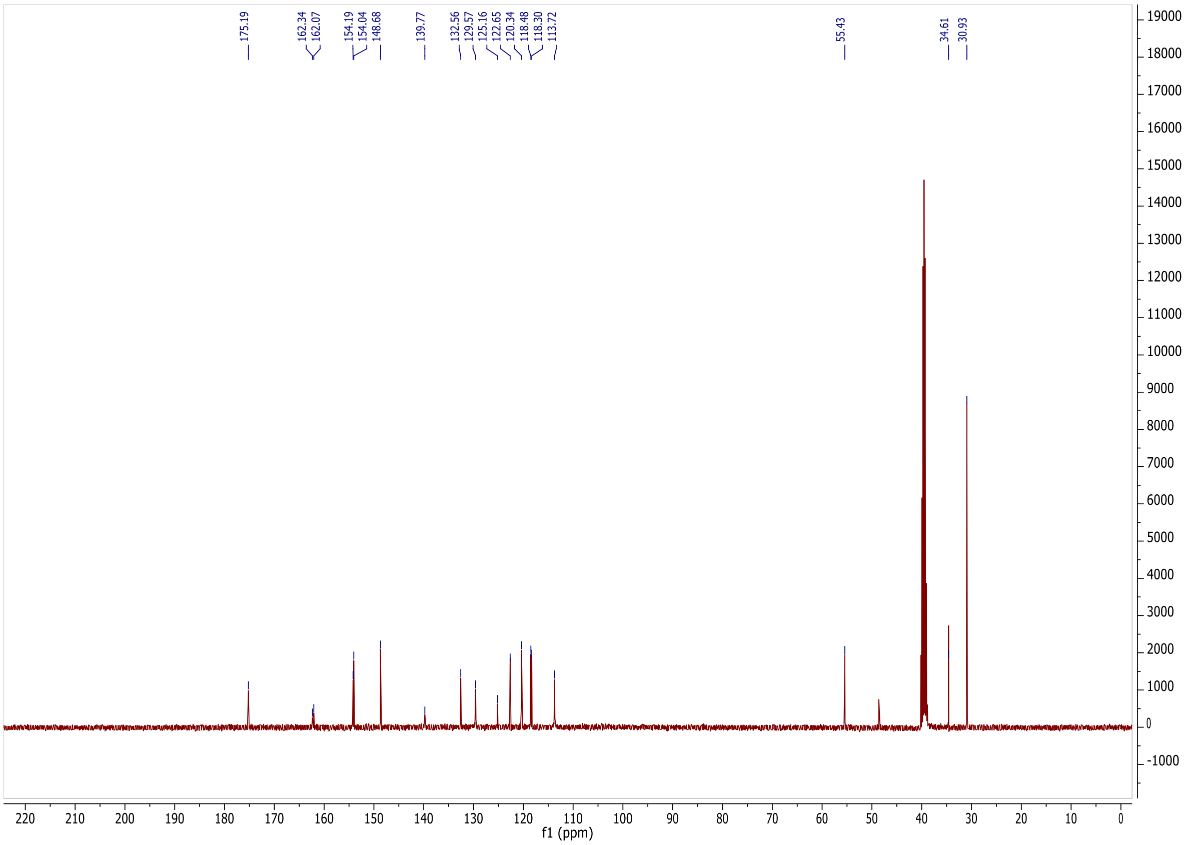

^{1}H NMR of compound **16**

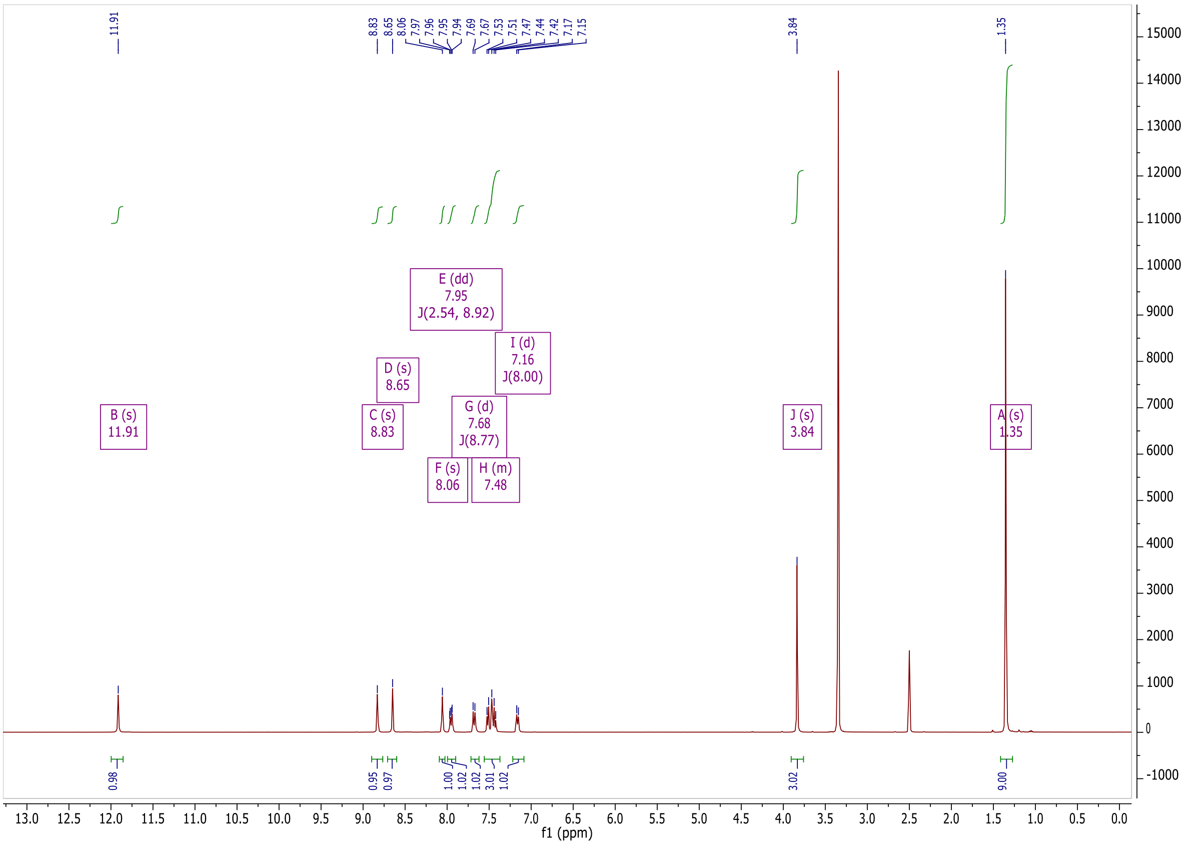

^{13}C NMR of compound **16**

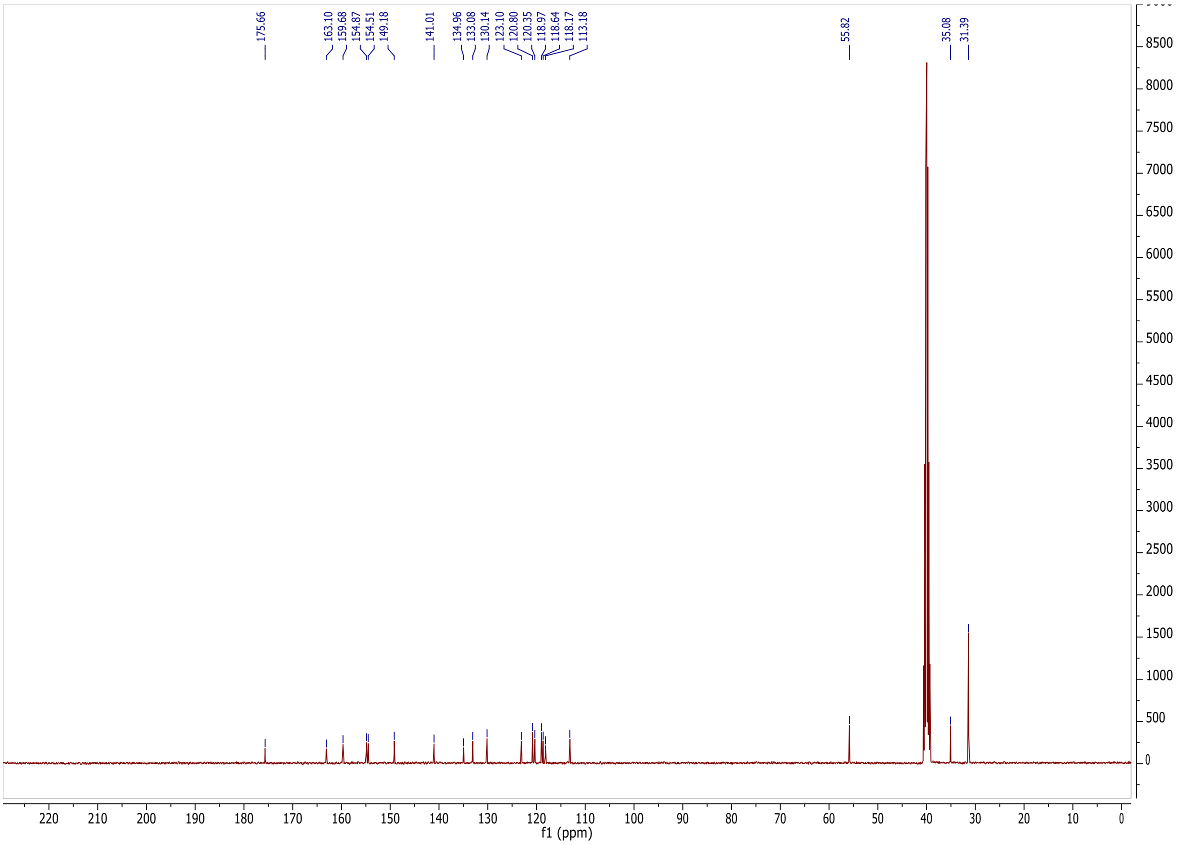

^{1}H NMR of compound **17**

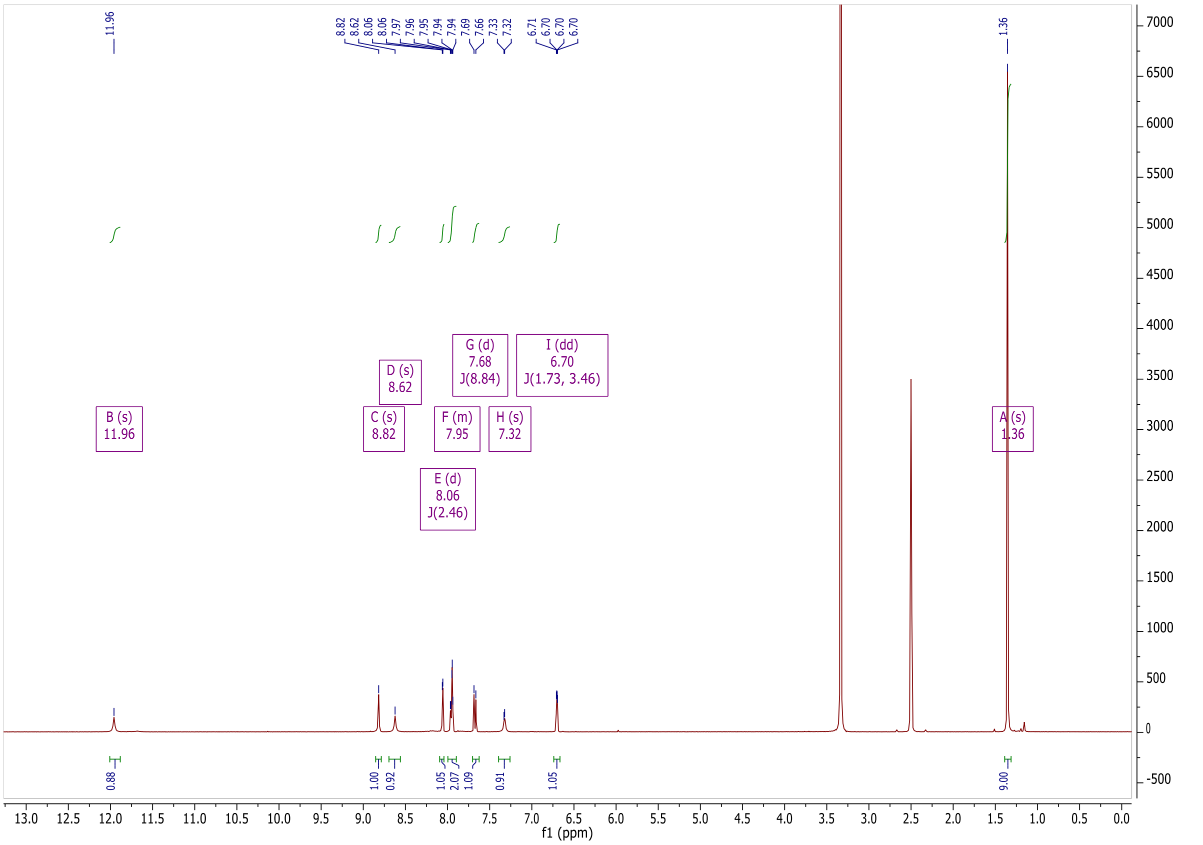

^{13}C NMR of compound **17**

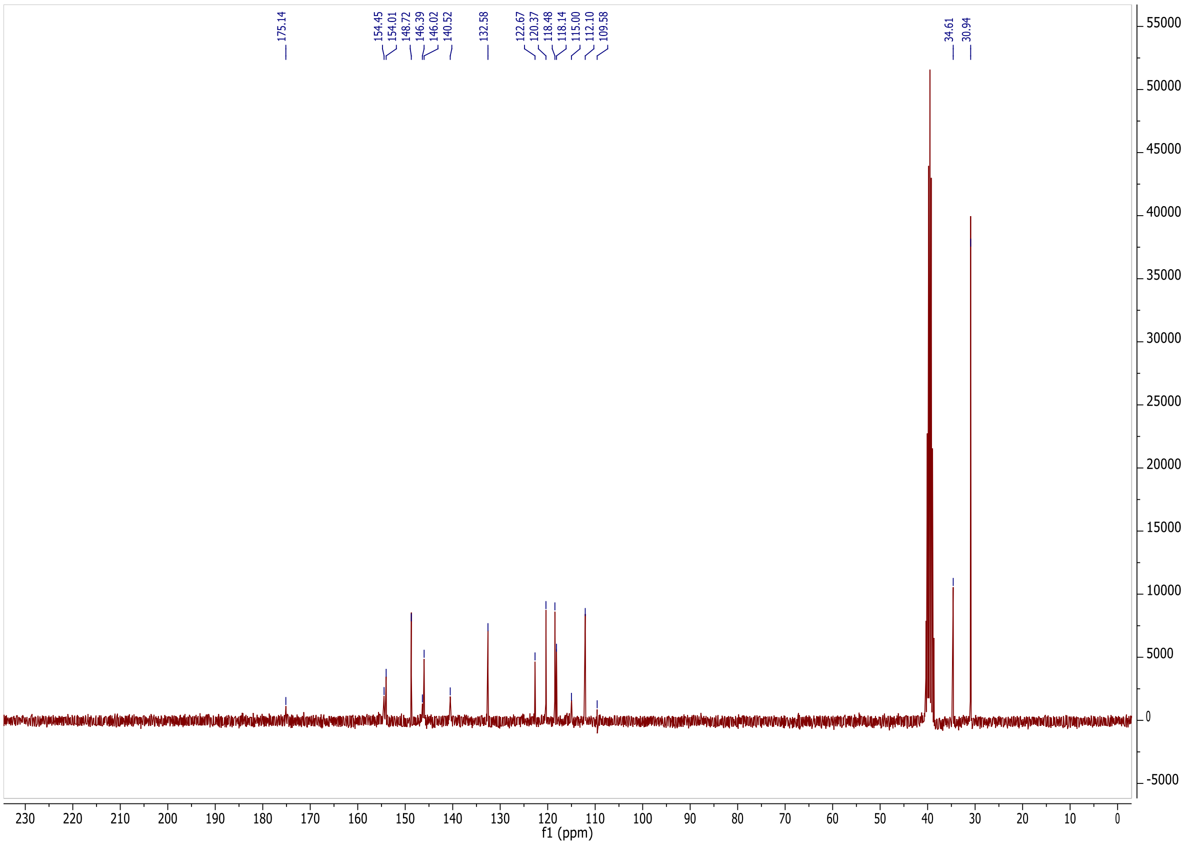

^{1}H NMR of compound **18**

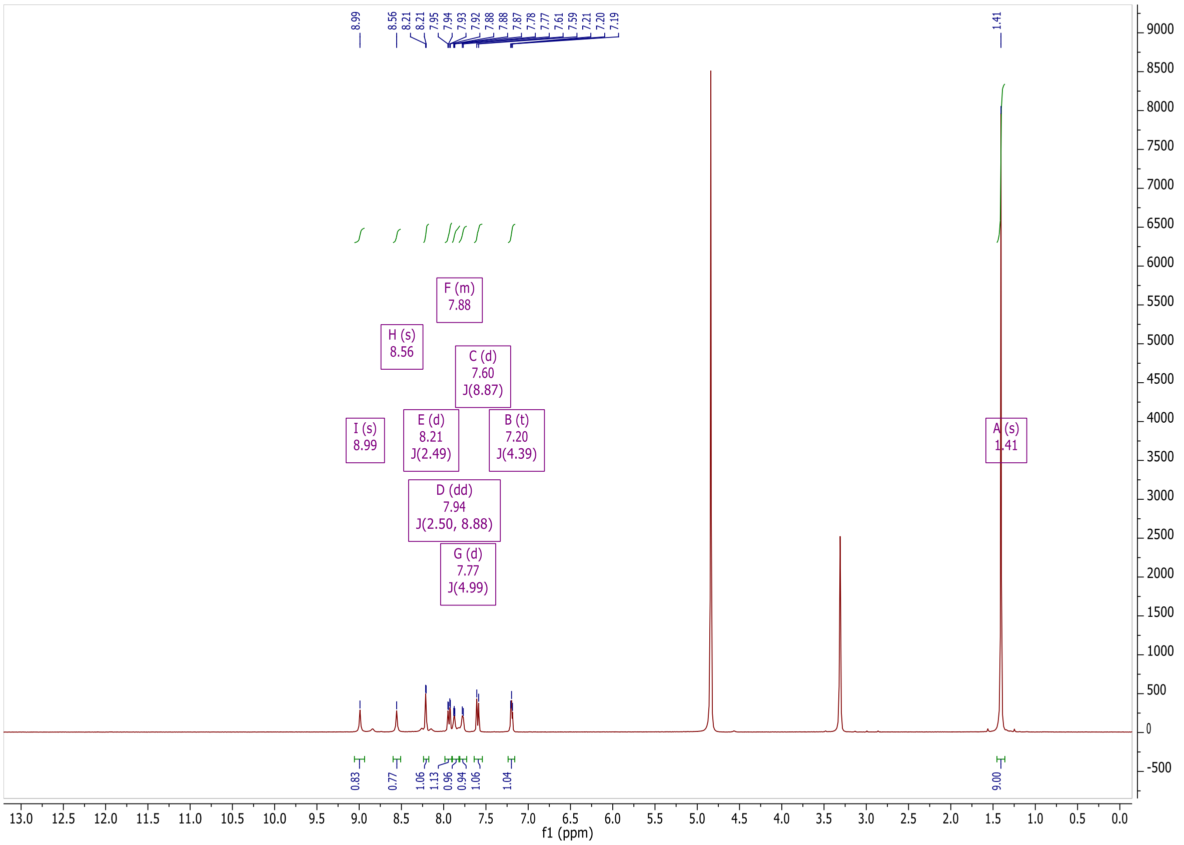

^{13}C NMR of compound **18**

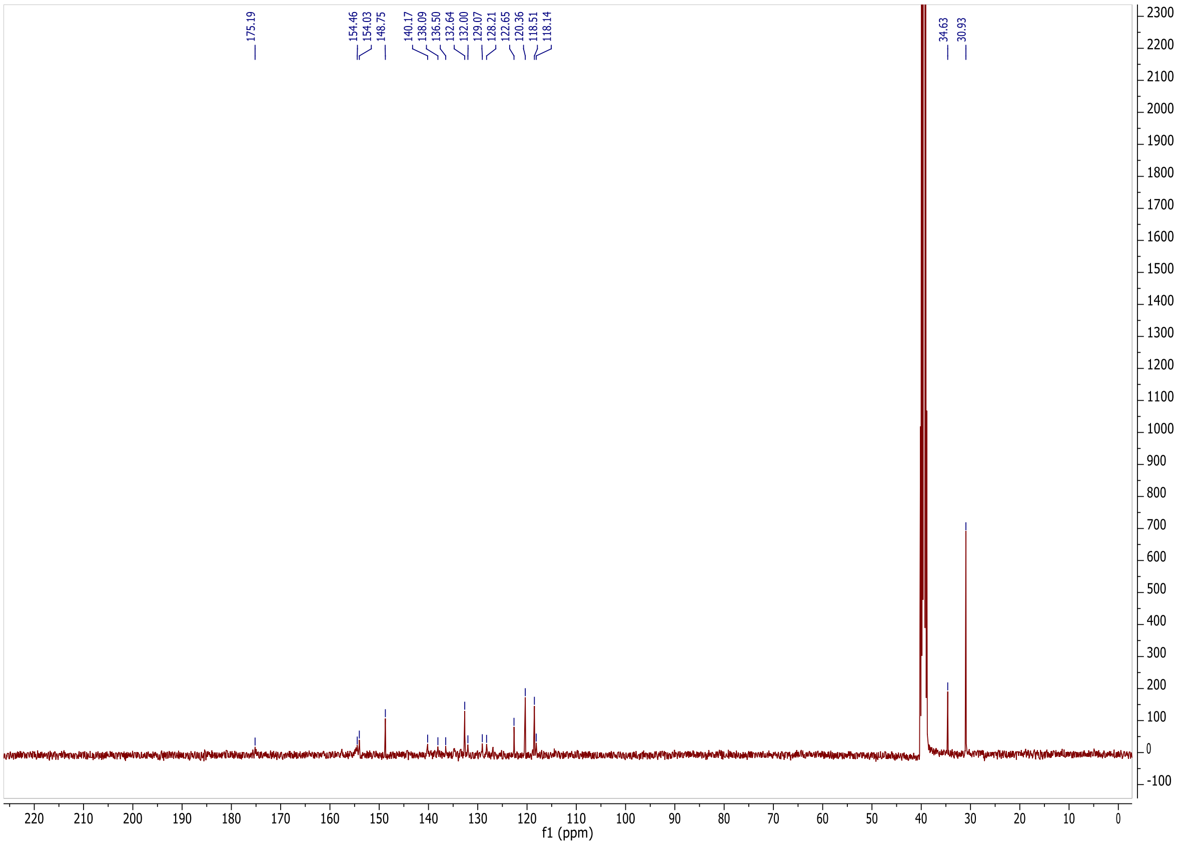

^{1}H NMR of compound **19**

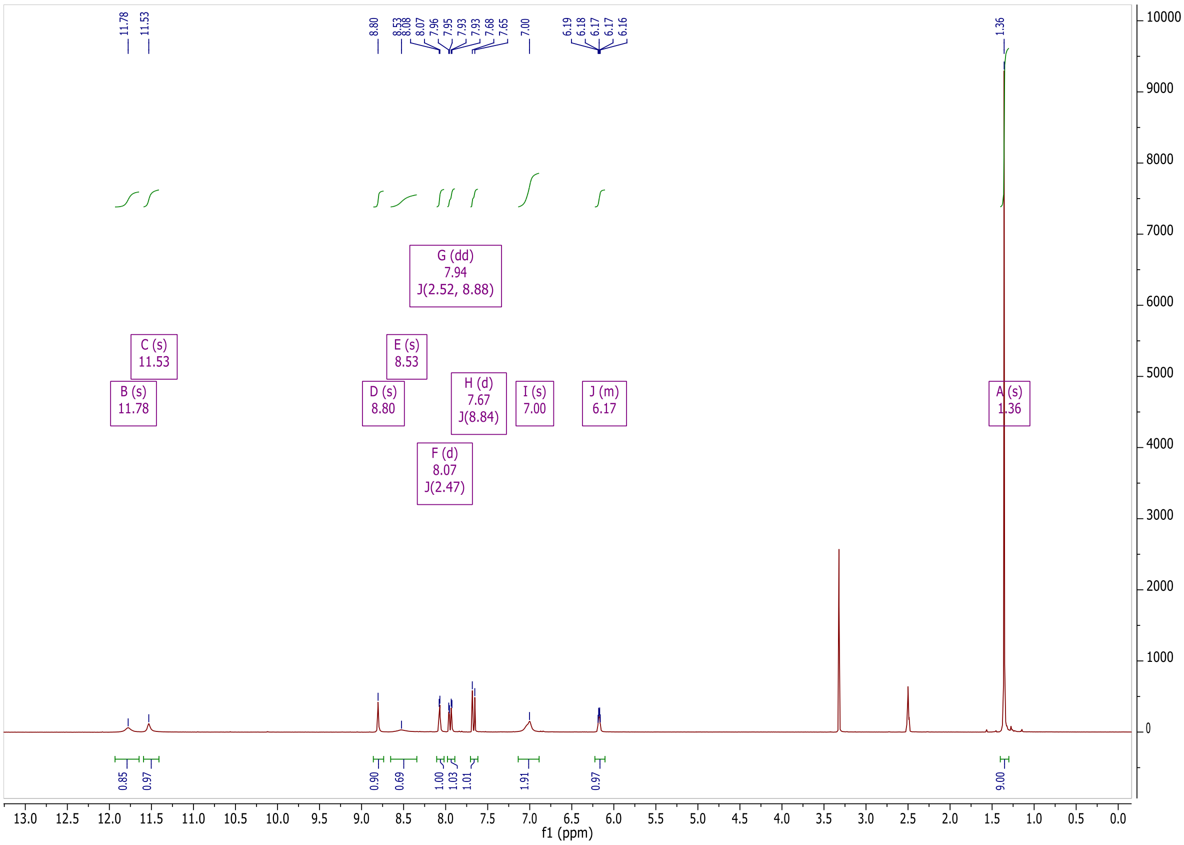

^{13}C NMR of compound **19**

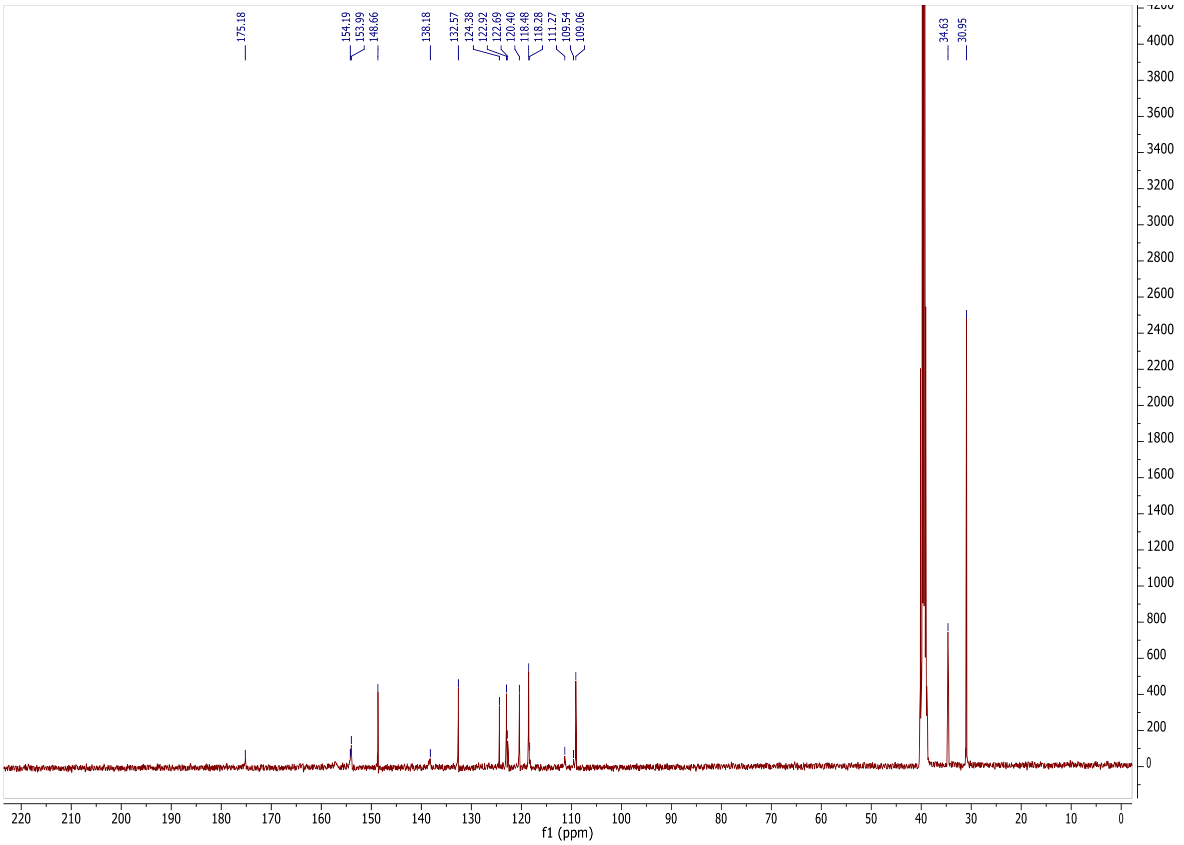

^{1}H NMR of compound **20**

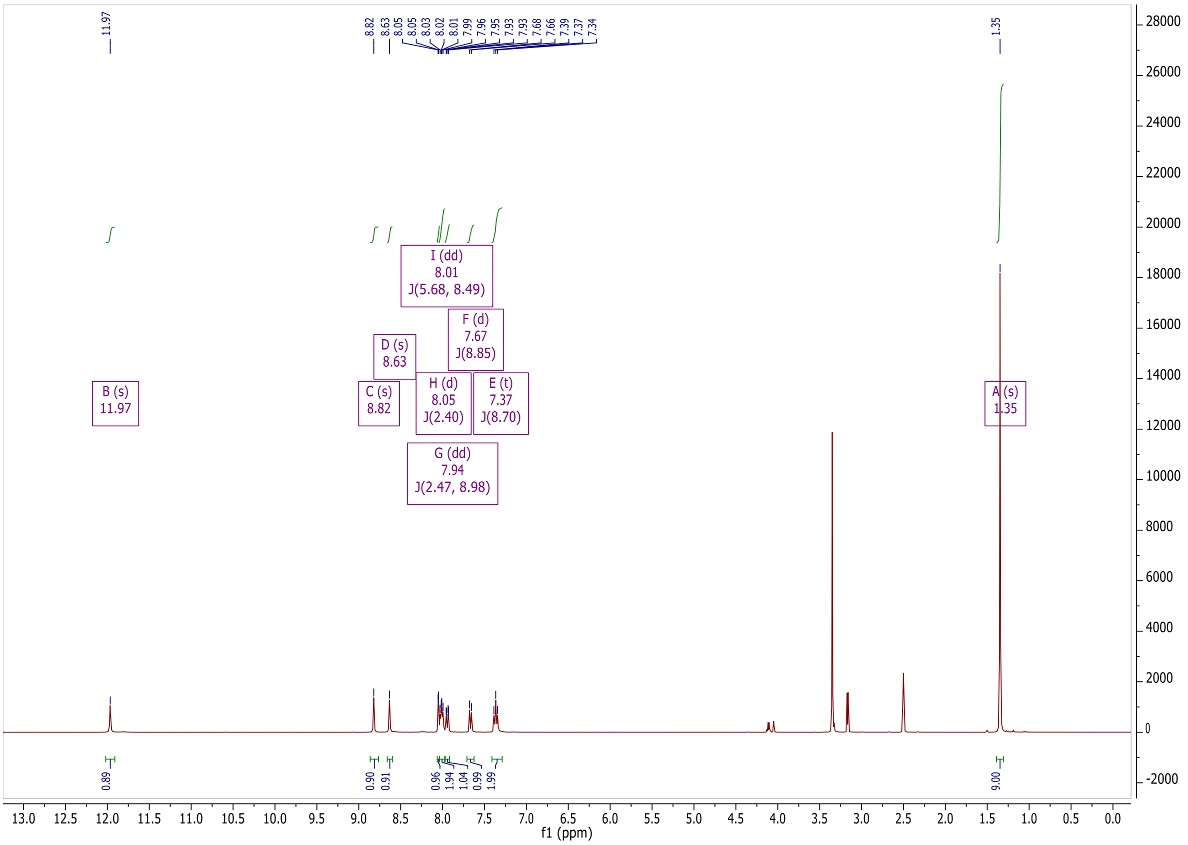

^{13}C NMR of compound **20**

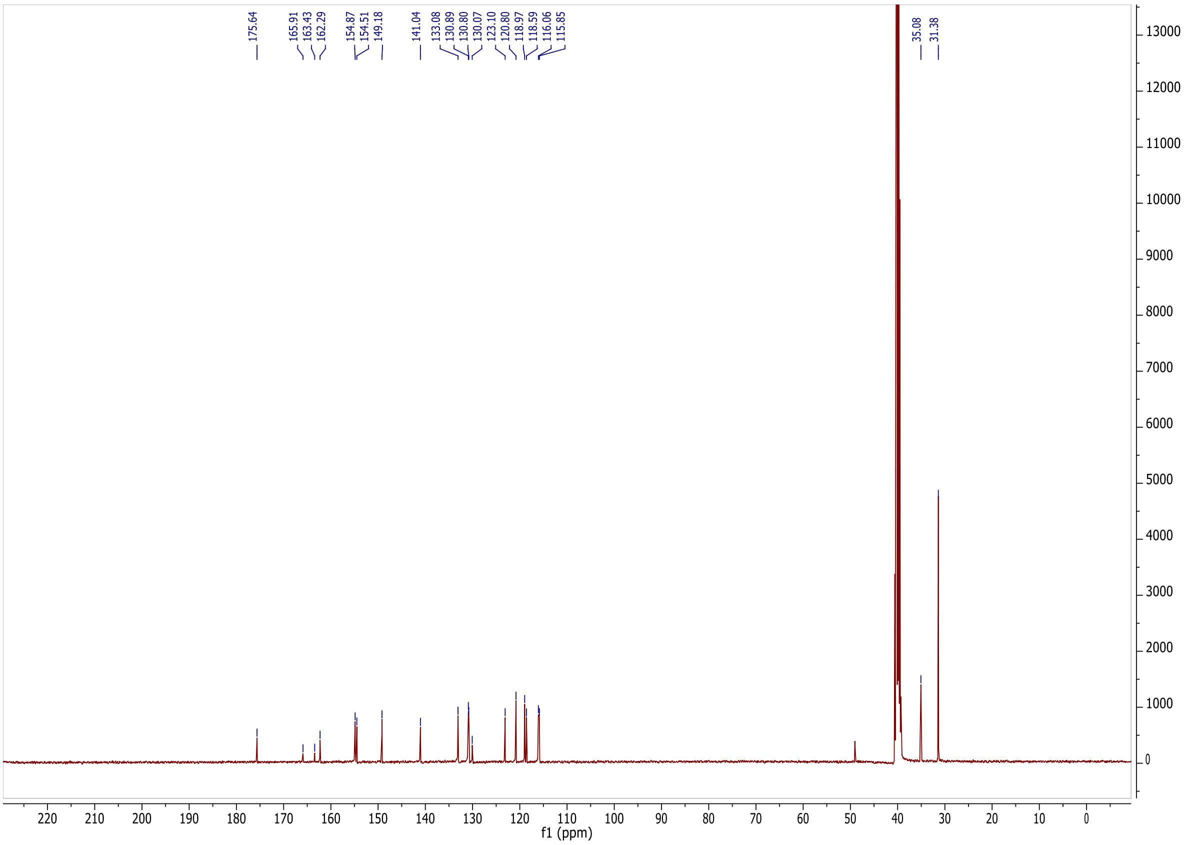

^{19}F NMR of compound **20**

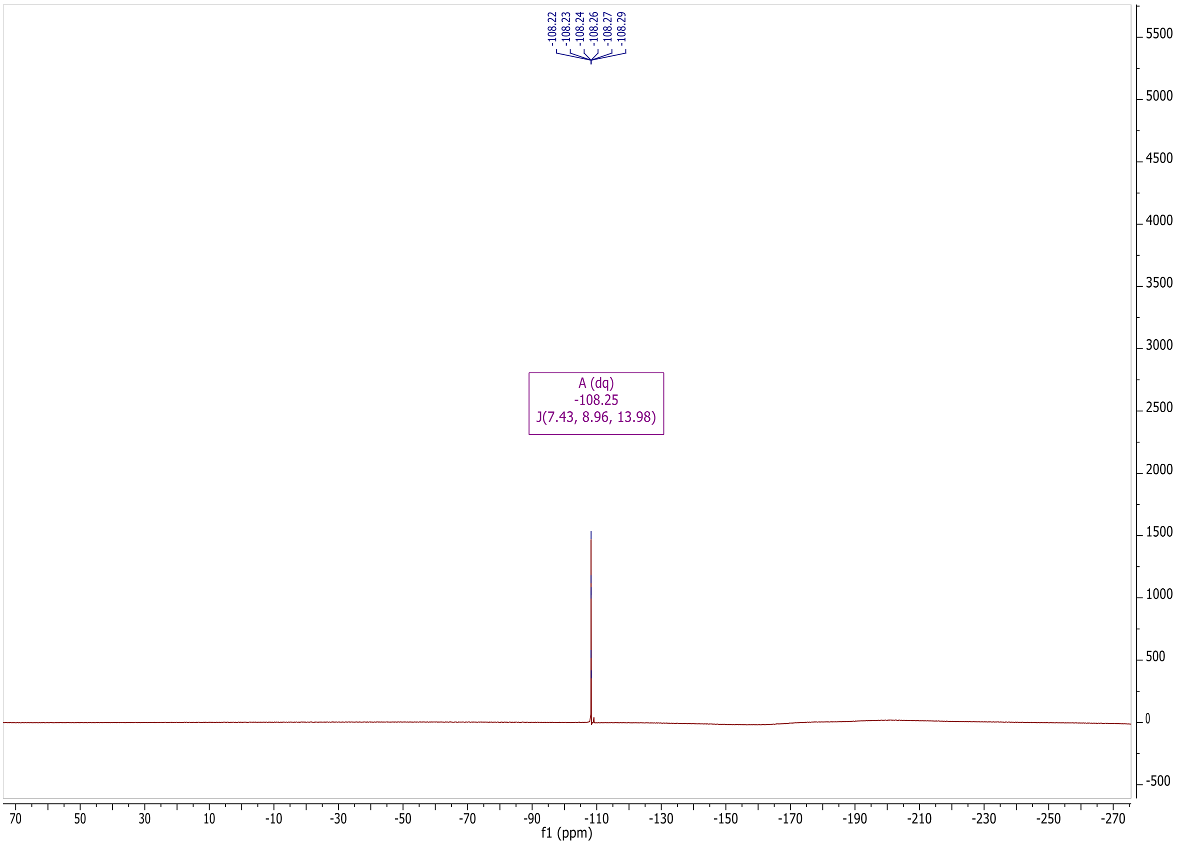

^{1}H NMR of compound **21**

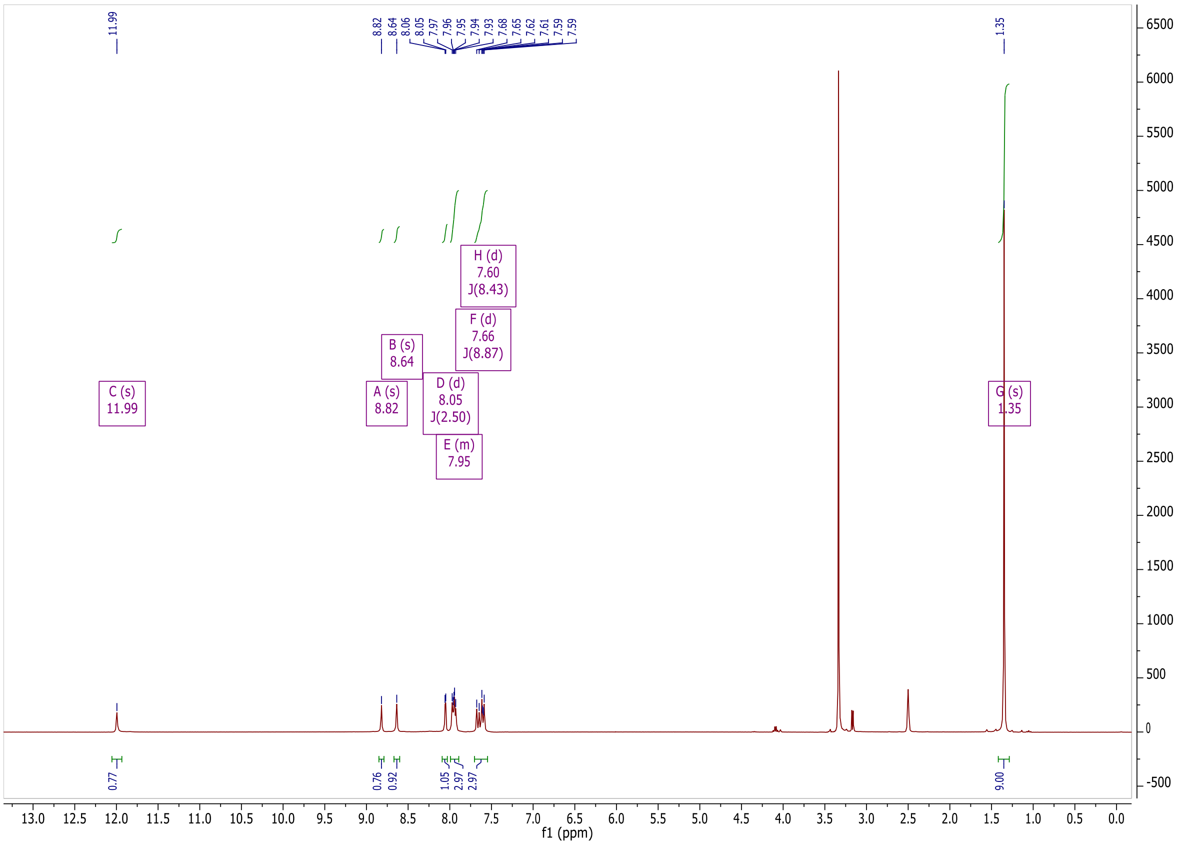

^{13}C NMR of compound **21**

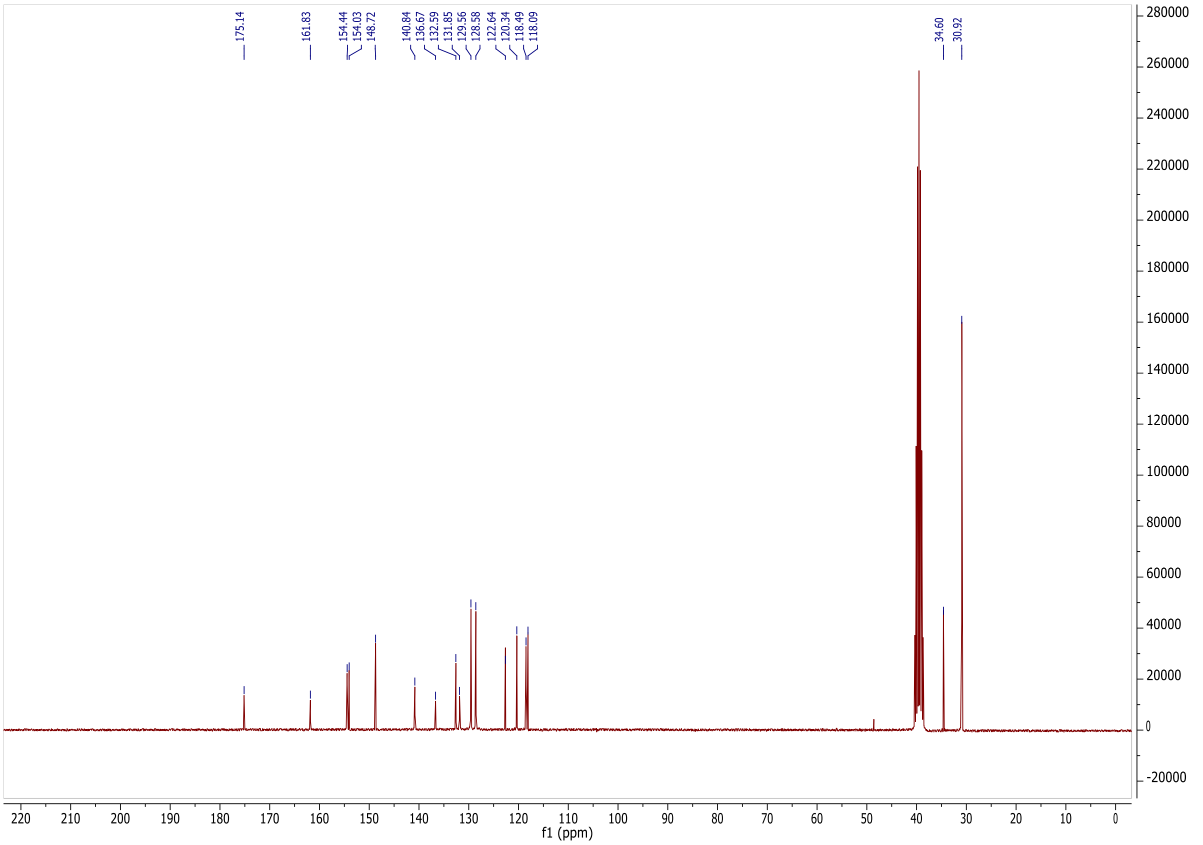

^{1}H NMR of compound **22**

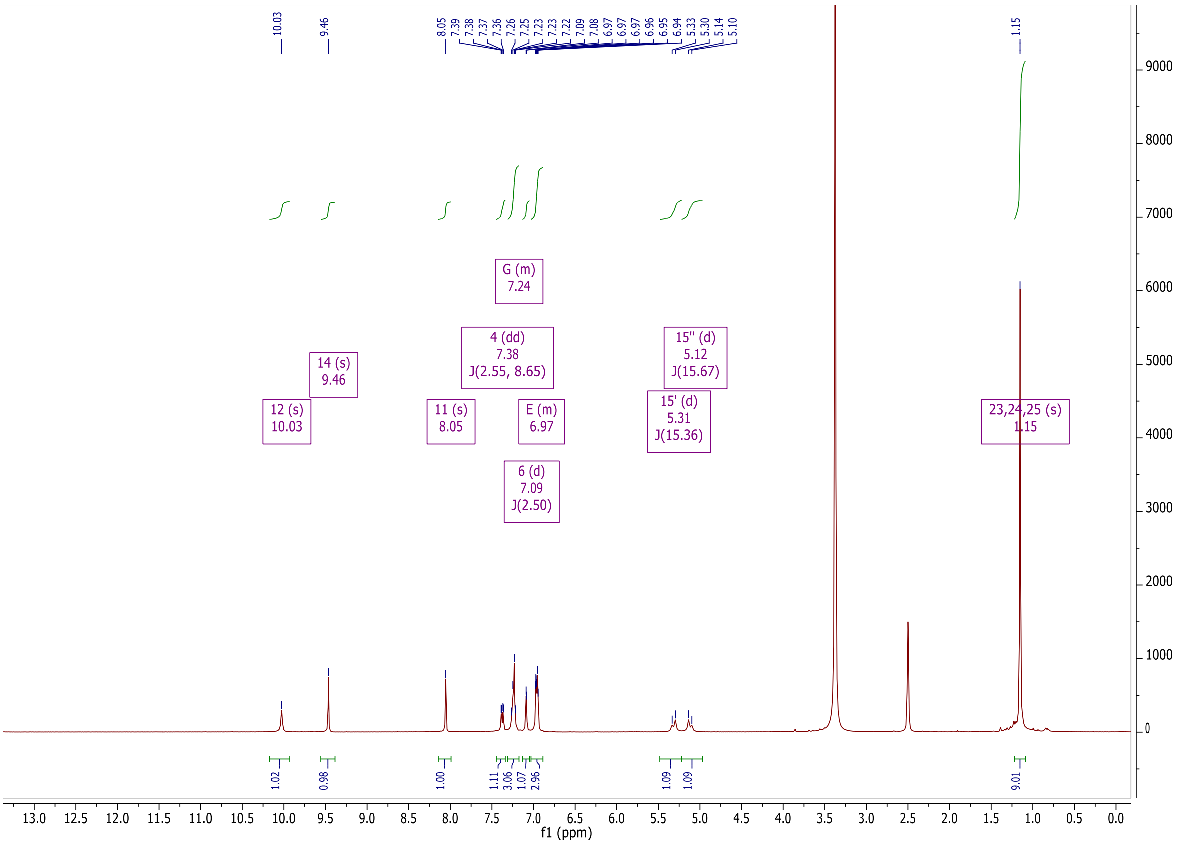

^{13}C NMR of compound **22**

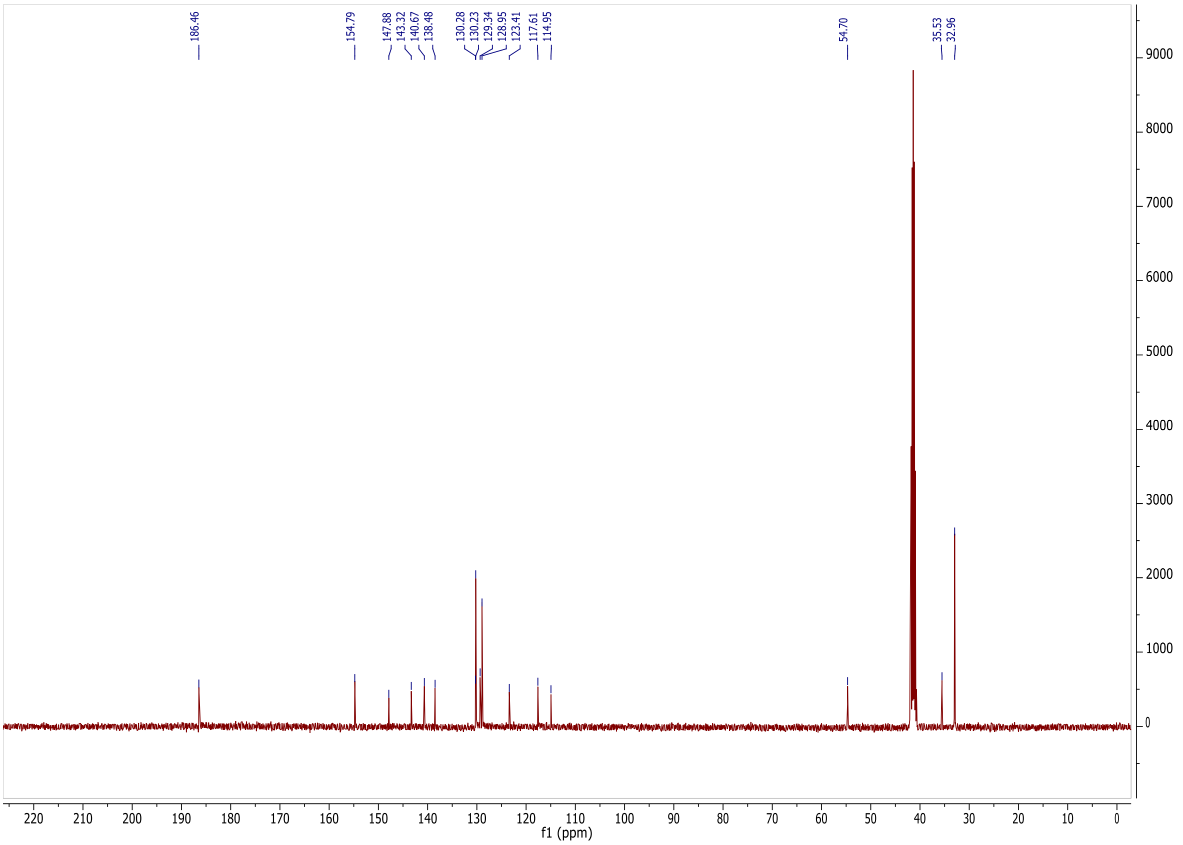

^{1}H NMR of compound **23**

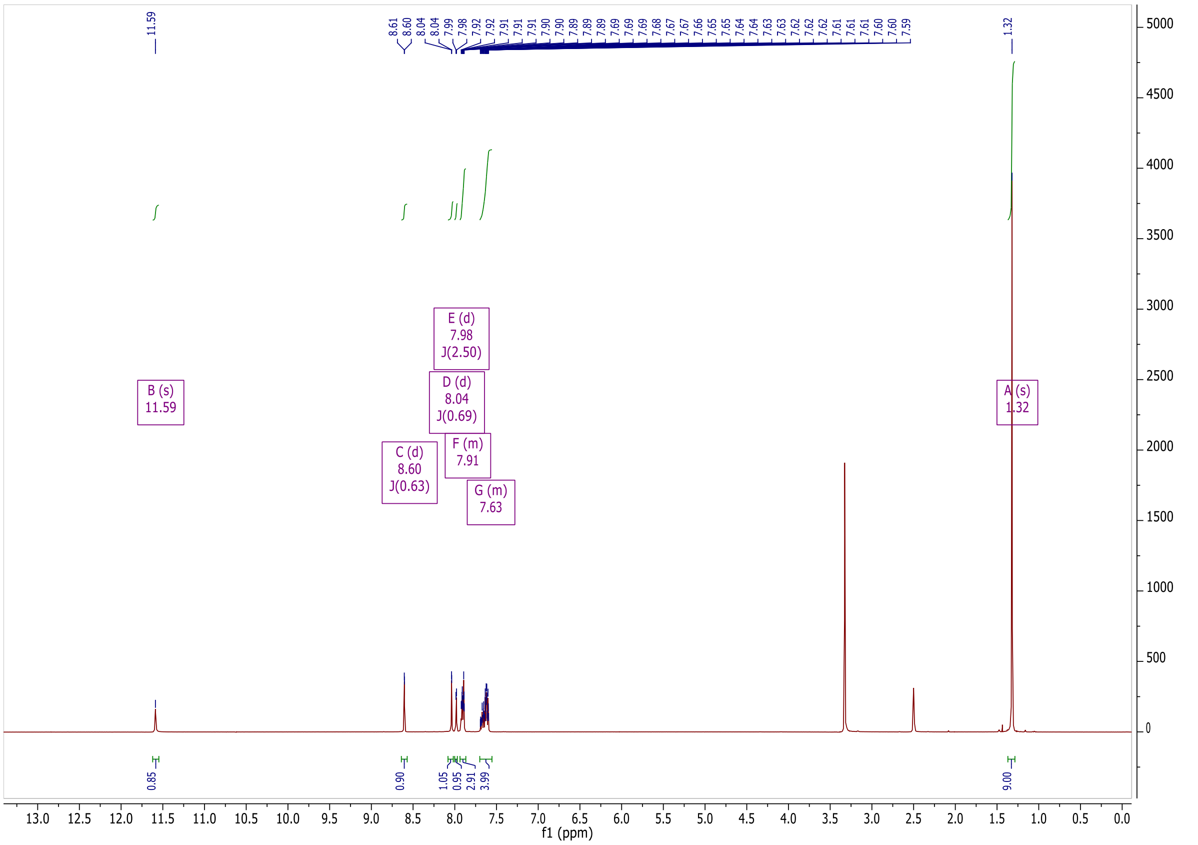

^{13}C NMR of compound **23**

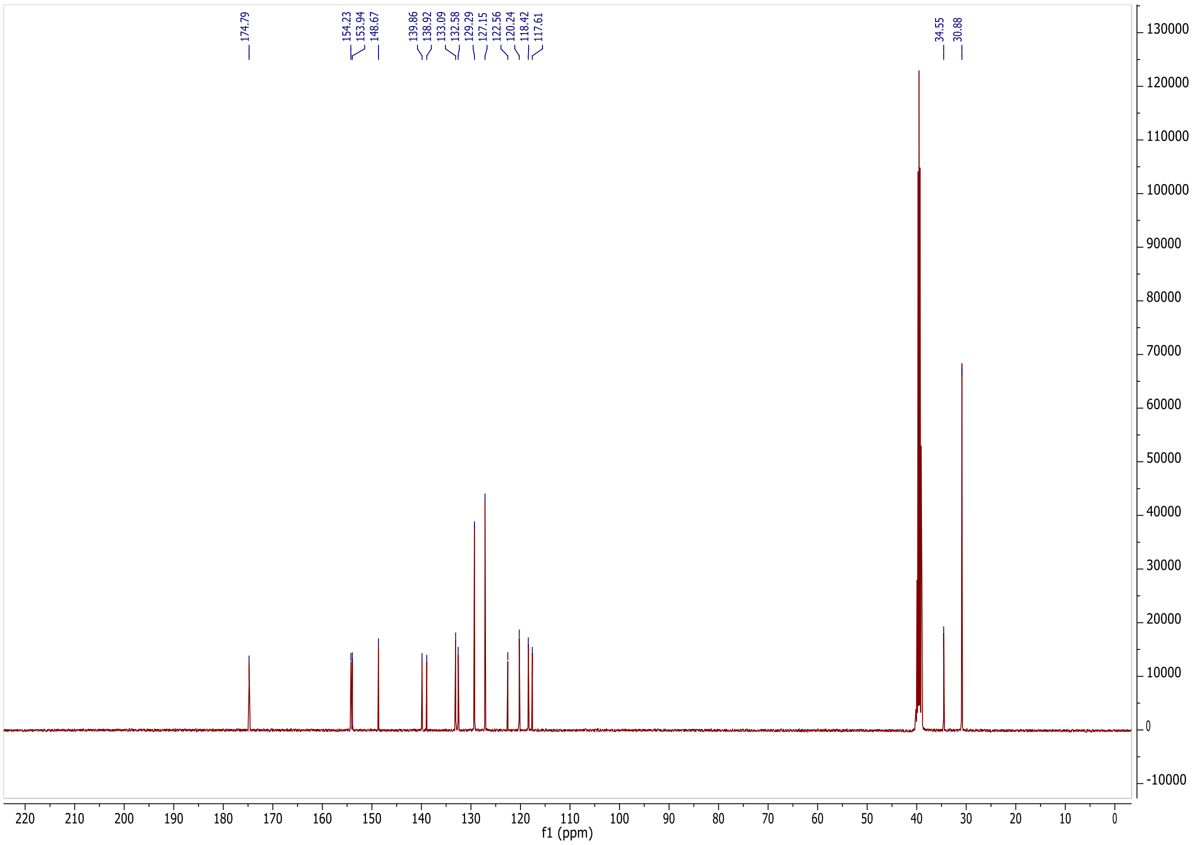

Supplement: Supplementary file 1 — Supplementary Information [file 41598_2017_17600_MOESM1_ESM.pdf]
